# The frequency architecture of brain and brain body oscillations: an analysis

**DOI:** 10.1111/ejn.14192

**Published:** 2018-10-24

**Authors:** Wolfgang Klimesch

**Affiliations:** ^1^ Centre of Cognitive Neuroscience University of Salzburg Salzburg Austria

**Keywords:** body oscillations, brain oscillations, cross‐frequency coupling, oscillatory hierarchy, phase coupling

## Abstract

Research on brain oscillations has brought up a picture of coupled oscillators. Some of the most important questions that will be analyzed are, how many frequencies are there, what are the coupling principles, what their functional meaning is, and whether body oscillations follow similar coupling principles. It is argued that physiologically, two basic coupling principles govern brain as well as body oscillations: (i) amplitude (envelope) modulation between any frequencies m and n, where the phase of the slower frequency m modulates the envelope of the faster frequency n, and (ii) phase coupling between m and n, where the frequency of n is a harmonic multiple of m. An analysis of the center frequency of traditional frequency bands and their coupling principles suggest a binary hierarchy of frequencies. This principle leads to the foundation of the binary hierarchy brain body oscillation theory. Its central hypotheses are that the frequencies of body oscillations can be predicted from brain oscillations and that brain and body oscillations are aligned to each other. The empirical evaluation of the predicted frequencies for body oscillations is discussed on the basis of findings for heart rate, heart rate variability, breathing frequencies, fluctuations in the BOLD signal, and other body oscillations. The conclusion is that brain and many body oscillations can be described by a single system, where the cross talk – reflecting communication – within and between brain and body oscillations is governed by m : n phase to envelope and phase to phase coupling.

## Introduction: Logic of the analysis

The aim of this paper is to analyze the frequency architecture of brain oscillations and to extend this analysis to body oscillations. The following questions will be addressed: How many oscillations are there, what are their frequencies, what is their functional (cognitive and physiological) meaning, what is the frequency architecture (if there is any) and, – last but not least – how are body oscillations related to brain oscillations? The line of argumentation will be briefly outlined here.

The first argument is that brain oscillations exhibit ‘preferred frequencies’. Different neurons have different preferred frequencies (Canolty *et al*., 2010), and different frequencies dominate in different brain regions (e.g., Tort *et al*., 2008). This argument, discussed in Section Not all frequencies are equal: The hypothesis of distinct frequency domains, is used to motivate the hypothesis of different ‘frequency domains’ which are associated with different‚ processing domains with respect to cognitive and physiological functions.

The second argument – which is closely related to the first argument ‐– deals with the functional role of phase and the frequency specificity of oscillations. The EEG/MEG is a complex, compound signal, consisting of a superposition of many signals stemming from different sources. When the phase of a task‐relevant oscillation is investigated, the superposition with other oscillations with different frequencies and from different sources causes serious problems. The reason is that the phase of a task‐relevant oscillation becomes distorted due to the superposition with other frequencies in a compound broad band signal. As a consequence, phase in a compound signal is meaningless. To avoid this problem, data‐driven methods for defining frequency bands and/or high‐frequency resolution methods applied to source reconstructed EEG/MEG signals can be used (e.g., Palva *et al*., 2010; Siebenhühner *et al*., 2016; Vidaurre *et al*., 2018). The important point here is that phase – in contrast to for example, power – is a highly frequency specific *and* time critical measure of an oscillation. Numerous studies have shown that phase plays a crucial role for perception, brain communication, and cognition (for reviews see e.g., Fries, 2015; Van Rullen, 2016; Palva & Palva, 2017; for a discussion of findings regarding phase for cross‐frequency coupling, see Section Principles of cross‐frequency coupling below). These findings nicely demonstrate the frequency specificity of oscillations. Or in other words, empirical findings showing that phase is meaningful also document the existence of oscillations with distinct frequencies (also termed ‘center frequencies’ in the following). This is an important conclusion, because the frequency specificity of oscillations is a crucial precondition for evaluating the question, how many oscillations are there (Section Not all frequencies are equal: The hypothesis of distinct frequency domains), and what will be the properties of the frequency architecture. This latter question refers to the frequency relationship between oscillations, and is addressed in Section Principles of cross‐frequency coupling, which emphasizes the fact that phase plays a crucial role for cross‐frequency coupling. The frequency specificity of oscillations does not mean that the frequency of an oscillation is a fixed value. Frequency varies in a state and task‐specific manner (for a recent review see e.g., Mierau *et al*., 2017) even within a very short time span (see e.g. Nelli *et al*., 2017), which may be the primary reason for the existence of frequency bands (see Sections The bandwidths of frequency domains and frequency separation and Frequency jitter and the 1/f shape of the spectrum below). Finally, it is very important to note that the frequency specificity of an oscillation can easily be blurred by averaging over trials, due to frequency shifts between trials (see Lundqvist *et al*., 2016).

The third argument refers to the numerical relationship between the frequencies of oscillations. It is an obvious fact that m : n phase to phase coupling (between a slow frequency m and a fast frequency n) is optimal for harmonic (= integer) frequency ratios only. As an example, for alpha with 10 Hz, phase coupling with neighboring frequencies is optimal for frequencies with 5 and 20 Hz which represent the center frequencies for theta and beta respectively. This is the starting point for suggesting a frequency architecture, which is based on a binary multiple frequency ratio: If a doubling/halving ratio is established between all neighboring center frequencies of traditional EEG frequency bands, a binary hierarchy of frequencies is obtained.

Based on these arguments, the binary hierarchy brain body oscillation theory is outlined in Section The binary hierarchy brain body oscillation theory, and its properties and predictions are evaluated in the following subsections. The basic assumption is that brain and body oscillations form a single hierarchy of oscillations and that center frequencies of body oscillations such as heart rate (HR) and breathing frequency (BF), but also other body oscillations, can be predicted from the center frequencies of brain oscillations.

The following Section Coupling between body and brain body oscillations deals with body oscillations and the question whether similar coupling principles can be observed between different body oscillations as well as between body and brain oscillations. It will be argued that brain and body oscillations obey the same coupling principles and can be described by a single system. The general conclusion is that the brain and the body represent a single oscillatory system with the same principles of oscillatory cross talk.

## Not all frequencies are equal: The hypothesis of distinct frequency domains

The hypothesis of distinct frequency domains assumes that in a state‐ and task‐dependent manner, different and distinct oscillations emerge. In some cases they can be detected as clear peaks in the power spectrum in other cases only by their event‐related reactivity.

Prominent examples for spectral peaks are alpha, emerging for example, during rest with eyes closed (a phenomenon known as the pioneering work of Berger, e.g., Berger, 1929), but also during different tasks demands such as attention (for a review see e.g. Foxe & Snyder, 2011) and memory demands (for reviews see e.g. Klimesch, 1999, 2012). Other examples are frontal midline theta (emerging e.g. during increased and ongoing attentional demands, see for example, Gevins *et al*., 1998; Jensen *et al*., 2002), sleep spindles (emerging after sleep onset, for a review, cf. De Gennaro & Ferrara, 2003), and slow oscillations (dominating in deep sleep, e.g. Diekelmann & Born, 2010; Staresina *et al*., 2015).

Task‐dependent oscillations were (and still are) traditionally studied by measuring event‐related changes in band power (e.g., Pfurtscheller & Aranibar, 1977; Pfurtscheller & Lopes da Silva, 1999; for a comprehensive review see e.g. Lopes da Silva, 2013). This research documents a variety of interesting properties of different frequency bands. As an example, in cognitive tasks, alpha (within a frequency range of about 8–12 Hz) is the only oscillation (in healthy humans) which typically responds with a pronounced event‐related decrease in band power, which is termed ‘event related desynchronization’ or ERD (Pfurtscheller & Aranibar, 1977; for reviews focusing on cognitive tasks, see Klimesch, 1996; Klimesch, 1999, 2012). Some task demands also elicit an event‐related increase in alpha band power, termed ‘event related synchronization’ or ERS. There is meanwhile good evidence that alpha ERD reflects cortical activation, whereas alpha ERS reflects inhibition (Klimesch *et al*., 2007a; Jensen & Mazaheri, 2010). It should be noted, however, that in movement tasks, ERD can also be observed in the beta band (a frequency range of about 16–25 Hz; e.g., Pfurtscheller & Lopes da Silva, 1999). But all other frequencies (in the delta, theta, and gamma band, with frequency ranges of about 2–4 Hz, 4–7 Hz, and 30–50 Hz respectively) typically respond with an increase in band power (ERS). Even within the alpha band, there is a clear functional differentiation between the lower and upper band (with frequencies between about 8–10 Hz and 10–12 Hz respectively). The upper alpha band responds reliably and selectively to cognitive tasks with visual stimuli (e.g., in recent research cf. e.g., Nelli *et al*., 2017; Rominger *et al*. 2018, Staudigl *et al*., 2017; Wolff *et al*. 2017), but most importantly, and independent of modality, to semantic memory demands (for reviews cf. Klimesch, 1996, 1999, 2012). The functional meaning of the lower band is less clear, but one hypothesis is that it is associated with more general attentional demands and the processing of acoustic stimuli. As an example, for a memory task with acoustically presented words, Schack & Klimesch (2002) found topographically widespread phase coupling networks during encoding in the lower alpha band only. Krause *et al*. (1999) reported that listening to music elicits a distinct reactivity also in the lower alpha band. They assume that differences between the lower and upper alpha band reflect different aspects of auditory information processing (see e.g., Krause, 1999). Another example for a frequency with a distinct function is the mu rhythm (a sensory rhythm in the alpha range that is associated with motor activity). In movement tasks, the typical finding is a decrease in mu band power (mu ERD) that co‐occurs (after a slight delay) with beta ERS, which is also termed ‘beta rebound’ (for a review see e.g., Pfurtscheller & Lopes da Silva, 1999). Theta ERS is closely associated with WM demands (Klimesch *et al*., 1994, 2001; Kahana *et al*., 1999; Kahana, 2006). The functional meaning of delta is less clear. Delta ERS may be related to very basic processing aspects, such as motivation (Knyazev, 2012), and mental concentration (Harmony, 2013), which is a typical requirement in the conscious management of different task demands. More recently, delta oscillations have been shown to play an important role for language encoding, because they are envelope coupled to speech (e.g., Giraud & Poeppel, 2012). Research on EEG resting state networks also indicates high frequency specificity (Hillebrand *et al*., 2012) in all traditional frequency bands (from delta to low gamma).

Brain stimulation studies, using repetitive transcranial magnetic stimulation (rTMS) or transcranial alternating current stimulation (tACS) also document high‐frequency specificity associated with high cognitive specificity. For rTMS at the individually adjusted upper alpha band, Klimesch *et al*. (2003) found increased cognitive (mental rotation) performance relative to sham but no effects for beta and the individually adjusted low alpha band rTMS. Similar findings were obtained by Sauseng *et al*. (2009), who observed increased performance after alpha rTMS in the retention period of a memory task. Wolinski *et al*. (2018) reported increased WM capacity with tACS at theta frequency with 4 Hz, but reduced capacity with 7 Hz tACS.

Animal research also has revealed frequency specificity even in single neurons. In the macaque cortex, Canolty *et al*. (2010) observed phase coupling networks which responded selectively to different frequencies within a range of 0.3–40 Hz. At the single neuron level, preferred frequencies were concentrated at the motor high beta band (25–40 Hz) but also at frequencies below about 1.5 Hz. Canolty *et al*. (2010) assume that different frequencies are useful to reduce interference between ensembles and that some frequencies (in this case the high beta band) are associated with a specific function of a network domain.

In summarizing, preferred frequencies with state and/or task‐specific reactivity are well documented. The hypothesis is that cognitive processing domains are associated with frequency domains represented by center frequencies of traditional frequency bands (delta 2–4 Hz, theta 4–7 or 8 Hz, alpha 8–12 Hz, beta 16–25 Hz, (low) gamma 30–50 Hz). Frequency domains form a hierarchy with optimally reduced interference and optimal coupling between domains (see Section The bandwidths of frequency domains and frequency separation below). The associated cognitive domains are language (for delta), WM (for theta), LTM which is considered a system that represents any kind of knowledge (for alpha), motor behavior (for beta), and perception (for low gamma). Higher frequencies in the gamma range are most likely not related to a specific cognitive domain. They may be associated with any type of processes, but probably more in a bottom–up‐like manner (Palva & Palva, 2017). It is important to note that the hypothesis of distinct frequency domains does not assume that any EEG/MEG frequency belongs to a certain domain. This question will be discussed later. In the following section, principles of cross‐frequency coupling will be considered that form the basis of the EEG/MEG frequency architecture.

## Principles of cross‐frequency coupling

In their review, Jensen & Colgin (2007) list four different principles of cross‐frequency m : n coupling : (i) power to power (the amplitude envelopes of m and n are correlated), (ii) phase to phase (phase coupling between m and n; also termed cross‐frequency phase synchronization), (iii) phase to frequency (phase of m is associated with a change in frequency of n), and phase to amplitude envelope coupling (phase of m is associated with an increase or decrease in the amplitude envelope of n). The best documented case of cross‐frequency coupling is phase to amplitude envelope coupling, which simply is termed ‘amplitude or envelope coupling’ in the following. The second best documented case is phase to phase coupling, simply termed ‘phase coupling’ in the following. These two cases will be discussed more closely in the next two sections.

### Amplitude (envelope) coupling

**Figure 1:**
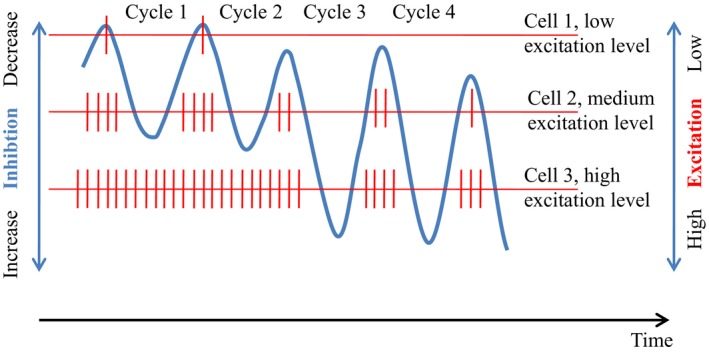
Illustration of AP modulation by theta phase. The oscillation (blue) reflects rhythmic activity in the theta frequency range of inhibitory interneurons in the rat hippocampus. The three horizontal lines with superimposed vertical lines (red) represent AP activity of three different target cells that differ in their excitation level. The horizontal lines are ordered according to the cells’ excitation level (scale on the right side) and can be interpreted as threshold. A target cell fires when inhibition (induced by the oscillation) decreases (scale on the left side) and crosses the threshold in the direction of decreasing inhibition. When the oscillation crosses the threshold in the reverse direction (toward increasing inhibition) target cells are silenced. Because the thresholds are different for the three cells (due to their different excitation levels) their temporal activation patterns are also different. Note that (i) the generation of AP's is theta phase dependent (if inhibition overrides excitation, which e.g., is not the case for cell 3 during cycle 1 and 2), (ii) particularly during cycle 3 the trough (relative to the peak) increases. This exemplifies an asymmetric oscillation which may be induced by the slope of another very slow oscillation.

The physiological function of brain oscillations is a good example for the role of phase and m : n envelope coupling. Oscillations as measured by the local field potential (LFP, i.e., the ‘EEG’ recorded from microelectrodes within the neural tissue and not from scalp electrodes as in the traditional EEG) reflect rhythmic changes in the (relative) level of depolarization in the (dendritic and somatic) membrane potentials of masses of neurons. The basic principle is that fluctuations of the LFP, which reflect phases of low vs. high excitability, modulate the firing probability for action potentials (AP's) of excitatory neurons (for reviews see e.g., Buzsáki, 2006; Klimesch *et al*., 2007a). As illustrated in Fig. 1, this can be best illustrated by an oscillation that is generated by inhibitory interneurons, a principle that is well documented for the hippocampal theta rhythm (Buzsaki *et al*., 1983; Maurer *et al*., 2006; Royer *et al*., 2012), which is the dominant oscillation in lower mammals (such as in rats) with a frequency range of about 3–12 Hz (for reviews see e.g., Lopes da Silva, 1992; Buzsaki, 2002). The point here is that even this basic and well‐established finding of AP modulation by LFP phase constitutes – in a formal sense – an example that is similar to envelope coupling if we consider the number of AP's that are triggered by phase as the ‘amplitude’ of the higher frequency which reflects AP frequency. The physiological mechanism of oscillatory AP modulation relies on a balance between excitatory and inhibitory neural activity (termed E/I balance; e.g., Atallah & Scanziani, 2009) as is shown in Fig. 1 by the differential influence of LFP phase on neurons with different levels of excitation. This oscillatory modulation of spiking is thought to reflect basic aspects of encoding and information transfer between neural ensembles (e.g., Buzsaki & Moser, 2013; Lisman & Jensen, 2013; Jensen *et al*., 2014).

**Figure 2:**
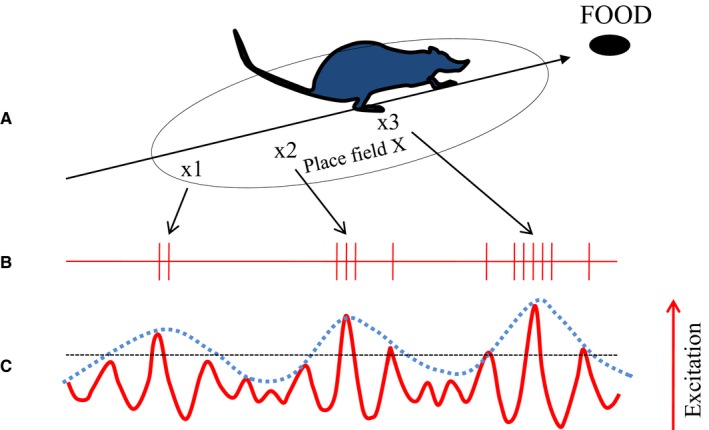
Illustration of phase envelope coupling as a memory coding mechanism. Place cell encoding can be considered an example of multiple envelope ((m_1_, m_2_) : n) coupling, where m_1_ represents gamma, m_2_ theta and n firing frequency of place cells. (A) When an animal approaches a place field, the place cell increases the number of action potentials (APs) the closer the animal is to the center of the place field. (B). The number of AP's is not increased continuously, but by increasing AP burst intensity, which is modulated by gamma phase. (C) Gamma oscillations, which drive AP bursting are in turn modulated by theta. Thus, AP bursting is double modulated by gamma and theta.

A good example for the role of cross‐frequency coupling in encoding processes comes from animal research on hippocampal place cells in rats. When an animal exhibits exploratory behavior (moving around in its environment) a dominant oscillation in the theta frequency range (about 6–12 Hz) and high activity in the gamma frequency range (about 40–80 Hz) can be observed (e.g., Buzsaki, 2002). Pioneering work by O'Keefe and colleagues has shown that different networks of CA1 hippocampal pyramidal cells respond to different places (in a maze where the rats search for food pellets). The activity of these ‘place cells’ is modulated (at least) in three different ways. They increase their firing rate, when the rat approaches a certain place field (O'Keefe & Dostrovsky, 1971; for an early summary see also Burgess & O'Keefe, 1994), as is illustrated in Fig. 2a. At the same time the firing rate is modulated by gamma, allowing action potentials (AP's) to appear primarily during the excitatory phase of gamma oscillations (cf. Fig. 2b). In addition, CA1 place cells are also modulated by theta (cf. Fig. 2c), via rhythmic inhibitory input from inhibitory interneurons oscillating at theta frequency. This theta modulation is characterized by a shift between the inhibitory theta phase and the excitatory gamma phase. As a consequence, on each successive theta cycle, the place cell fires earlier and earlier in theta phase. This phenomenon was termed phase precession (e.g., O'Keefe & Recce, 1993; Skaggs *et al*., 1996) and most likely is due to the interaction of the excitation level of the place cells with rhythmic inhibition induced by theta phase. Excitation increases, the closer the animal moves to the center of the pace field. Simultaneously, theta increases its inhibitory influence, the closer the ‘firing phase’ moves to the trough (e.g., Mehta *et al*., 2002; Magee, 2003). This increasing influence of theta‐induced inhibition counteracts the increasing excitation elicited by the movement of the rat approaching the center of the place cell. The result is a strong rhythmicity in the theta frequency range. The double modulation by gamma and theta constitutes a hierarchy of coupling between theta phase to gamma amplitude (envelope) on one hand and between gamma phase and AP firing rate on the other hand. This type of coupling is considered a basic aspect of the neural code for episodic information in working memory (for reviews, cf., Axmacher *et al*., 2010; Fell & Axmacher, 2011; Buzsaki & Moser, 2013; Lisman & Jensen, 2013; Jensen *et al*., 2014).

Lakatos *et al*. (2005) suggested the ‘oscillatory hierarchy hypothesis’ which states that the amplitudes of oscillations with higher frequencies are modulated by the phase of a lower frequency. In an auditory passive listening task with monkeys that are awake, they found that delta (1–4 Hz) phase modulates theta (4–10 Hz) amplitude, and theta phase modulates gamma (30–50 Hz) amplitude. Because this oscillatory hierarchy can entrain to the frequency of repetitive auditory stimulation, the authors assumed that the auditory cortex can adapt its temporal activity pattern in order to optimize the processing of acoustic inputs. This interpretation is fully substantiated by a variety of more recent studies on speech envelope entrainment (for a review see Giraud & Poeppel, 2012). In general, cross‐frequency coupling between the phase of a lower frequency and the amplitude (power envelope) of higher frequencies is well documented not only in animal research but also in EEG and MEG with human subjects (e.g., Vanhatalo *et al*., 2004; Mormann *et al*. 2005; for reviews see Jensen & Colgin, 2007; Canolty & Knight, 2010; Hyafil *et al*., 2015). It should also be noted that one of the oldest EEG phenomena, the waxing and waning of alpha (the coming and going of alpha ‘spindles’) obeys a similar principle. It reflects rhythmic amplitude fluctuations (Pfurtscheller, 1976) in the infra‐slow (0.01–0.1 Hz) frequency range (for a review of slow electrophysiological fluctuations see Palva & Palva, 2012a).

The conclusion is that all of these findings represent different examples of m : n phase to amplitude coupling. Even the most basic example of AP modulation by LFP oscillations (as depicted in Figs 1 and 2) reflects a principle that is similar to amplitude coupling.

### Phase coupling

Amplitude and phase coupling differ with respect to at least the following properties. (i) In a mathematical sense, amplitude coupling works for any m : n frequency ratio, but phase coupling requires a harmonic (integer) relationship between m and n. (ii) Amplitude coupling operates at the temporal precision of the slow frequency m. For phase coupling, the temporal precision is higher. It is characterized by the excitatory time window (phase) of the higher frequency n. This property and the hypothesis that slow oscillations (in the delta, theta, alpha, and beta frequency range) play an important role in cognitive top–down control (Klimesch, 2012; Palva & Palva, 2017) make it likely that m : n phase coupling (for m ≤ beta) is an important mechanism for the downstream control of neuronal synchronization in anatomically distributed neural circuits (Palva & Palva, 2012b; Fries, 2015).

For the ongoing EEG, phase to phase coupling is well documented by a variety of studies (e.g., Tass *et al*., 1998; Palva *et al*., 2005; Nikulin & Brismar, 2006; Sauseng *et al*., 2008; for a recent review see Palva & Palva, 2017). One of the basic results is that during periods of increased cognitive demands, cross‐frequency phase to phase coupling increases (Palva *et al*., 2005; Sauseng *et al*., 2008; Siebenhühner *et al*., 2016). Another basic result stems from the 1 : 2 ratio between neighboring frequencies. Center frequencies in the delta, theta, alpha, and beta band reveal a doubling/halving relationship which can in some tasks already be observed in power spectra. A good example is the co‐occurrence of frontal midline theta at about 6 Hz and upper alpha at about 12 Hz which appears during increased WM demands (e.g., Jensen *et al*., 2002). For alpha with 10 Hz and beta with 20 Hz a 1 : 2 relationship has been reported in several studies (e.g. Carlqvist *et al*., 2005; Palva *et al*., 2005; Nikulin & Brismar, 2006; Palva *et al*., 2010; Nikulin *et al*., 2012; see also Haegens *et al*., 2011 for an animal study).

**Figure 3:**
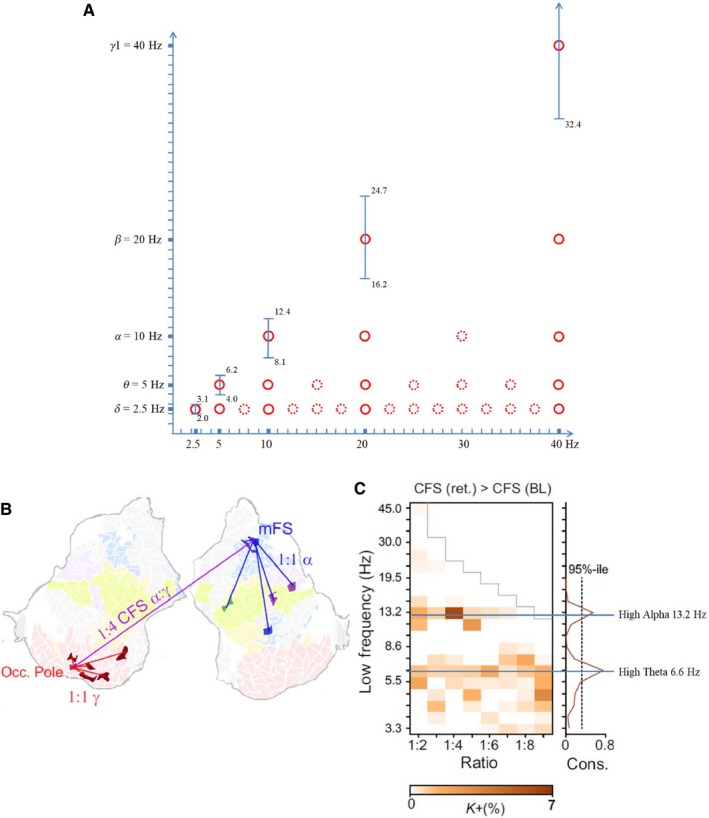
The oscillatory hierarchy is shaped by the properties of between‐frequency phase coupling. (A) Illustration of the suggested binary hierarchy. Center frequencies of traditional EEG frequencies (*y*‐axis; frequency bands, predicted by the ‘golden mean rule’ are shown as vertical bars) exhibit a 1 : 2 frequency relationship between neighboring center frequencies (solid red circles). They also couple with other frequencies at any integer (harmonic) ratios (dotted red circles). Each circle (representing phase coupling between harmonic frequencies) is hypothesized to reflect a specific brain network. This hypothesis is well substantiated by results, reported in Siebenhühner *et al*. (2016), who analyzed combined EEG–MEG signals, recorded during the retention period of a visual WM task. The example shown in (B) (from Siebenhühner *et al*., 2016; Fig. 2; reprinted with permission) exhibits three brain networks, an alpha 1 : 1, a gamma 1 : 1, and an alpha : gamma 1 : 4 network that connects the alpha and gamma networks, which are localized in the right medial frontal sulcus (MFS) and left occipital pole respectively. The example in (C) shows the increase in inter‐areal cross‐frequency phase connections during retention relative to baseline (Fig. 3 of Siebenhühner *et al*. 2016; reprinted with permission). Note the 1 : 2 ratio between high theta at 6.6 Hz and high alpha at 13.2 Hz (the inscriptions in C are made by the author and are not included in the original Fig. 3). Because theta and alpha are 1 : 2 related and because each of the two frequencies shows coupling with higher harmonic frequencies, a binary hierarchy with 6.6, 13.2, 26.4, and 52.8 Hz can be observed.

The interesting point here is that for any frequency domain, the next higher neighboring frequency domain is twice as fast. As a consequence, the ratios of all frequency domains (in ascending order), relative to the slowest domain, establish a binary hierarchy (2, 4, 8 …). However, as illustrated in Fig. 3a, this does not mean that other harmonic ratios (3, 5, 6, 7, 9, 10 ….) do not occur. The central argument regarding the binary hierarchy (doubling/halving) frequency relationship refers to the relationship between *neighboring frequency domains*. Not neighboring frequencies can couple at other harmonic ratios. As illustrated in Fig. 3a, delta with 2.5 Hz and the not neighboring frequencies of 7.5 and 15 Hz, are harmonically coupled with ratios of 1 : 3 and 1 : 6 respectively. In contrast, neither alpha nor beta (with 10 and 20 Hz respectively) can well couple with 7.5 or 15 Hz, because the frequency relationship is not harmonic.

The frequency architecture is not only defined by conditions enabling optimal phase coupling, but also by conditions enabling optimal phase decoupling to reduce interference between frequencies. Two aspects are important here. One refers to mathematical analyses which document that the golden mean (g = 1.618…..), as the ‘most irrational number’, enables the best possible frequency separation between two frequencies m and n (n/m = g; Roopun *et al*., 2008; Pletzer *et al*., 2010). Another aspect is that for frequency domains, frequency separation is provided (beside other factors) by non‐overlapping frequency bands. It will be argued below (see the Section The bandwidths of frequency domains and frequency separation) that only the binary hierarchy enables non‐overlapping frequency bands.

Empirical evidence supports certain aspects of the suggested coupling principles. One of the most sophisticated recent studies with human subjects (Siebenhühner *et al*., 2016) investigated phase coupling during the retention period of a visual working memory task from combined MEG/EEG data, which were source‐reconstructed and represented on a flattened cortical surface. Cross‐frequency and 1 : 1 within‐frequency phase coupling were measured for each pair of cortical parcels (of the Destrieux atlas) and between all frequency pairs (in the theta‐, alpha‐, beta‐, and gamma frequency range). As depicted in Fig. 3b, they found significant 1 : 4 coupling between high alpha (13 Hz) and gamma (54 Hz). The calculation of task‐related phase coupling (during retention relative to baseline) for all inter areal connections and frequency bands revealed an increase in connectivity for phase coupling of (high) theta and (high) alpha for most of the harmonic ratios (from 1 : 2 up to 1 : 9). Because theta and alpha were 1 : 2 related (with frequencies at 6.6 and 13.2 Hz; cf. Fig. 3c) and because each of the two frequencies showed coupling with higher harmonic frequencies, a binary hierarchy with 6.6 Hz, 13.2, Hz, 26.4 Hz, and 52.8 Hz can be observed. Most interestingly, Siebenhühner *et al*. (2016) also observed a task‐related decrease in connectivity, which was concentrated at around 8.6 Hz. Relative to alpha with 13.2 Hz, this frequency exhibits a ratio of 1.54, which is close to the golden mean (1.618). One may speculate that this frequency decoupling enhances the frequency separation from alpha.

Binary multiple frequency ratios (1 : 2, 1 : 4, and 1 : 8) are also reported in other studies. As an example, a 1 : 4 ratio between delta (2.2 Hz) and low alpha (9 Hz) and a 1 : 3 relationship between delta and high theta (6.7 Hz) was observed in an acoustic novelty detection task (Isler *et al*., 2008) in which cross‐frequency coupling (measured with bicoherence and crossbicoherence) was investigated. Similar preferred frequencies with a 1 : 4 ratio (between delta and low alpha) were observed for correct trials in a visual detection task (at around 2.3 and 9.5 Hz; cf. fig. 1e and fig. S2b in Helfrich *et al*. 2017). Palva *et al*. (2005) reported enhanced phase coupling for alpha with 1 : 2, 1 : 3, and 1 : 4 frequency ratios. Sauseng *et al*. (2008) found increased theta : gamma phase coupling in a visual target detection task (in frequency bands of 4–8 Hz and 30–50 Hz respectively). This finding is consistent with a 1 : 8 coupling ratio, when assuming center frequencies between 4.5–6 Hz for theta and 36–48 Hz for gamma.

It is interesting to note that in some studies, phase amplitude coupling can be observed between frequencies that represent binary multiples. As an example, Axmacher *et al*. (2010) analyzed cross‐frequency coupling in epilepsy patients from intracranial EEG recordings in the hippocampus during a visual WM task. They found increased coupling (during maintenance relative to baseline) which was largest between 7 Hz theta phase and 28 Hz beta/gamma amplitude. There also was coupling between delta (1–4 Hz) phase and beta amplitude (14–20 Hz), which, however, did not increase during maintenance. These findings show that coupling is preferably observed at binary multiples (7 Hz : 28 Hz with a ratio of 1 : 4 and ~ 2 : ~16 Hz with ratio of 1 : 8 Hz).

In rodents, theta : gamma phase coupling is well investigated, but the question here is, whether the hypothesis of distinct frequency domains is also valid for animals. Rodents (the best investigated animal species) have a dominant frequency (in the hippocampus) in the range of about 7–12 Hz which is termed theta, although in human subjects, this range represents alpha oscillations. As discussed in Section Amplitude (envelope) coupling, hippocampal theta plays an important role for spatial memory. Because rodents spend most of their time foraging and exploring their environment, which are activities that rely on memory, it is plausible to assume that hippocampal theta represents a memory‐related frequency domain. But are there also other, neighboring frequency domains similar to the hierarchy depicted in Fig. 3a? Findings reported in Belluscio *et al*. (2012), which document a complex coupling pattern between theta and different gamma frequencies, may provide an answer. These authors investigated theta to gamma amplitude and phase coupling in the rat hippocampus (CA1 region) during maze exploration (RUN condition) and REM sleep. During RUN, three gamma bands could be distinguished, slow, middle, and fast gamma (with band pass and peak frequencies of 30–50 Hz, peak: 40.5 Hz; 50–90 Hz, peak: 60.6 Hz; and 90–150 Hz; peak: 118.9 Hz respectively). The three gamma bands are associated with different phases of the theta wave. Middle gamma power was largest around the peak, slow gamma was largest on the descending phase, whereas fast gamma was most pronounced around the trough. The interesting point here is a harmonic 1 : 3 and 1 : 2 relationship of slow and middle gamma (with peak frequencies of 40.5 and 60.6 Hz respectively) relative to high gamma (with a peak frequency of 118.9 Hz): slow gamma three times and middle gamma two times equal high gamma (40.5 × 3 = 121.5 Hz, and 60.6 × 2 = 121.2 Hz ~ 118.9 Hz). It should also be noted that slow and middle gamma are frequency decoupled (60.6 Hz : 40.5 Hz = 1.5) with a ratio of 1.5 that is close to the golden mean (g = 1.618). For the analysis of theta : gamma phase coupling, a special method was used. Frequency ratios between each theta cycle (i.e., instantaneous theta frequency = 1/theta period) and the corresponding peak gamma frequency (estimated from the power spectrum between 30–90 Hz) were calculated. The findings show a more or less continuous distribution of theta : gamma ratios between about 1 : 3 up to 1 : 13 with peaks at around 1 : 5 and 1 : 9 indicating that preferentially five waves of slow gamma (8.1 Hz × 5 = 40. 5 Hz) and nine waves of middle gamma (6.7 × 9 = 60.6 Hz) are nested within one theta cycle. Fast gamma was not phase coupled to theta. These findings provide clear evidence for the existence of preferred frequencies and theta : gamma phase locking. But there is no evidence for the existence of different *frequency domains* in a binary frequency architecture. The main reason is that not in a single instance, a 1 : 2 frequency ratio was observed between theta and gamma frequencies. One may speculate that a binary frequency hierarchy is a special property of the human frequency architecture.

#### Transient event‐related phase coupling

In contrast to the ongoing EEG, the existence of phase to phase coupling in the event‐related EEG/MEG is a hotly debated issue (for a more recent study cf. Burgess, 2012). The reason is that event‐related potentials (ERPs; or event‐related fields, ERFs, in the case of magnetic signals) are traditionally used to analyze the event‐related EEG/MEG. The calculation of ERPs/ERFs is based on the more or less implicit assumption of a fixed latency and polarity‐evoked signal that appears superimposed and without interaction with the ongoing (‘background’) EEG, which is considered random noise. The ERP/ERF is obtained by averaging the EEG/MEG response for each time point over a number of single trials. Averaging aims to reduce the influence of random fluctuations of the ‘background EEG’, and allows the true signal s (i.e., the evoked response) to emerge, when the number of trials increases. But meanwhile there is convincing evidence that neither the ongoing (background) EEG/MEG is random noise, nor that the ERP/ERF is a response that does not interact with ongoing activity. On the contrary, strong evidence accumulates, suggesting that the ERP/ERF is generated by a superposition of transiently aligned oscillations (see Basar, 1999 for early work on this issue; for reviews see e.g., Klimesch *et al*., 2007b; Burgess, 2012). Single‐trial analyses demonstrate that frequencies in the theta and extended alpha frequency range (of up to 15 Hz) exhibit significant phase locking during a short‐time window (Schack & Klimesch, 2002; Klimesch *et al*., 2004; Gruber *et al*., 2005, 2014; Schack *et al*., 2005). There is also evidence that transient cross‐frequency phase coupling (in the extended alpha frequency range (of about 7–14 Hz) predicts P1 and N1 peak latencies (Gruber *et al*., 2005). Furthermore, several studies have shown that ERP components (and the P1 in particular) behave like a traveling wave (e.g., Klimesch *et al*., 2007c; Alexander *et al*., 2009, 2013; Fellinger *et al*., 2012), a finding completely inconsistent with the notion that the ERP/ERF is generated at a particular brain site by a fixed latency and polarity component. In a simulation study, Burgess (2012) has demonstrated that ERP components can be generated by a cascade of cross‐frequency phase alignments that start with high frequencies (in the gamma and beta range) thereby generating early ERP components and proceed down to low frequencies (in the theta and delta range) generating late components. In a recent study, Van der Lubbe *et al*. (2016) also conclude that at least the early ERP components such as the P1, N1, and P2 can be described as the sum of event‐related alpha and theta oscillations. These findings support the hypothesis that ERP/ERF components are generated (at least in part) by the superposition of transiently phase‐coupled oscillations. It should be noted, that a particular ERP/ERF waveform as recorded in a particular task, is not expected to comprise all possible oscillations, but only those, which are task relevant (Klimesch *et al*., 2007b).

Most ironically, if transient phase coupling between task‐relevant oscillations is a valid hypothesis (what is assumed here), ERP/ERF research is the best documented example of cross‐frequency phase coupling. The general finding is that the power spectrum of the ‘evoked EEG (i.e., of ERPs/ERFs) is dominated by traditional EEG frequencies in the delta, theta, alpha, beta, and gamma bands. Which of these frequencies are most pronounced is largely task and stimulus dependent.

This view is also supported by studies which document that pre‐ and/or peristimulus phase influence perception (e.g., Busch *et al*., 2009; Mathewson *et al*., 2009; Fiebelkorn *et al*., 2011). Fiebelkorn *et al*. (2013) have shown that the phases of different and distinct oscillations with peak frequencies at 1, 7, 9, 16, and 25 Hz are strongly associated with detection performance in a visual threshold task. The frequency ratios show two interesting properties. Each of the higher frequency (7, 9, 16, and 25 Hz) represents a harmonic multiple relative to 1 Hz. But the higher frequencies do not exhibit harmonic ratios relative to each other. A possible interpretation is that the phase of the slow frequency (transiently) drives the phases of higher frequencies which are (frequency) decoupled relative to each other.

### Interim discussion and conclusions

The basic finding is that in all examples, phase plays a crucial role for coupling. But phase can establish its impact – physiologically as well as mathematically – only, if a single oscillation is the dominant frequency in the analyzed band. The dominant oscillation may exhibit a large jitter in a broad band, as for example, is the case for hippocampal theta. However, in a broad band with different oscillations, the phases of different frequencies will tend to cancel each other. Thus, the critical role of phase for cross‐frequency coupling can be taken as strong evidence for the existence of distinct center frequencies. This conclusion, leads to the next argument, which refers to the numerical relationship between frequencies. For longer time periods, phase coupling is optimal and stable only for harmonic frequency ratios. This fact and the observation that neighboring center frequencies of traditional EEG bands exhibit a 1 : 2 ratio suggests a binary hierarchy of frequency domains. The term *frequency domain* is used to emphasize that (i) frequencies of traditional frequency bands are 1 : 2 related and (ii) reflect cognitive processing domains. The suggested frequency architecture is depicted in Fig. 3a. Frequency domains establish a binary hierarchy relative to each other but are harmonically related to other frequencies at ratios that do not belong to the subset of binary multiples. Findings from Siebenhühner *et al*. (2016) as shown in Fig. 3b provide empirical evidence for the frequency architecture as illustrated in Fig. 3a.

Finally, it should be emphasized that the requirement for a harmonic frequency relationship does not apply to phase coupling in very short‐time windows. A transient and brief phase coupling between many frequencies can occur during a short‐time window and may underlie the generation of ERP components (see e.g., Klimesch *et al*., 2007b; Burgess, 2012).

#### The bandwidths of frequency domains and frequency separation

The properties of the suggested frequency hierarchy can be derived not only from conditions providing optimal coupling. Decoupling also plays an important role as the prediction of the bandwidth of traditional frequency bands shows. Starting point is the simple fact that the golden mean provides the best possible frequency separation. As an example, for alpha, the best possible frequency separation with beta (the neighboring higher frequency domain) is 10 × 1.618 ≈ 16.2 Hz and with theta (the neighboring slower frequency domain) is 10/1.618 ≈ 6.2 Hz. Thus, 16.2 Hz may be considered the bandwidth limit of beta relative to alpha, whereas 6.2 Hz is the bandwidth limit of theta relative to alpha. On the other hand, the best possible separation of beta relative to alpha is 20/1.618 = 12.4, and of theta relative to alpha is 5 × 1.618 = 8.1. The general role for defining frequency band limits (which will be termed ‘golden mean rule’) is: Upper limit for frequency domain fd(i) is (fd(i + 1))/g and the lower limit is (fd(i‐1))*g. When applying this rule for the traditional center frequencies (fd(i); i = 1, 2 …5), we receive the following bandwidths, for delta, theta, alpha, beta, and gamma, 2.0–3.1 Hz, 4.0–6.2 Hz, 8.1–12.4 Hz, 16.2–24.7, and 32.4–49.4 respectively. It should be noted that the doubling/halving property also holds for the widths of the frequency bands which are 1.1, 2.2, 4.3, 8.5, and 17 Hz respectively (the small deviations are due to rounding). Center frequencies may shift within a band to guarantee maximal decoupling or coupling with neighboring frequency domains. As an example, alpha may shift from 10 to 8 Hz, to obtain separation from theta (8/1.618 = 5 Hz), or may stay at 10 Hz to enable optimal coupling with theta.

Only the binary hierarchy enables non‐overlapping frequency bands. If frequency domains would consist of all harmonics that can be obtained by multiplying the slowest frequency (e.g., delta with 2.5 Hz) with the integers 1, 2, 3, 4, 5, 6….. we would receive the following frequencies: 2.5, 5, 7.5, 10, 12.5, 15…. These frequencies are too densely spaced to allow the application of the golden mean rule.

Although bandwidth increases with frequency, the ratio between fd(i) and bandwidth of fd(i) stays constant for different i's. As an example for alpha with 10 Hz, bandwidth is 4.3 Hz, whereas for low gamma with 40 Hz, bandwidth is 17.2 Hz. The ratio of 10 : 4.3 and of 40 : 17.2 equals in both cases 2.33. This means that all frequency domains in the binary hierarchy are functionally equal in a sense that their frequency jitter (relative to frequency) remains the same for all frequency domains. But in absolute terms (i.e., in terms of time), jitter increases with decreasing frequency. As an example, the period of alpha is 100 ms but 25 ms for low gamma. Their jitter (calculated from their bandwidths) is (rounded) 37 and 9 ms respectively. This means that time precision (e.g., for the generation of AP's) is higher for high frequencies, but variability in time is larger for slow frequencies. Because neural encoding requires temporal variation in AP spacing (Fig. 1) a slower frequency may have a larger ‘coding capacity’ if it couples with higher frequencies. The ubiquitous observation of slow frequency phase to high frequency amplitude coupling may have to do with this fact, because the phase of a slower frequency has a stronger modulating impact (due to their larger variation in time) than the phase of a higher frequency.

#### Frequency jitter and the 1/f shape of the spectrum

One critical objection against the assumption of distinct center frequencies could be that a spectrum with several peaks for each frequency domain should be expected. If so, this would be incompatible with the well‐documented overall 1/f shape of the power spectrum. This critical issue is closely linked to four questions, to the number of different frequencies outside the hierarchy of frequency domains, to the question, when spectral peaks are expected to emerge, to the physiological meaning of frequency jitter, and to the sources of different frequencies.

As already emphasized, the assumption is not that frequency domains are the only frequencies in the hierarchy of brain oscillations, they couple with other frequencies in a task‐related way, as illustrated in Fig. 3. The emergence of peaks also is task dependent, which is well documented for theta, alpha, beta, but also state dependent during sleep as the emergence of spindles and slow frequencies (below 1 Hz) document.

**Figure 4:**
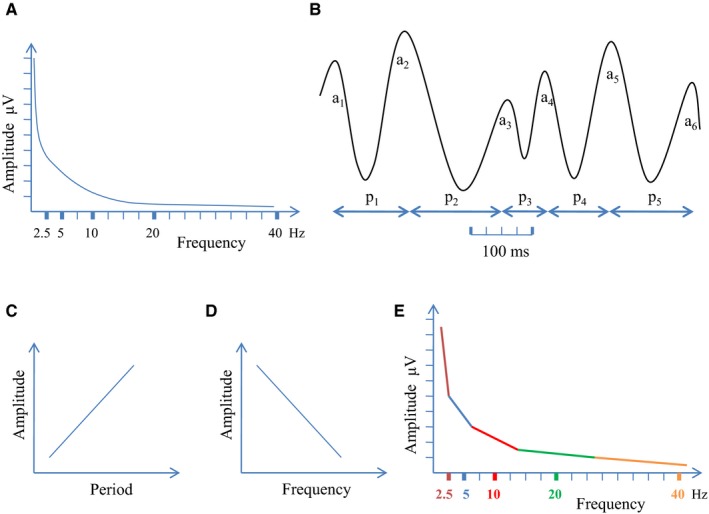
The 1/f shape of the EEG power spectrum (A) may be explained – at least in part – by cycle per cycle amplitude period co‐fluctuations of different frequencies. (B) The basic principle is that a large amplitude is associated with a lengthening of the period in the immediately following cycle and a small amplitude by a shortening of the period. As an example, amplitude a_2_ is large and therefore followed by a long period p_2_, whereas amplitude a_3_ is small and therefore followed by a short period p_3_. (C) This co‐fluctuation is characterized by a positive correlation between amplitude and period. (D) If period is expressed as (instantaneous) frequency, the correlation between (instantaneous) amplitude and frequency is negative which means that a slowing in frequency is associated with an increase in amplitude, whereas a speeding up is associated with a decrease in amplitude. (E) If a complex signal is generated by many fluctuating frequencies, its spectrum can be explained (at least in part) by amplitude period co‐fluctuations of different frequencies.

Recent research suggests that frequency jitter is not just noise, but can be explained by cycle to cycle fluctuations in (instantaneous) amplitude and (instantaneous) period (Fig. 4). Research on rat hippocampus gamma oscillations has shown that instantaneous amplitude and period (frequency) change rapidly and vary together (Whittington *et al*., 1995; Traub *et al*., 1996; Atallah & Scanziani, 2009). Most interestingly, Atallah & Scanziani (2009) could demonstrate that amplitude size predicts period (frequency) in a way that (within each cycle) an increase in instantaneous amplitude is closely associated with a lengthening of the immediately following period, and – vice versa – a decrease in amplitude is associated with a shortening in the immediately following period. This kind of cycle to cycle fluctuations is manifested by a significant positive correlation between amplitude and period (Fig. 4c), which was also found for alpha oscillations in the human EEG (Himmelstoss *et al*., 2015). According to Atallah & Scanziani (2009) the underlying physiological mechanism is due to (stimulus‐ and/or task‐ dependent) changes in excitation that are immediately and proportionally counterbalanced by inhibition. These rapid adjustments in inhibition modulate gamma oscillations over a wide frequency range on a cycle per cycle basis. These findings are in good agreement with predictions of the global wave model of Nunez & Srinivasan (2014) which assumes that the modulation density of action potentials is a function of cortical background excitability and inhibitory feedback strength. Quantification of this model predicts that an increase in parameter β (reflecting the degree of cortical background excitability) is associated with an increase in oscillatory amplitude but a decrease in frequency.

Finally, it should be emphasized that EEG/MEG signals stem from different sources in the brain. Frequencies in the delta, theta, alpha, beta, and gamma range play a primary role for long‐range connectivity (e.g., Sauseng & Klimesch, 2008; Siebenhühner *et al*., 2016; Palva & Palva, 2017) and most likely have their primary sources in the cortex (Palva *et al*., 2005; Siebenhühner *et al*., 2016). But to what extent cortical and deeper sources contribute to power spectra calculated from the scalp EEG or the MEG is an open question. This means that traditional spectral data cannot unambiguously be used to detect center frequencies, because of the existence of overlapping sources.

There are two important conclusions. First, frequency jitter (due to fluctuations in instantaneous period) is not simply noise but instead the result of a physiological mechanism that controls the relationship between excitation and inhibition. Second – and most importantly – the positive association between instantaneous amplitude and instantaneous period (Fig. 4c), which is a negative association between amplitude and frequency (Fig. 4d), is compatible with – or may even explain – the 1/f distribution between amplitude (or power) and frequency as illustrated in Fig. 4e.

## The binary hierarchy brain body oscillation theory

Starting point for the suggested theory is the already described observation that center frequencies of traditional EEG bands exhibit a doubling/halving relationship. This binary hierarchy describes a frequency relationship between any frequencies regardless of their numerical values. It represents an universal scale‐free power law. When, however, a scaling factor (s) is introduced (Klimesch, 2013), absolute frequency values can be predicted on the basis on formula (1):

(1) $$\text{fd(i)}\;=\;s*2^{i}\;\text{Hz}\;\;\;s\;=\;\text{scaling factor};i\;\ldots \ldots \;\text{integer}$$

An estimate for s can be found, when considering delta the first frequency domain fd(1). If we assume a value of 2.5 Hz for the center frequency of delta, we obtain fd(1) = 2.5 = s *2^1^. When solving for s, we receive a value of 1.25. Substituting this value in formula (1) gives formula (2):

(2) $$\text{fd(i)}\;=\;1.25*2^{i}\;\text{Hz}$$

When calculating the frequencies for the first seven frequency domains, starting with fd(i), i = 0, 1….6, we receive:

fd(0),fd(1)……fd(6)=1.25;2.5;5;10;20;40;80Hz

Three findings are interesting. First, the frequency domains fd(1)….. fd(6) describe the center frequencies of delta, theta, alpha, beta, gamma1 and gamma2 quite faithfully for s = 1.25. Second, as already mentioned, the golden mean rule also allows a faithful description of the respective bandwidths. Third, and most importantly, when asking the question, whether s itself represents a center frequency, the surprising answer is that 1.25 Hz expressed as beats per minute (bpm) equals average heart rate (HR) of adult humans which is about 75 bpm during wakeful rest (e.g., Fleming *et al*., 2011; Shaffer *et al*., 2014). Thus, if we accept this interpretation, fd(0) represents HR, which can be considered one of the most important body oscillations.

### Predictions of the theory

The predictions of the theory focus on the binary hierarchy between brain and body oscillations. This means that other harmonic ratios for coupling between *neighboring* frequency domains are excluded. As an example, a 1 : 3 hierarchy would predict frequencies that are inconsistent with empirical observations. In such a hierarchy, the neighboring frequencies of delta (with 2.5 Hz) would equal 7.5 and 0.83 Hz. In this example, we would miss theta and alpha (of about 5 and 10 Hz respectively), as well as heart rate of about 1.25 Hz.

The evaluation of the theory refers to three closely interrelated issues. The most important is the interpretation of fd(0) = HR. A second issue refers to the prediction of other body oscillations, which comprise breathing, blood pressure (BP) waves, rhythmic fluctuations in the blood oxygen level dependent (BOLD) signal, and gastric waves. The logic for the empirical evaluation is based on the ‘golden mean rule’, which allows the calculation of frequency bands. Supporting evidence is assumed, if the frequency of a body oscillation lies within the predicted band of a body oscillation. Frequency bands are, thus, used in a similar way, as confidence intervals are. A third issue is the covariation of all oscillations, brain, body, and brain body oscillations.

#### Is HR a frequency domain of the binary hierarchy of oscillations?

The measurement of individual alpha frequency (IAF), as the dominant brain oscillation, is the obvious starting point for testing the prediction that fd(0) represents individual HR. In a recent study, Gutmann *et al*. (2018) measured HR and IAF in a sample of 97 healthy young subjects during a baseline condition and after exhaustive physical exercise. Calculated from their data (Gutmann *et al*., 2018; Supporting Information), mean IAF, and mean HR were 9.87 Hz and 1.18 Hz (= 71.05 bpm) respectively. When substituting HR = 1.18 Hz for s in formula (1), the predicted frequency for IAF = 1.18 × 2^3^ = 9.44 Hz, and its bandwidth is 7.64–11.67 Hz. Thus, the empirically measured IAF with 9.87 Hz lies well within the predicted frequency band. The same logic can be applied for the prediction of HR, based on IAF. The predicted HR = IAF/2^3^ = 9.87/8 = 1.23 Hz, and its bandwidth is 1–1.52 Hz. Again, the measured value of HR = 1.18 lies well within the predicted band. The conclusion is that HR and IAF can be predicted on the basis of formula (1). Considering the large sample and the fact that subjects were controlled for age, height, weight, and body mass index (BMI) – all variables known to have a strong impact on HR (for a review cf. Valentini & Parati, 2009) – the reported findings provide strong evidence for the validity of the suggested binary hierarchy brain body oscillation theory.

#### The prediction of center frequencies in the respiratory, cardiovascular, and stomach gastric system

**Figure 5:**
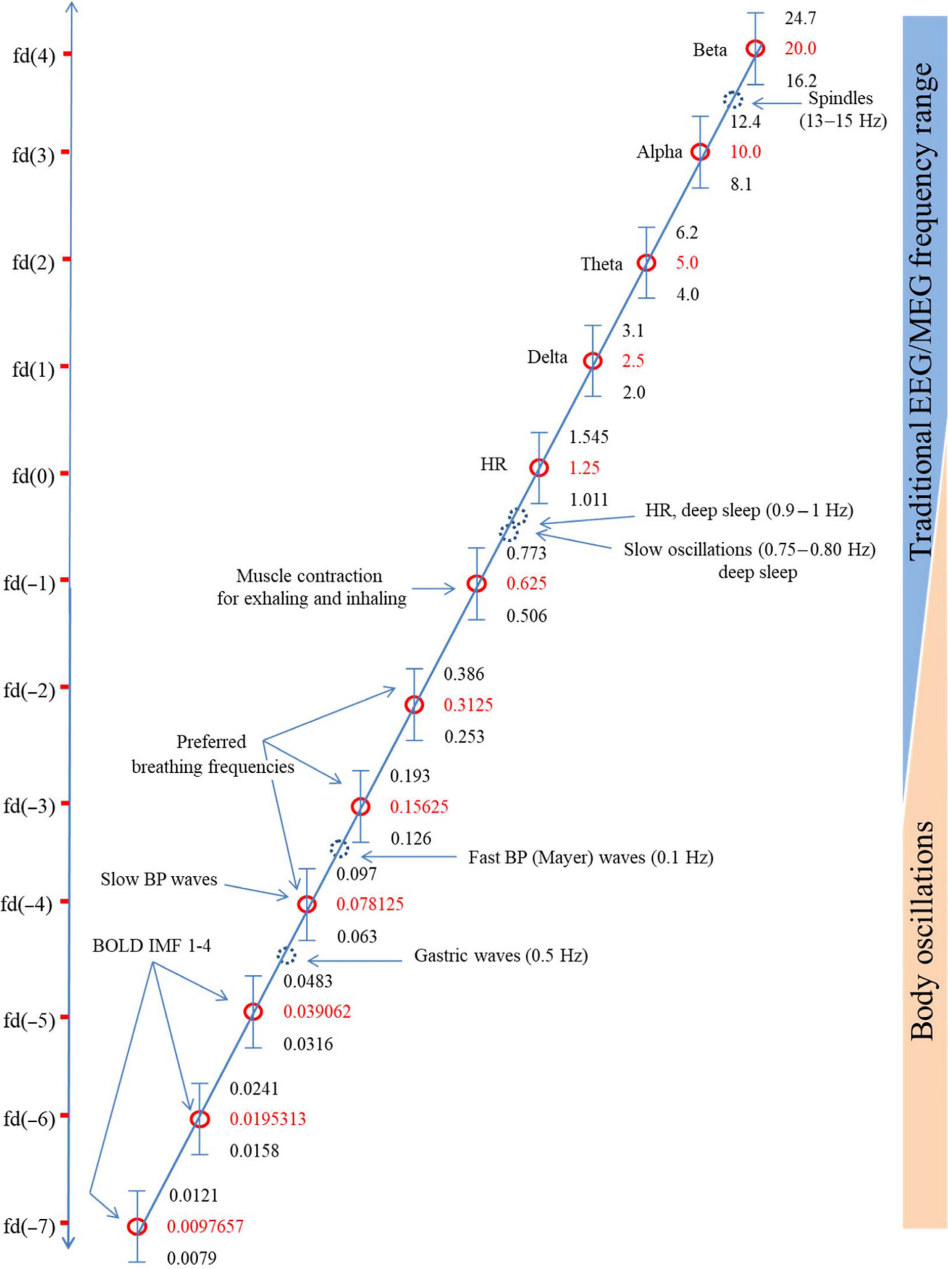
Illustration of frequency domains that are predicted by the binary hierarchy brain body oscillation theory (red circles), shown as linear function (on a log(2) scale) of frequency in Hz (*y*‐axis). Frequency bands, calculated according to the ‘golden mean rule’ (see text) are depicted as vertical bars (bandwidths relative to the *y*‐axis are not to scale). Frequencies, lying outside the predicted bands are represented as dashed blue circles and are considered falling outside the binary hierarchy. Note that dominant brain and body oscillations that emerge in deep sleep are not members of the binary hierarchy. This suggests decoupling from frequencies that dominate in the conscious, awake state.

The frequency range of fd(0) down to fd(−4) with predicted center frequencies of 1.25, 0.63, 0.31, 0.16, and 0.08 Hz are dominated by the cardiorespiratory system. We already have associated fd(0) = 1.25 Hz with HR. The next slower frequency (fd(−1) = 0.63 Hz) can be associated with muscle activity that supports breathing. Because different muscles support inhaling and exhaling, two contraction – relaxation cycles are nested within one breathing cycle. Figure 5 gives an overview of the predicted body frequencies and their relation to brain oscillations.

Breathing frequency (BF) comprises three frequency domains, fd(−2), fd(−3), and fd(−4). The crucial finding here is that BF does not vary continuously. Spectral analyses (e.g., reported in Perlitz *et al*., 2004) show distinct peaks at around 0.30, 0.15, and 0.07 Hz, which all lie well within the predicted frequency bands (0.253–0.386 Hz, 0.126–0.193 Hz, and 0.063–0.097 Hz respectively). Perlitz, Lambertz and colleagues found evidence for a distinct ‘0.15 Hz’ rhythm, which can be observed in skin blood flow as recorded by the plethysmogram (Perlitz *et al*., 2004). This rhythm emerges particulary during periods of relaxation, reflects fluctuations in vasomotor activity and – most likely – is induced by a neural pacemaker in the brainstem reticular formation (Lambertz & Langhorst, 1998; Lambertz *et al*., 2000). The interesting finding is that respiration entrains (becomes phase locked) to the 0.15 Hz rhythm at integer frequency ratios of 1 : 1, 1 : 2, or 2 : 1 (Perlitz *et al*., 2004). This means that breathing frequency exhibits a 1 : 1 or doubling/halving relationship relative to the 0.15 Hz rhythm, with dominant frequencies at 0.15, 0.30, or 0.07 Hz (cf. Table 1 in Perlitz *et al*., 2004). These findings, thus, provide solid support for the binary hierarchy theory.

**Table 1:**
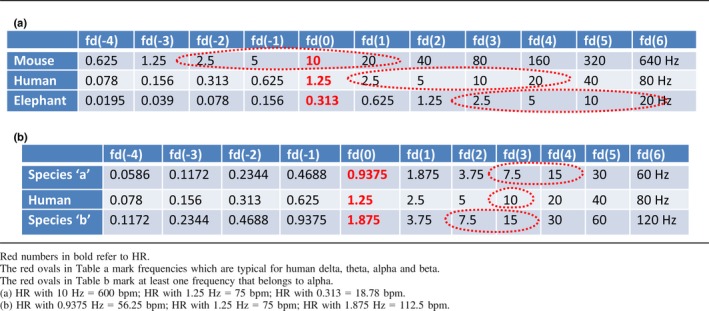
(a) Doubling/halving frequency relationship between species. (b) Maximal deviation from a doubling/halving frequency relationship between species

The slowest peak of BF at around 0.07 Hz is close to a preferred frequency of BP waves at around 0.1 Hz, which are known as Mayer waves (cf. Julien *et al*., 2001; Julien, 2006 for reviews). This frequency range is also called ‘baroreceptor range’ (e.g., Malliani *et al*., 1990), because baroreceptors detect changes in arterial BP and lead to compensatory changes in HR. Although Mayer waves exhibit a large variation, this ‘0.1 Hz rhythm’ cannot be associated with fd(−4), because it lies outside the respective frequency band (0.063–0.097 Hz). But it is important to emphasize that a faithful description of slow frequency peaks requires the application of appropriate methods. If a time series comprises two (or more) time varying components with similar wavelengths, traditional FFT analysis (which is applied in most studies) will fail to detect these components (cf. Kuusela *et al*., 2003). Applying more refined methods, including FFT analysis with sliding time windows, Kuusela *et al*. (2003) was able to demonstrate that the extended frequency range of Mayer waves, exhibits two peaks, one at 0.117 Hz (reflecting the traditional 0.1 Hz peak) and a second, slower at 0.076 Hz, which nicely coincides with the predicted frequency band (0.063–0.097 Hz) of fd(−4). The interesting point here is that fd(−4) can be associated with two body oscillations, slow BF and fast BP waves.

Finally, a more recently described body oscillation is the gastric basal rhythm, which is continuously and intrinsically generated in the stomach. It has a frequency of 0.5 Hz that can be recorded with the Electrogastrogram (EGG; cf. Koch & Stern, 2004; Rebollo *et al*., 2017; Richter *et al*. 2017). Similar to the low frequency 0.1 Hz Mayer wave peak, the frequency of gastric waves with 0.5 Hz fall outside the predicted frequency bands for fd(−4) and fd(−5) with frequency boundaries of 0.063–0.097 Hz and 0.0316–0.0483 Hz respectively. Nonetheless, it is worth noting that the 0.5 Hz gastric and 0.1 Hz Mayer waves exhibit a 1 : 2 frequency relationship.

#### The prediction of center frequencies in slow fluctuations of the BOLD signal

In a similar way as the EEG/MEG community, the brain imaging community was (and still is) primarily focusing on the event‐related response. In this sense, and despite its much lower time resolution, the BOLD signal can be considered the metabolic counterpart of the event‐related electrophysiological response as measured by the ERP/ERF. In both approaches, ongoing activity was traditionally treated as ‘background’ activity or noise. But the detection of the slow waxing and waning (fluctuation) of the BOLD signal (Cooper *et al*., 1966; Biswal *et al*., 1995; Lowe *et al*., 1998; Damoiseaux *et al*., 2006; De Luca *et al*., 2006; Mantini *et al*., 2007) enabled the investigation of resting state networks (RSNs). These are characterized by spatially coherent fluctuations of different brain regions. It is, thus, not surprising that in the neuroimaging community, the term ‘resting state networks’ is used almost synonymously with low‐frequency fluctuations (LFFs; Niazy *et al*., 2011). It became clear that the well investigated and typical event‐related BOLD response is embedded in ongoing BOLD fluctuations that are intrinsically generated by the brain in a state‐ and task‐dependent manner. Thus, in a similar way as ongoing electrophysiological oscillations, LFFs also reflect state‐ and task‐dependent and cognitive meaningful activity. As an example, the default mode network (DMN; one of the first systematically analyzed RSNs) presumably is involved in gathering information about the world around us in an ongoing manner (Raichle *et al*., 2001).

The frequency range of LFFs is determined (besides many other factors) by the temporal properties of the hemodynamic response itself, but also by the (comparatively slow) sampling characteristics of fMRI with a typical frequency resolution below about 0.5 Hz that was increased with advanced technology to about 1.5 Hz (cf. e.g., Gohel & Biswal, 2014). Many authors refer to a broad frequency range of about 0.01–0.1 Hz (e.g., Biswal *et al*., 1995; Fransson, 2005 and the review by Auer (2008), whereas others include much higher frequencies of 0.25 Hz (Balsters *et al*., 2013) or 1.5 Hz (Gohel & Biswal, 2014). Some authors divide the broad frequency range of BOLD fluctuations into four subbands (a high, two medium, and a low frequency band: 0.20 Hz–0.15 Hz; 0.15–0.10 Hz; 0.10–0.05 Hz; 0.05–0.01 Hz; cf. Baria *et al*., 2011) or in five subbands (0.75–0.5 Hz; 0.5–0.198 Hz; 0.198–0.073 Hz; 0.073–0.027 Hz; 0.027–0.01 Hz; cf. Gohel & Biswal, 2014). Others (e.g., Zuo *et al*., 2010) refer to a classification that was introduced by Penttonen & Buzsaki (2003) and Buzsáki & Draguhn (2004), who distinguish between five low frequency oscillations between 0.02 and 1.42 Hz.

If oscillatory components are not known, the definition of subbands always is a critical issue. Niazy *et al*. (2011) were the first to apply a frequency decomposition method, the empirical mode decomposition (EMD; Huang *et al*., 1998, 1999) to analyze the frequency architecture of the BOLD resting signal. This method aims to find the most dominant oscillatory components in a given time series. For fMRI data, obtained during rest and including frequencies of up to about 0.16 Hz, Niazy *et al*. (2011) found four dominant oscillatory components, which were termed intrinsic mode functions (IMFs). They comprise four frequency bands (termed band 1–4 in the following) within 0.15 and 0.004 Hz. The predicted center frequencies for fd(−4), down to fd(−7) of the binary hierarchy lie well within the extracted frequency bands, fd(−4) = 0.078 Hz lies within band 1 (0.016–0.15 Hz), fd(−5) = 0.039 Hz lies within band 2 (0.02–0.05 Hz), fd(−6) = 0.0195 lies within band 3 (0.01–0.02 Hz), and fd(−7) = 0.0097 lies within band 4 (0.004–0.01 Hz). The four bands of the IMFs show a high overlap with the predicted bands for fd(−4) to fd(−7), which are in that order: 0.063–0.097 Hz, 0.0316–0.0483 Hz, 0.0158–0.0241 Hz, and 0.0079–0.0121 Hz. The progressive decline in bandwidth from 0.134 Hz for band 1, to 0.006 Hz for band 4 also agrees well with the binary hierarchy theory.

The most important conclusion that can be drawn from the EMD analysis is that RSNs are not based on random fluctuations in the BOLD signal, but instead on oscillatory components. This conclusion rests on the following basic findings. First, it was found that the RSNs could be ‘re‐constructed’ using GLM analysis with the IMFs (i.e., the four most dominant oscillatory components) as regressors. Second, this result was also obtained, when the BOLD signal was band pass filtered (using the frequency ranges of the IMFs) before applying the independent component analysis to extract the RSNs. The RSNs appeared in all four frequency bands, but the best match with the unfiltered (original) data was in the frequency range of 0.02–0.05 Hz. With respect to the BOLD frequency bands (in the resting state) similar findings were obtained by Achard *et al*. (2006) who calculated frequency dependent correlation matrices in an attempt to depict the functional connectivity between 90 cortical and subcortical regions. This analysis resulted in a set of inter‐regional correlation matrices with each matrix describing the functional connectivity in a different frequency band. Six frequency bands were used (0.45–0.23 Hz, 0.23–011 Hz, 0.11–0.06 Hz, 0.06–0.03 Hz, 0.03–0.01 Hz, and 0.01–0.007 Hz), but functional connectivity was most salient in the frequency interval of 0.06–0.03 Hz. In an analogous way as for the study by Niazy *et al*. (2011), each of the six frequency bands reported by Achard *et al*. (2006) comprises exactly one of the six predicted center frequencies from fd(−2) down to fd(−7). This again is support for the binary hierarchy theory.

The functional meaning of LFFs and the IMFs in particular are not well understood. But it is important to emphasize that slow frequency fluctuations can also be observed in EEG direct current (DC) recordings (e.g., Monto *et al*., 2008). Palva & Palva (2012a) assume that slow EEG and BOLD fluctuations may have a common physiological source. Important evidence for this view comes from studies showing that slow EEG and BOLD fluctuations are spectrally similar (Zarahn *et al*., 1997; Monto *et al*., 2008) and that the phase of both types of slow fluctuations exhibit m : n coupling with the EEG envelope. They modulate the amplitudes of fast electrophysiological activity beyond about 1 Hz (e.g., Laufs *et al*., 2003; Mantini *et al*., 2007; Sadaghiani *et al*., 2010). In monkey cortex but also in the human brain, slow amplitude fluctuations in delta‐, theta‐, alpha‐, and gamma frequency bands are directly correlated with BOLD fluctuations (Mantini *et al*., 2007; Scholvinck *et al*., 2010).

Because alpha oscillations are the dominating frequencies in the resting EEG, several studies have used simultaneous EEG and fMRI measurements in order to directly investigate co‐fluctuations of alpha and the BOLD signal. Some of these studies found negative correlations at occipital regions, but positive correlations in the thalamus (cf. Goldman *et al*., 2002; Moosmann *et al*., 2003; Feige *et al*., 2005), whereas others did not observe negative posterior correlations but weak, non‐systematic positive thalamic correlations (e.g., Laufs *et al*., 2003). These inconsistent findings may be due to large interindividual differences in cognitive processes during rest but also to differences between the lower and upper alpha band. As has already been emphasized in Not all frequencies are equal: The hypothesis of distinct frequency domains, traditional EEG studies have shown that the lower and upper alpha band exhibit a strikingly different event‐related reactivity and topography. In a study by Jann *et al*. (2009) the lower and upper alpha band were analyzed separately and correlated with the BOLD signal. Most interestingly, the two alpha bands correlated with different RSNs. It was found that the lower alpha band is associated with the dorsal attention network, whereas the upper band is related to the DMN. The reactivity and functional meaning of the DMN and upper alpha are closely related. Both, the DMN and upper alpha are considered an internally focused state. Both signals show a similar event‐related reactivity, which is characterized by a decrease in activity during task demands and a functional association with internal processing and self‐monitoring. The functional meaning of upper alpha with respect to long‐term memory retrieval was also interpreted in terms of self‐monitoring. It was suggested that alpha enables controlled knowledge access and semantic orientation which is the ability to be consciously oriented in time, space, and context (Klimesch, 2012). This ability may be considered one of the most basic ongoing cognitive processes which become transiently disrupted during (demanding) event‐related tasks.

In summarizing, the search for slow frequencies in BOLD fluctuations showed clear oscillatory components in the frequency range of about 0.1 Hz and below (Niazy *et al*., 2011) which coincide with the predicted frequency domains fd(−4) to fd(−7). The fact that higher frequencies in the range of fd(−3) to fd(0) are not reported is primarily due to the slow sampling characteristics of traditional fMRI equipment but also to the influence of heart beat and breathing which appear in this frequency range of about 0.1–1.25 Hz and which are considered artifacts in the BOLD signal.

#### The covariation of brain and body oscillation

All oscillations belonging to the binary hierarchy are expected to covary interindividually but also intraindividually in a task‐dependent manner. As an example, if in a movement task, alpha (or more precise the mu rhythm) slows to for example, 8 Hz (see e.g., Gross *et al*., 2002) in task‐relevant brain areas, the binary hierarchy also is predicted to slow down. The problem is that – except alpha – frequency domains rarely exhibit peaks in the spectrum, which makes it difficult to calculate correlations between different frequencies. Klimesch *et al*. (1996) and Doppelmayr *et al*. (1998) used the differential event‐related reactivity of theta and alpha to test the hypothesis, whether both frequency domains are correlated between subjects. In both studies, they were able to demonstrate that the transition between alpha desynchronization and theta synchronization occurs within a narrow frequency range that varies significantly as a function of individual alpha frequency (IAF). In an animal study, Belluscio *et al*. found that during theta : gamma phase coupling, theta and gamma cycles vary together. These studies, thus, provide evidence for the covariation of brain oscillations.

Evidence for a covariation between brain and body oscillations comes from the above mentioned study from Gutmann *et al*. (2018). They found a significant increase in IAF of about 0.4 Hz after exercise relative to a baseline condition, which is paralleled by an increase in HR. This finding links two seemingly independent groups of findings. One one hand it is well documented that physical exercise has a positive influence on cognitive performance (e.g., Hillman *et al*., 2008), and on the other hand there is evidence that cognitive performance is positively correlated with IAF (e.g., Klimesch *et al*., 1990; Jin *et al*., 2006). Thus, if exercise increases IAF, cognitive performance should also be enhanced. The interesting point here is that the time course of increased cognitive performance and IAF are correlated. Positive effects of exercise on cognitive performance are most pronounced after a delay of 10–20 min (Chang *et al*., 2012) in a very similar way as IAF stays elevated in the study of Gutmann *et al*. (2018) before it drops to baseline. Evidence for a covariation between IAF and HR was also reported in a sleep study by Lechinger *et al*. (2015). During wakefulness, HR was significantly correlated with IAF, but during sleep (except REM) HR was correlated with spindle frequency. During sleep the correlation declined with increasing sleep depth, suggesting frequency decoupling of brain oscillations from HR during sleep.

It is important to note that a covariation between frequency domains is not expected in a 1 : 1 manner. As an example if HR doubles during heavy exercise relative to baseline (e.g., from 75 to 150 bpm), IAF is not expected also to double in frequency. In the Gutmann *et al*. (2018) study, the ratio between IAF to HR immediately after exercise is 10.4/3.12 = 3.33, which suggests frequency decoupling of HR from brain oscillations. This is not surprising, because immediately after exhaustive exercise cognitive performance is impaired.

## Coupling between body and brain body oscillations

In this section, some of the most prominent principles that govern coupling between body oscillations and between body and brain oscillations will be discussed. Well investigated examples are heart rate variability (HRV) and the coupling of muscle activity with brain oscillations.

### Body oscillations: m : n amplitude and phase coupling

The lung and heart are ‘mechanically’ closely coupled organs. The heart, as a ‘double’ organ, consists of a pair of chambers, the left and right atrium, and ventricle. It pumps blood (almost) synchronously into two different circuits, the lung and the body circuit. Deoxygenated blood from the body enters the right atrium, flows in the right ventricle from where it is pumped to the lungs. Oxygenated blood flows from the lungs to the left atrium, enters the left ventricle from where it is pumped through the aorta in the body arterial system (for textbooks, see e.g., Birbaumer & Schmidt, 2010).

The close relationship between the lung and the heart is functionally reflected by m : n amplitude envelope coupling in a way that the phase of the slower rhythm (breathing) modulates the ‘amplitude’ (cycle length) of the faster rhythm (heart beat). This relationship can be illustrated by a diagram, in which for each heart beat (at time *t* shown at the *x*‐axis) the corresponding cycle length (in ms) is plotted on the *y*‐axis. Fluctuations in the envelope of cycle lengths reflect m : n coupling (in this case with breathing) and is known as heart rate variability (HRV). Cycle length (or length of the period of instantaneous HR) is also termed interbeat interval and usually measured by the RR interval that is recorded with the electrocardiogram (ECG). The ECG, reflects electrical activity of heart muscle activity, and consists of a typical sequence of components. The largest component is associated with ventricular depolarization and is called R‐wave, which is embedded in the QRS wave complex. The sharp peak of the R‐wave represents a convenient trigger to measure the duration of the interbeat interval (RR interval). The RR interval can also be expressed in terms of instantaneous HR in Hz = 1000/(RR interval in ms).

Spectral analyses of fluctuations in the RR interval as measured from ongoing (continuous) recordings comprise a wide range of different frequencies between 0.4 down to < 0.0033 Hz. Four frequency bands are usually distinguished, a high, low, very low, and ultra low frequency band (termed HF, LF, VLF, and ULF with frequency ranges of roughly 0.4–0.15 Hz, 0.15–0.04 Hz, 0.04–0.003 Hz, and 0.003 Hz and slower frequencies respectively). The HF and LF bands are the most prominent frequency ranges which usually exhibit peaks at around 0.25 and 0.1 Hz (for reviews see e.g., Task‐Force: Standards of heart rate variability, 1996; Acharya *et al*., 2002). These two bands comprise the range of BF and BP waves (fd(−2), fd(−3), and fd(−4)) as described in the previous section. The m : n amplitude coupling between breathing and HR lies in this frequency range with peaks primarily around fd(−2) and fd(−3). It is known as respiratory sinus arrhythmia (RSA; for a review see e.g., Berntson *et al*., 1993) which is a cardiorespiratory coupling phenomenon characterized by RR interval fluctuations that are in phase with inhalation and exhalation (Angelone & Coulter, 1964). During inspiration HR accelerates (the RR interval decreases) and during expiration HR slows down (the RR interval increases). The amplitude (power) of the HF component depends upon breathing frequency and tidal volume (depth of ventilation; e.g., Hirsch & Bishop, 1981). It is increased during deep sleep (e.g., Bonnet & Arand, 1997; Busek *et al*., 2005) when respiration becomes deeper and more regular and decreased during REM when respiration is shallower and more frequent (e.g., Lanfranchi *et al*., 2007; Cabiddu *et al*., 2012). There is also evidence for a positive relationship between higher HF power and greater total cerebral blood flow during rest (Allen *et al*., 2015).

The LF band (0.15–0.04 Hz) comprises frequencies that stem from fluctuations in arterial BP (including the 0.1 Hz Mayer waves), whereas the VLF band (0.04–0.003 Hz) has traditionally been associated with thermoregulation and hormonal factors (Shaffer *et al*., 2014). More recent animal studies, allowing single neuron recordings from the beating heart, have shown that a VLF rhythm with a period of 90 seconds is an intrinsic rhythm that is generated by the heart itself (Armour, 2003). Several studies indicate that reduced VLF power exhibits the strongest association with mortality as compared to HRV power in higher frequency ranges (e.g., Schmidt *et al*., 2005). The ULF band with frequencies below 0.003 Hz is primarily associated with circadian fluctuations.

The important conclusion from this brief review of HRV is that m : n envelope coupling is an outstanding property of coupling between body oscillations. The general principle is that the length (‘size’) of the R‐R interval (considered formally the ‘amplitude’ of the faster oscillation) varies as a (multiple) function of the phases of (multiple) slower oscillations including respiration, BP waves, thermal, and hormonal fluctuations. But m : n envelope coupling is not the only principle. In many cases m : n phase coupling can also be observed. As an example, Bartsch *et al*. (2010, 2007) observed that heartbeats tend to cluster at a specific phase of the breathing cycle, particularly during deep sleep, and related to this finding, m : n phase synchronization between respiration and heart rate favors a 1 : 4 frequency relationship (four heart beats are phase locked within one respiration cycle), particularly during deep sleep.

Finally, it should be mentioned that fluctuations in HR can be categorized in intrinsic and event‐related frequency changes. All of the above reported fluctuations of (instantaneous) HR, are intrinsic changes that can only be seen in continuous recordings. They reflect ongoing activity and are the basis for measuring HRV. In contrast, event‐related changes are short lasting and can be observed in response to a variety of different task demands. They typically consist of an anticipatory deceleration that is followed by a stimulus/task‐related acceleration (e.g., Jennings *et al*., 1990; Pfurtscheller *et al*., 2007). Another source of event‐related changes is due to the timing of a meaningful stimulus relative to the heart beat as the work of Lacey & Lacey has shown. Stimuli appearing early in a cardiac cycle tend to prolong that cycle (Lacey & Lacey, 1980).

#### Heartbeat induced EEG amplitude and phase modulations

A special type of event‐related EEG response that reflects body–brain communication is the heartbeat‐evoked potential (HEP). It is an evoked potential, calculated time locked to the R peak (first reported in Schandry *et al*., 1986), which is used in research on interoceptive awareness. As an example, Pollatos & Schandry (2004) found that a late HEP component (between 250 and 350 ms after the R peak) was significantly higher in good heartbeat perceivers (defined as subjects capable of silently counting their heartbeat without taking their pulse). Similar findings, showing an association between cardiac awareness and HEP amplitudes were reported by Montoya *et al*. (1993) and Schandry & Weitkunat (1990). More recently, Park *et al*. (2014) have associated a HEP component (which predicts the detection of a faint visual grating) with visual awareness. Park & Tallon‐Baudry (2014) have extended this interpretation and introduced the concept of the neural subjective frame, which refers to the constant updating of internal body states.

Based on these findings, which suggest that proprioceptive information plays an important role for conscious experience, Lechinger *et al*. (2015) hypothesized that the magnitude of the HEP response will decrease with sleep depth. As expected, the results showed that HEP amplitudes and phase locking (in a time window of about 300–400 ms after the R peak) decreased during sleep, but increased again during REM sleep. Most interestingly, individual HR was significantly correlated with IAF (during wakefulness) and spindle frequency (during sleep). Because the correlation was strongest during wakefulness and declined with increasing sleep depth, this finding suggests frequency decoupling from HR during sleep.

#### A special case of m : n phase coupling: Resonance frequency breathing

Envelope coupling between BP and HR is due to the baroreflex loop. When BP increases, HR decreases and conversely, when BP decreases, HR increases. Changes in HR and BP do not occur simultaneously because of inertia in blood flow. The delay lies in a range of roughly 5 seconds and determines the resonance frequency of the hydrodynamics of the vascular system. Because the delay operates for up‐ as well as downregulations, resonance frequency has a period of about 10 seconds (i.e., a frequency of about 0.1 Hz). It should be noted that the baroreflex‐induced change in HR is almost instantaneous, but the subsequent hydrodynamic (‘mechanic’) change is delayed by about 5 seconds.

Resonance breathing can be observed, when subjects breath at the same frequency (at around 6 bpm; i.e., every 10 seconds or 0.1 Hz) as the BP (Mayer) waves. It was first observed by Vaschillo and colleagues and is characterized by five facts (Vaschillo *et al*., 2002; Lehrer, 2013), (i) The breathing‐induced RSA peak in HRV moves from the HF band to the LF band with a peak at BF (at around 0.1 Hz), where the HRV amplitude reaches a maximum, (ii) RSA and BF exhibit the same frequency (at around 0.1 Hz) and become phase locked with zero phase lag, (iii) BP waves are also phase locked, but in antiphase of 180° relative to RSA and BF, due to the delay in the baroreflex loop, (iv) HR slows down to about 1 Hz (60 bpm), and (v) the frequency at which frequency locking occurs, varies between subjects.

One central aspect of this phenomenon is that three body oscillations, BF, HRV (i.e., the RSA peak in HRV) and BP become phase locked (although with different delays) with the same frequency at around 0.1 Hz. In addition, and most importantly, HR (at around 1 Hz) exhibits a clear 10 : 1 frequency ratio relative to the frequency of BF, BP, and HRV which are phase locked to each other. This harmonic relationship also invites a phase coupling with HR (cf. Fig. 1 in Vaschillo *et al*., 2006).

What is the meaning of this phenomenon? At least three different aspects can be distinguished, one referring to energy demands, another to resonance properties, and yet another to emotional changes. Animal studies show that gas exchange in the lungs is most efficient at zero degree phase locking between HRV and breathing (Yasuma & Hayano, 2004). Thus, resonance frequency breathing represents a state, where blood oxygenation and energy demands for blood transportation are most efficient. The reduced energy demands are reflected by the decrease in HR and the antiphase BP phase locking. The latter indicates that the length of the delay in the baroreceptor loop is frequency locked to breathing and HRV (RSA). Mathematical models also suggest that resonance frequency is critically determined by the properties of feedback loops between the heart and brain (e.g., Baselli *et al*., 1994) and its frequency is around 0.1 Hz (Vaschillo *et al*., 2011). These are also dependent on body size, or more accurately, on total blood volume. Thus, it is to be expected that resonance frequency varies between subjects. This is indeed the case as the work of Vaschillo, Lehrer and colleagues have shown (cf., e.g., Lehrer, 2013). They found that taller people had slower resonance frequencies than shorter people and assumed that greater blood volume is associated with greater inertia in the blood supply, and a greater delay in the baroreceptor loop. The psychological aspect is characterized by positive emotions, reduced stress, and is associated with the appearance of very rhythmic (sinusoidal) waveforms of HRV at around 0.1 Hz. Biofeedback with the aim to enhance 0.1 Hz HRV is known to induce relaxation (McCraty *et al*., 2009; Lehrer & Eddie, 2013; Lehrer & Gevirtz, 2014; Gross *et al*., 2016).

As already emphasized, 0.1 Hz waves do not belong to the binary hierarchy. Thus, the tight coupling between three 0.1 Hz waves (BF, HRV and BP) and the 1 : 10 ratio with HR, implies a decoupling from the binary hierarchy. Because the binary hierarchy is thought to reflect a system for optimal coupling between brain and body oscillation, resonance breathing may be considered a situation, where body oscillations are entrained to the resonance properties of the cardiorespiratory system, but are only weakly coupled with brain oscillations.

### Gastric waves and body brain coupling

An interesting case of body–brain coupling was reported by Richter *et al*. (2017), who simultaneously recorded the EGG and MEG in resting subjects. Proceeding from the hypothesis that the gastric basal rhythm may influence resting state brain dynamics, they calculated phase amplitude coupling between gastric waves and alpha amplitudes. They found that gastric phase accounts for about 8% of alpha amplitude fluctuations. Directionality analyses suggest an ascending influence from the stomach to the brain. Rebollo *et al*. (2018) extended this finding in a BOLD study and showed that a brain network (termed gastric network) is phase synchronized with the gastric basal rhythm. Within this gastric network, approximately 15% of the BOLD variance is explained by gastric phase.

### The motor system and brain body coupling

Muscle activity comprises a wide range of oscillatory components. It will be argued that muscle frequencies are phase locked to the phase of EEG frequencies. It will further be shown that limb resonance frequency is an important factor for the motor frequency architecture.

#### Muscle oscillations and tremor frequency

Muscle activity usually is monitored by the use of electromyography (EMG). The EMG primarily reflects properties of motor unit action potentials (for a detailed discussion see e.g., Hermens *et al*., 1992). Frequency analyses show a flat double ramp‐like shape of the spectrum, with an ascending part up to a broad mean frequency range of about 40–70 Hz, which is followed by a descending part in a high frequency range that extends to several 100 Hz. The fact that the EMG has dominant power in the gamma frequency range plays an important role for the evaluation of muscle artifacts, when analyzing EEG/EMG gamma activity (for a review see e.g., Muthukumaraswamy, 2013).

Shifts in EMG mean frequency are considered valid indicators of muscle fatigue. An increase in fatigue is reflected by a downward shift in the EMG frequency spectrum, which is characterized by a relative decrease in amplitude in the higher frequency range and a small increase in the slow frequency range (for a review see Phinyomark *et al*., 2014). In studies on mean EMG frequency, the recording usually is done under different muscle force levels when limbs are not moved. The shape of the EMG spectrum is influenced by a variety of factors, primarily by the type of muscle, task, and recording (surface or needle electrodes).

For EMG frequencies in the beta and gamma range (of about 15–30 Hz and 30–60 Hz respectively), coupling between the motor cortex and muscles is well documented in contraction tasks (e.g., Conway *et al*., 1995; Salenius *et al*., 1997; Brown *et al*., 1998; Halliday *et al*., 1998; Gross *et al*., 2000 see e.g., Grosse *et al*., 2002 for a review). In the gamma band coherence is found for contraction and movement tasks. But in the beta band, coherence may be abolished during movements (e.g., Kilner *et al*., 1999).

**Figure 6:**
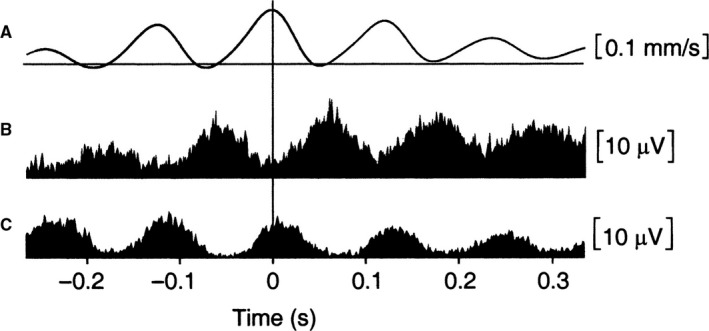
Body oscillations: An example for envelope and phase coupling in the movement system from Gross *et al*. (2002), reprinted with permission. Copyright (2002) National Academy of Sciences, USA. The signal trace in (A) is an example for the velocity (micromovement) signal, recorded (by an ultrasound localization device) from the tip of the right index finger. This signal reflects physiological tremor with a peak frequency of about 8 Hz. The averaged EMG traces for the flexor and extensor muscles are shown in (B) and (C) respectively. Maxima in the velocity signal were taken as triggers for averaging. Note phase coupling between the 8 Hz tremor and the respective 8 Hz envelopes in the EMG traces. Tremor is in phase with the extensor but antiphase with the flexor muscles.

Most importantly, during actual movement, rhythmic busting of the EMG signal can be observed in slower frequencies from delta to slow beta. This is another example of an amplitude envelope modulation (in this case of the EMG) by slower frequencies (e.g., in the alpha frequency range, cf. Vallbo & Wessberg, 1993; Wessberg & Vallbo, 1995; Mehrkanoon *et al*., 2014; or in the delta range, e.g., DeLuca & Erim, 1994; Ruspantini *et al*., 2012). It is interesting to note that this frequency range from slow beta down to delta overlaps with the frequency range of physiological and pathological tremor. Tremor is defined as a rapid back and forth movement of a body part. It reflects a mechanical signal that usually is termed velocity signal (e.g., Vallbo & Wessberg, 1993; McAuley & Marsden, 2000) but also kinematic or accelometric signal. As mentioned above, the EMG exhibits a broad frequency range, but the rhythmic ‘bursting envelope’ of the EMG observed during slow movements represents tremor frequency as shown in Fig. 6.

In a study with normal subjects, Gross *et al*. (2002) have shown that the ~8 Hz EMG (and velocity) signal is significantly coherent with the cortical MEG signal in the primary motor and sensory cortex (M1 and S1 respectively). Directionality analyses revealed an efferent drive from M1 to muscle, but an afferent input from muscle to S1. These findings are good examples of phase synchronization in a complex brain network reflecting long‐range neural communication (cf. also Schnitzler & Gross, 2005). Together with the EMG results these findings suggest that slow movements consist of successive micromovements (where an agonist burst is followed by an antagonist burst; for a review see e.g., McAuley & Marsden, 2000). Gross *et al*. (2002) conclude that ~8 Hz oscillations ‘represent the neural mechanism of intermittent motor control, providing common timing for synergistic muscles, which ensures constant duration of micromovements across muscles’. In other words, micromovements in muscle fibers of different muscles have a similar time constant, allowing for a ~8 Hz synchronization (phase locking) between them. These synchronized micromovements are the source of tremor. The efferent drive from M1 suggests a cortical contribution to the generation of tremor. Thus, tremor is not an epiphenomenon of peripheral muscle unit activity, but an integral feature of sensory motor control in a complex corticomuscular loop.

A variety of studies has shown that tremor in a frequency range overlapping the alpha band comprises a central (i.e., cortical) component (e.g., Raethjen *et al*., 2002, 2007; Budini *et al*., 2014). This has also been shown for pathological tremor (cf. McAuley & Marsden, 2000 for a review). Tremor frequency is not only determined by the frequency of cortical oscillations, but also by peripheral properties, such as corticomuscular and musculo‐cortical delay times (which are about 15 ms and 20 ms respectively; cf. Govindan *et al*., 2005), the spinal stretch reflex loop (operating as an inhibitory feedback loop) and the resonance frequency of the limbs. The latter not only is determined by limb length (in a similar way as pendulum length determines frequency) but also muscle stiffness, and load. Thus, a straight forward empirical approach to dissociate the influence of central (cortical) from peripheral factors on tremor frequency is to measure tremor under different load conditions. As an example, Raethjen *et al*. (2002) found that the frequency of significant 6–15 Hz corticomuscular coherence did not change under increased load and, thus demonstrated the importance of a central drive. The influence from peripheral factors comes for example, from findings showing that patients with neurological lesions resulting in deafferentation (which removes the contribution of reflex loops) have preserved but less sharply tuned 10 Hz frequency range tremor (Marsden *et al*., 1967).

The finding of a more or less stable corticomuscular coherence in a frequency range that overlaps with the alpha band is interesting because resonance frequency of different body parts varies to a very large degree. Resonance frequency is that frequency at which – due to biomechanical factors – body parts have a preferred tendency to move. As an example, for the unloaded finger, resonance frequency is around 25–27 Hz (Stiles & Randall, 1967). For the longer body parts, such as the wrist, elbow, arm, and leg the respective frequencies are at around, 9, 2, 0.98, and 0.85 Hz respectively (Marsden, 1984; Wagenaar & van Emmerik, 2000).

As a working hypothesis, it may be suggested that slow frequencies that modulate the envelope of the EMG have (at least) three sources, a central micromovement, a peripheral resonance, and a macromovement control source. The central source is primarily associated with neural mechanisms of intermittent motor control as suggested for example, by Gross *et al*. (2002). The peripheral source is associated primarily with the resonance frequency of the different body parts, whereas macromovement control may be associated with the respective regions in the cortex and basal ganglia.

### Body movement oscillations with a 1 : 1 and doubling halving frequency ratio

**Figure 7:**
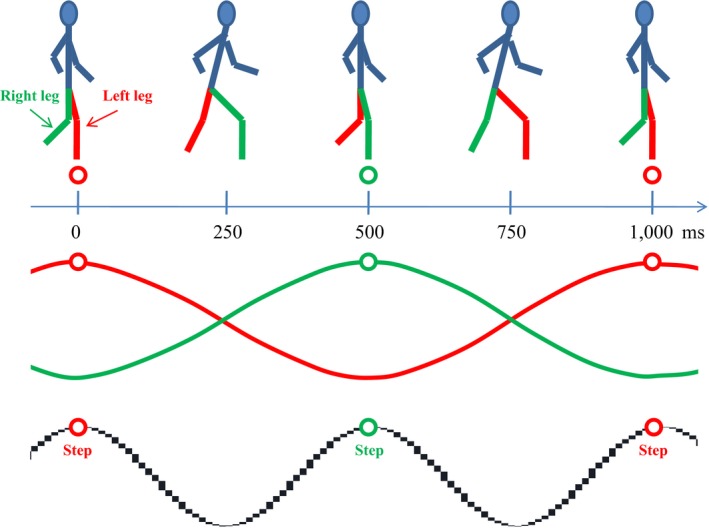
Bipedal locomotion is another example of binary‐coupled oscillators. Leg movement during walking can be described in terms of two oscillators that oscillate (roughly) in counterphase. When full body weight is on one leg (see e.g., the red circle at the peak of the red sinus wave), the other leg does not carry body weight (see the corresponding trough in the green sinus wave). The red and green sinus waves describe stride frequency (f) which is defined by the leg angle time series. Step frequency is characterized by the black sinus wave. Stride frequency for slow to medium walking speed is around 0.9 Hz. In the example above, stride frequency is 1 Hz, and step frequency is 2 Hz.

The movement of the two legs during walking is the most prominent example of body part oscillations. It can be described in terms of coupled oscillators (cf. Strogatz & Stewart, 1993) as is illustrated in Fig. 7. Each leg ‘swings’ in a similar way as a pendulum does and its preferred frequency is close to its resonance frequency of about 0.85 Hz. The resonant frequencies of arms and legs are similar, allowing them to oscillate at the same frequency. It is an interesting observation that at self selected (but also faster) walking speeds (of about 1.5 m/s and beyond) arms and legs oscillate at the same frequency and in phase within each body side, but in counterphase relative to the opposite body side (e.g., Murray *et al*., 1967). Or in other words, ipsilateral limbs swing in phase but are in counterphase to their contralateral limbs (as is described in Fig. 7 for leg movements). Thus, the movement of arms and legs can be described as two pairs of coupled oscillators, operating in synchrony with a 1 : 1 frequency ratio.

At slower walking speeds (of about 0.75 Hz and below) the frequency ratio changes from 1 : 1 to 2 : 1 in a way that arms swing twice as fast than legs. In addition, the out of phase arm movements disappear and change to an (roughly) in phase movement (e.g., Wagenaar & van Emmerik, 2000; Kubo *et al*., 2004). For other body movements, such as thoracic and pelvic rotations, similar phase transitions have been observed (Wagenaar & Beek, 1992). Most interestingly, when the arm to leg frequency ratios are analyzed individually for each subject during the transition from slow to self paced (or fast) walking speed, the observed frequency ratios were always either 1 : 1 or 2 : 1 (depending on walking speed) with minor deviations of about 0.1 Hz only (Donker *et al*. 2001).

## Discussion

The binary hierarchy brain body oscillation theory provides an algorithm that describes the relationship between brain and prominent body oscillations such as HR, BF, and LFF quite faithfully. But there are some open questions which should be addressed. One refers to the possibility that there are different frequency hierarchies, which emerge in a task‐ and state‐dependent manner. Another question refers to the central role of HR as a scaling factor. Yet another question is the communication with the environment. If the binary hierarchy is important for the communication within and between brain and body oscillations, one might speculate that it also may play a role for the communication with the environment. Most interestingly, as will be shown below, musical sounds are also organized along a binary hierarchy.

### Are there different hierarchies?

It is assumed that the binary hierarchy theory describes the frequency architecture of the conscious human brain (Klimesch, 2013), in which alpha is the dominant oscillation. As already mentioned, alpha may play an important role for a ‘default mode processing state’, which can be described as the ability to be consciously oriented in time, space, and context (Klimesch, 2012). This view is quite similar to the assumed function of the DMN (Raichle *et al*., 2001). Because cognitive, emotional, and motor processing requirements vary widely, it might well be the case that there are different frequency hierarchies, with center frequencies of different systems, such as sensory‐motor or emotional systems. Optimal frequency separation may be another reason for assuming a hierarchy of center frequencies fdg(i) that are separated by the golden mean (e.g., Pletzer *et al*., 2010). This idea is expressed by formula (2a):

(2a) fdg(i)=1.25∗giHzg=golden mean

Formula (2a) may play an important role particularly for slower frequencies, because frequencies of the binary hierarchy (e.g., 2.5, 5, and 10 Hz, for i = 1, 2, 3) and frequencies of the golden mean hierarchy (e.g., 2.02, 3.27, and 5.30, for i = 1, 2, 3) are closely interleaved. This means that small frequency shifts determine optimal coupling or decoupling. As an example, if delta shifts down to 2.02 Hz and theta up to 5.30, both oscillations are optimally (frequency) decoupled.

It should also be noted that for the rat brain a different oscillatory hierarchy, which is based on Euler's number (e = 2.718……) was suggested by Buzsaki and colleagues (Penttonen & Buzsaki, 2003; and Buzsáki & Draguhn, 2004). According to these authors, EEG oscillations form a linear hierarchy on a natural logarithmic scale. This hierarchy can be expressed by the following formula (3):

(3) $$\text{fde(i)}\;=\;e^{i}\;\text{Hz}$$

Delta as the first frequency in the hierarchy (with i = 1) has a mean frequency of 2.718 Hz which is defined by fde(1) = e^1^ = 2.718 Hz. The frequencies for theta, beta, and gamma (represented by fde(2), fde(3) and fde(4)) are equal to e^2^, e^3^, and e^4^ with 7.388, 20.079, and 54.576 Hz respectively.

The predictions of the binary and Euler's hierarchy are not compatible for at least two reasons. Formula (3) gives no appropriate estimate for (human) alpha frequency and it lacks a scaling factor which means that all frequencies are identical for all individuals across all species.

It already has been emphasized that two distinct body oscillations, traditional Mayer waves with a frequency of about 0.1 Hz, and gastric waves with a frequency of about 0.05 Hz fall outside the frequency bands that are predicted by the binary hierarchy. They are not frequency aligned to the oscillations that establish the binary hierarchy. Whether Mayer waves and gastric waves are part of a different hierarchy remains an open question.

#### The frequency architecture in sleep

Sleep EEG is a good example for state‐dependent frequency changes. Alpha slows down and disappears as dominant oscillation before the onset of sleep. After sleep onset (except REM sleep), two brain oscillations are dominant, slow oscillations (SO) of about 0.75 or 0.8 Hz (e.g., Diekelmann & Born, 2010; Staresina *et al*., 2015), and spindle oscillations with a mean spindle frequency (SF) of about 13 Hz (for a review see e.g., De Gennaro & Ferrara, 2003). With the exception of SF, the general picture is that of a general slowing of frequencies, which is also apparent in body oscillations (e.g., HR drops from about 75 bpm to about 60 bpm = 1 Hz). None of the frequency ratios between these oscillations (neither SF/HR ~ 13, nor SF/SO ~ 17, or HR/SO ~ 1.3) represent binary multiples, they rather suggest phase de‐coupling between frequencies. Amplitude coupling, however, plays an important role during sleep, as Staresina *et al*. (2015) have shown for human subjects. They found amplitude coupling between SO, SF, and ripples in hippocampus, which presumably is associated with memory consolidation.

### The implications of HR as scaling factor

Cross‐species studies in mammals have found a relationship between HR and body size (cf. Noujaim *et al*., 2004) that can approximately be described by the following formula (4):

(4) $$\text{HR}\;=\;235*{\text{BM}}^{-0.25}\;\;\;\text{BM}\;=\;\text{Body mass in kg}$$

Formula (4) states that HR decreases with increasing body size (as measured by BM). As an example, an elephant with a BM ≈ 3400 kg has a predicted and measured HR of 31 and 35 bpm respectively, whereas a mouse with a BM ≈ 0.027 kg has a predicted and measured HR of 579 and 723 bpm respectively (Human: BM ≈ 66 kg, predicted and measured HR = 82 and 75 respectively).

Whether this scaling property also applies for humans of different body size and body mass (BM) is an open question, because normative data are not (readily?) available for analyses. Indirect supporting evidence comes from the decline in HR from young children (babies have a HR of about 120 bpm; cf. Fleming *et al*., 2011; web appendix) to adulthood, and from studies on total body blood volume (which is correlated with body size), showing a negative correlation with HR (Lehrer, 2013).

In their review, Buzsaki *et al*. (2013), show convincingly that brain oscillations are quite stable between different mammalian species with different body sizes (such as mice, humans, or elephants). Is this evidence against the presented theory which states a direct relationship between brain oscillations and body size? Surprisingly, the answer is no – as the examples listed in Table 1a show – because of an interesting property of formula (2): If the numerical relationship between different scaling factors s also exhibits a doubling/halving ratio, then the predicted frequencies are identical. The examples shown in Table 1(a) were selected in this way. HR for a mouse is eight times faster than for humans, whereas HR of humans is four times faster than for elephants. The red ovals mark frequencies which are typical for human delta, theta, alpha, and beta. But these same frequencies are also predicted for mice and elephants despite their enormous differences in body size. The important point here is that according to formula (2), each frequency carries a ‘name’ (the numerical value of i) which is different for each species and which defines its distance from fd(0), which represents HR. This means that rhythms with the same center frequencies will have different functional meanings. As an example a 10 Hz rhythm for humans equals fd(3) which reflects alpha, but a 10 Hz rhythm for mice equals fd(0) which reflects HR. This does not mean that there is no brain oscillation in mice with 10 Hz, it means that a brain oscillation with 10 Hz exhibits a 1 : 1 frequency ratio with HR. What is different, is the ‘dimensional’ distance, the distances of fd(i)) relative to HR (fd(0)). The possible implication is that the coupling between brain and body oscillations (such as HR and breathing) is different between species.

If the numerical examples for HR of different (theoretical) species ‘a’ and ‘b’ are chosen in a way that they exhibit the largest possible deviation of a doubling halving relationship in HR relative to humans, then the predicted frequencies will not be identical between species, as is illustrated in Table 1b. In this example the values for HR are shifted exactly halfway between the next higher/lower frequency (e.g., for species ‘a’ HR equals the mean between the human fd(−1) and fd(0)), which is the largest possible deviation. Even in this case both species have a 7.5 Hz rhythm, which is very close to the lower frequency limit of the human alpha. Nonetheless, even for these species with the largest possible numerical distance from the human oscillatory hierarchy, frequencies exist that are close to human alpha (or theta, beta, etc.).This means that on one hand, frequencies remain comparatively consistent between species, as emphasized by Buzsaki *et al*. (2013), but on the other hand also, that significant deviations between (and most likely also within) species exist.

### Music and the binary hierarchy

Musical sound is organized in ‘modules’ (octaves) comprising 12 tones with increasing frequency and a fixed frequency ratio (r) between neighboring tones. The pitch of the first tone of the next higher octave has twice the frequency of that of the first tone of the (neighboring) lower octave. Or, in other words, the pitch of the 13th tone is twice as high as the 1st tone. Because of the fixed frequency ratio between the 12 tones, and the doubling/halving relationship between the 1st and 13th tone, the numerical value of r equals the 12th root of 2. This relationship allows one to determine the frequency of all tones of the pitch scale, if a ‘reference’ or ‘basic’ tone is defined. Usually the ‘Kammer A’ (which is the 50th tone in the scale) is the reference tone with a frequency of exact 440 Hz. Accordingly, the 1st tone in the scale has a frequency of 27.5 Hz. The frequencies of all tones t(i) of the pitch scale can be defined by the following formula (5):

(5) $$\text{t(i)}\;=\;s*{\left(12\text{th root}\left(2\right)\right)}^{i}\;\text{Hz}\;\;\;s\;=\;27.5\;\text{Hz}\;\;\;i\;=\;1,2,\ldots \ldots$$

The interesting fact here is that the structure of formula (5) is identical with formula (1), which describes the relationship between brain body oscillations. Both formulas comprise a scaling factor and a scale‐free doubling/halving power law. The only difference is that for music, intervals between the doubling/halving frequency domains are introduced.

The similarity in the frequency architecture may well be the reason that sound plays a vital role for inducing emotional feelings and emotional communication via music. Choir singing is a good example. Psychophysiological studies on singing show that song structure, breathing and HRV are coupled (Vickhoff *et al*., 2013). The song structure determines the time windows for breathing (i.e., for inhaling and exhaling) and, as a consequence, drives HRV. Thus, the hearts of singers become synchronized, they accelerate and decelerate at the same time. In addition, in a completely analogous way as speech envelope coupling, music envelope coupling (e.g., Hennig, 2014; Meltzer *et al*., 2015) operates to synchronize brain oscillatory activity between music listeners. Thus, all of these factors, the frequency architecture of musical sound, the coupling with HRV and entrainment of brain oscillations to the structure of music operate to synchronize brain and body oscillations particularly for performing musicians.

## Conclusions

There are three basic conclusions, (i) brain and body oscillations form a single hierarchy, (ii) they are individually scaled, and (iii) they follow a mathematical/physical law. Thus, brain and body oscillations are aligned to each other, their frequencies do not vary randomly or arbitrarily.

Representing a single hierarchy, brain and body oscillations show identical coupling principles. This also holds true for principles that govern coupling between brain and body oscillations. The most ubiquitous principle is phase amplitude coupling, which is the best documented coupling principle not only for brain oscillations but also for body oscillations. It is important to note that HRV – a well investigated phenomenon – has not yet been recognized (or described) as an example of amplitude coupling. But the rhythmic fluctuations in HRV, most strongly influenced from the respiratory system, represent a clear case of amplitude coupling, where the phase of a slower frequency (e.g., of BF) modulates the instantaneous period of HR. The binary hierarchy theory (formula (2)) represents a strict definition of a single hierarchy to which brain and body oscillations belong. This means that if a single frequency is known (such as e.g., HR or alpha), all other frequencies are also known. The same conclusion can be drawn for musical sounds. If a single frequency of a musical sound is known (determined) then all others are ‘known’ (via formula (5)). This property allows the prediction (knowledge) of all frequencies of the oscillatory hierarchy.

The brains and bodies of different individuals are different. As an example, resonance properties of limbs (which depend on their size), the hydrodynamics of the cardiovascular system (which depend on a variety of different factors, such as total blood volume and body mass) and of the brain (such as network size, and extent of myelination) are factors that ‘scale’ the frequencies. This means that each individual has its own frequency structure that depends on a variety of biological and neurophysiological properties.

Probably the most thrilling conclusion is that brain and body oscillations obey (at least in part) mathematical and physical rules. This means that our body functions – including thinking – not only depend on biological ‘laws’, they also depend on laws that are rooted in mathematics/physics.

Biological systems exhibit large fluctuations. They are considered to provide a ‘noisy environment’. Is this evidence against the above described conclusions? Probably not, because frequencies constantly shift. Variations in instantaneous frequency (see Section Frequency jitter and the 1/f shape of the spectrum) or HRV are good examples. But this variability does not reflect noise, it reflects coupling principles between frequencies, which can be understood to reflect communication between brain and body oscillations. The oscillatory hierarchy may be considered a ‘default’ frequency architecture, to which frequencies preferentially shift in a task‐dependent manner.

## Conflict of interest

The author declares no conflict of interest, financial of otherwise.
