# Supplementary material for: The frequency architecture of brain and brain body oscillations: an analysis
Source: Eur J Neurosci. 2018 Oct 24;48(7):2431–53. doi: 10.1111/ejn.14192 (PMC6668003; doi:10.1111/ejn.14192)
Supplement: Supplementary file 1 [file EJN-48-2431-s001.docx]

The Frequency Architecture of Brain and Brain-Body Oscillations: An Analysis

Klimesch, Wolfgang.

_____________________________________________________________________________________

| Review timeline: | Submission date: 05/06/17  Editorial Decision: Reject + resubmit  Revision Received: 03/04/18  Editorial Decision: Major revision  Revision Received: 19/08/18  Accepted: 13/09/18 |  |
| --- | --- | --- |
|  |  |  |

Editor: John Foxe

Reviewer 1: Adam Snyder

Reviewer 2: Jonathan Cannon

Reviewer 3: Nicholas Cooper

Reviewer 4: Benjamin Pittman-Polletta

_____________________________________________________________________________________

1st Editorial Decision	25/07/17

_____________________________________________________________________________________

Brain-Body Oscillations: The Binary Hierarchy Oscillation Theory

Dear Prof. Klimesch,

Your manuscript was reviewed by three external reviewers as well as by the editors.  Based on these reviews, we regret to inform you that we are not able to accept your manuscript for publication in EJN in its present form, and we could only consider a resubmission of the paper if all of the very critical points raised by our reviewers can be fully addressed. For example, both reviewer #1 and reviewer #2 raise a legitimate and critical question about the falsifiability of the theory you present, and indeed, some of your own important empirical work would appear to violate the basic model you forward.  Questions were also raised as to why heart rate is picked as the fundamental body oscillation. What is the basis for this claim?  The manuscript is in need of a substantially more critical look at the central tenets of the proposed theory – are there other potential accounts, is there evidence that might contravene any of the central claims, and if not, what would constitute a strong test of the theory?  There should also be some treatment/acknowledgment of the implications of the inverse model for what it is that one can realistically say about the contributions of potentially overlapping sources of activity on the sorts of oscillatory dynamics we can record at the scalp surface.  There are calls for consideration of additional papers in the literature, and please clarify whether your figures actually represent measured responses or are purely illustrations.

Obviously, a resubmission of your manuscript could only be considered on the basis of extensive revisions, requiring the description of new evidence and/or additional analyses of your data. When you prepare your re-submission, please consult the Author's Checklist provided at the bottom on this email, notably with regard to characterization of reagents and reporting of statistical analyzes in the Results section and Figure legends.

When revising the manuscript, please embolden or underline major changes to the text so they are easily identifiable and DO NOT leave 'track change' formatting marks in your paper. If the changes made are extensive, please also provide an unmarked version.

Please provide a detailed response to the reviewers' comments, describing the changes you have made in the text. Please upload this response letter as a separate Word (.doc) file using the file designation "Authors' Response to Reviewers" when uploading your manuscript files.

When finalized, please upload your complete revised manuscript onto the website as a Word (.doc or .docx), or .rtf file.  Please also ensure that a complete set of tables and figures is included as separate files, even if these have not changed from the originals.  At this stage it is necessary to provide high resolution figures.  Please see important instructions below.

To re-submit your manuscript, please go to your Author Centre and check in 'Manuscripts with decisions' under the previous manuscript number for a 'Create a resubmission' link.  Also, please note that only the Author who submitted the original version of the manuscript should submit a revised version.

Thank you for submitting your work to EJN.

Kind regards,

John Foxe & Paul Bolam
co-Editors in Chief, EJN


Reviews:

Reviewer: 1

Comments to the Author
The author's theory is that oscillatory physiological processes are inter-related in such a way that makes their frequencies distributed logarithmically with base 2. It's an interesting theory, but I have two main concerns. The first is that it isn't clear to me how to falsify this theory. For example, in the manuscript there are several instances where the author presents as evidence that the frequency of one process is "close to" what would be predicted by doubling or halving the frequency of another, presumably related process. How "far" would a frequency have to be from the predicted value to invalidate the theory? My second main concern is that I'm unclear on the details of exactly how this theory is supposed to play out in an individual. If my heart rate goes up, does my alpha center frequency also go up? Why do brain oscillations have bandwidth? There isn't really any room in this theory for bandwidth that I can see. The author makes an effort to address this in terms of "frequency jitter", but what about the case of neural cell assemblies that have a stable oscillation (i.e., not "jittering" over time) at slightly different frequencies? For example, the author himself has previously reported that different brain regions have different alpha center frequencies (Klimesch et al., 1993, Brain Topography, vol. 5). How does the binary hierarchy theory account for that? I feel some discussion of these matters would improve the manuscript.

1) On p. 8, the author writes, “The binary hierarchy…. represents a universal scale free power law.” The term, “scale free” implies that no one scale is more important than another. Yet the author argues at other points that heart rate is “the most [important] body oscillation” (p. 3). Why should this oscillation, as opposed to e.g. the Mayer waves, or respiration, or alpha, etc., be defined as the “fundamental frequency”? I think the author’s point can be made to the same extent just by noting the apparent harmony without ascribing a special role to heart rate. If there is some ecological reason why the heart rate is special, I don't find the reasoning to be well-explained in the manuscript.

2) On p. 2, the author states “it will be argued that there are (physiologically) only two coupling principles, (i) amplitude (envelope) coupling… and (ii) phase coupling…”. However, I do not find evidence arguing for this claim to follow. The manuscript does include examples of the two types of coupling provided, but does not describe the types of coupling that are *excluded* by the claim (e.g., amplitude-amplitude correlations  --am I correct that this is the only remaining possible combination? That is, besides phase-phase, phase-amplitude, and amplitude-amplitude, what other sort of coupling could be possible?) nor does it include evidence that these other potential types of coupling do not occur.

3) More generally, I find much of the evidence presented in the manuscript to be confirmatory. That is, the evidence is mostly a sequence of observations that confirm the theory (rhythm X is about half the frequency of rhythm Y which is itself about half the frequency of rhythm Z, etc.), which of course could be selected for that purpose. Without an experimental manipulation that aims to explicitly falsify the theory, I am left wondering what passive observations would have been like that could have disproved it. For example, what would my heart rate and alpha frequency need to be for us to conclude that the binary hierarchy can’t be correct? There should be some discussion of the falsifiability of this theory.

4) All of the evidence given relies on average values for a species, but I believe the author’s hypothesis should make strong predictions about the spacing of brain and body oscillation frequencies within individuals, correct? Has anyone looked at that? It seems like it should be relatively straightforward to get to the bottom of this…

5) I am having difficulty following the reasoning at the bottom of page 2: “The argument here is that in all examples, phase is the driving force for coupling. The central conclusion from these findings is that phase can establish its impact… only, if its frequency exhibits a comparatively small jitter. In a broad band the phases of different oscillations with different frequencies will tend to cancel each other. This is taken as strong evidence for the existence of more or less distinct center frequencies.” I believe the argument is that because theory requires the phases to be stable, that that serves as evidence for what’s going on in nature. This strikes me as the reverse of the usual tack, for which scientists take observations of what goes on in nature as evidence for or against theory. In fact, when we measure the power spectrum of brain activity we usually do not see narrow-band peaks, especially e.g. in the gamma band. Does the author have experimental observations to bolster this argument?

6) Another good example of the behavioral correlates of cross-frequency phase-amplitude coupling that the author might consider reviewing was provided by Fiebelkorn, et al. (2013), NeuroImage, vol. 69.

7) P. 7: “Alpha peaks appear usually during…. Frontal midline theta… can be observed during…. Sleep spindles are associated with…” These claims need citations. For the alpha claim, one source the author might consider is a review from Foxe and Snyder (2011), Frontiers in Psychology, vol. 2.  In the following paragraph, a citation is needed for the claim that “beta exhibits some relation to motor performance”.

8) On p. 11, the author writes, “This critical issue is closely linked to two questions, to the number of different frequency hierarchies (see above) and to the physiological meaning of frequency jitter which can be described by cycle to cycle fluctuations in (instantaneous) amplitude and (instantaneous) period.” The author then goes on to describe some potential “explanations” of frequency jitter. One factor that was not discussed, however, is that our recording electrodes are integrating over relatively large areas of space, and may be picking up signals from multiple channels of neural communication that may be operating on slightly different frequencies (i.e., not so much cycle-to-cycle change in the duty cycle of a single source, but the moment-to-moment changes in amplitude of multiple, superimposed sources.

9) Following-up on the previous issue, at several points in the manuscript it seems as though the author is suggesting that the “scaling factor” for formula 1 is universal within an individual. How critical is this for the author’s central premise? I could imagine that some neural cell assemblies might be coupled more or less strongly to others and therefore could have some degree of freedom with respect to the exact value of the scaling factor at any given time. For an oversimplified example, maybe the left hemisphere resonates at 10.1 Hz whereas the right hemisphere prefers 12.3 Hz (see also, Groppe et al., 2013, NeuroImage, vol. 79, who used ECoG to find “substantial variability in the dominant frequencies cortical regions…”; and of course, see also Klimesch et al., 1993, Brain Topography, vol. 5). Could frequency jitter actually not be a problem, but rather a genuine use of frequency sub-bands? Would this perspective undermine the whole “binary hierarchy” framework? What is the role of bandwidth in this theory?

10) How salient of a force does the author suppose the “binary hierarchy” to be? Is this more of a developmental principle that stakes out where on the spectrum each body function will roughly fall, but then from moment-to-moment their frequencies are somewhat independent? In other words, does the author predict that my individual alpha frequency should be about twice as fast if I measure it while I’m on a treadmill with a heart rate of 144 bpm as opposed to when I’m sitting quietly at 72 bpm? This seems unlikely. While I'm not personally well-versed on this issue, I know there is a small, but growing, literature on EEG changes during exercise; For example, Gutmann et al., 2015, Neural Plasticity, vol. 2015, measured individual alpha peak frequency before and immediately after exercise, and they did report an increase (in only one of two exercise conditions), but the size of that effect was only a quarter of a Hertz, not nearly what would be predicted by a binary hierarchy based on heart rate. What's more: the peak frequency remained elevated after ten minutes of rest, presumably after heart rate would have decreased to near resting levels, which also doesn't seem to fit with the binary hierarchy theory.

11) It is not clear which of the figures depict measured data, and which are merely cartoon examples. My impression is that only figure 6 depicts measured data and the rest are all just illustrations. Is that correct? This should be made explicit in the figure legends.


Reviewer: 2

Comments to the Author
Dr. Klimesch,

I have great respect and appreciation for your experimental work and your excellent reviews on brain oscillations. However, I cannot recommend this manuscript for publication.

The idea that 2:1 locking is a general principle uniting brain and body oscillations is an interesting one, and may deserve some type of attention in the literature. Some of my criticisms of this work could be addressed in a simple revision. However, I believe that there is a systemic flaw that may be very difficult to address without a deep reconsideration of the subject at hand: namely, that the theory proposed in the work is broad enough to be practically unfalsifiable. Throughout the manuscript, evidence is cherry-picked from experimental literature, and all evidence that seems to contradict the theory is dismissed in ways that could be used to dismiss practically any contradictory evidence that could be raised. Nowhere is mention given of what observations could show the binary oscillation theory to be false. I will address this problem in more detail below.

The theory presented in this work is made up of several parts. First, that the important biological frequencies obey a geometric scaling law; second, that this law scales frequencies by powers of two; third, that the purpose of this property is to facilitate communication between processes with different characteristic frequencies via nesting; and fourth, that the hierarchy of oscillations is scaled to match the organism’s heart rate. The issue of “m:n phase-amplitude coupling” is raised, but it is not clear what purpose is served by this discussion — phase-amplitude coupling can clearly occur even if the frequencies do not exist in an integer m:n relationship, so this seems largely irrelevant to this theory of integer-nesting oscillations.

The first part, the geometric scaling law, follows from the nature of oscillations -- if the essential frequencies are indeed systematically related to each other, it is to be expected that they would form a geometric progression. If any m:n relationship of oscillation periods is to be meaningful in itself, this relationship can only be preserved if frequencies are shifted together multiplicatively. This is well-established in music, as is noted in the manuscript, and in any other study of interacting oscillations. In the manuscript, this idea is presented at length, but this very basic concept and the accompanying math (including the repeated explanations of the relationship between period and frequency) do not deserve more than a brief acknowledgement of an assumed shared understanding.

The second part, the scaling factor of two, is contradicted by your own work from 2010 discussed in this manuscript, in which you argue that the biophysically relevant brain oscillation frequencies exist in a golden-mean-based hierarchy. The only reason that both of these theories can be argued is that there are so many types of oscillation in the brain (none of the greek-letter oscillations can be said to represent a single monolithic phenomenon), and even oscillations created by the same process are often frequency-flexible. In this paper, you point out that there are distinct upper and lower alpha bands, but the inconsistency of this dichotomy with the theory is ignored. By focusing on some oscillatory phenomena instead of others that contradict the theory (e.g., the 0.1Hz oscillation referenced repeatedly in the paper) and using sufficiently large frequency bands, any scaling factor for essential brain frequencies can be argued. No principled method is offered for identifying “center frequencies,” and the apparent lack of center frequencies in brain oscillations is dismissed by claiming that each type of oscillation is frequency-flexible enough to disguise its center frequency. This allowance is one factor that makes the theory so difficult to falsify.

The third part, that power-of-two nesting facilitates communication, is not argued in this paper at all. “Communication” is never even given a concrete definition, nor is the mechanism by which period-nesting could improve communication discussed.

The fourth part, the heart-rate basis of the hierarchy, is rendered completely unfalsifiable by the cross-species comparison: the vastly different heart rates of different species seems to be deeply inconsistent with a system in which all brain oscillation frequencies scale with heart rate, but this problem is dismissed by claiming that the same frequency in two different species could represent different powers of two. This would imply that 10Hz oscillations in a mouse would interact with the body in a fundamentally different way than in humans; yet experiment shows that 10Hz oscillations appear in the same contexts in mice and humans and appear to serve the same function. If this is not counter-evidence for a hierarchy that scales with heart rate, what else possibly could be? Further table 1b shows that the trick of assigning the same rhythm to different powers of two could shoehorn any species with a binary-like hierarchy of frequencies into a heart-rate-scaled hierarchy, making the theory of heart-rate scaling impossible to prove or disprove.

I can imagine several types of data that might support parts of the theory. There must be data somewhere that records characteristic human alpha frequency and body size — to make a convincing argument for this theory, it would help to find and consider data of this type. I would also be interested to see evidence that any two brain oscillations systematically vary in frequency together in a single individual, or any evidence of brain oscillations phase locking 2:1. Neither seems to be present in this paper. I am aware of a certain case of 2:1 beta/alpha locking (Steph Jones), but this seems to be a consequence of an alpha period interrupted repeatedly at its peak and thus subdivided rather than an episode of two independent oscillations being coordinated.

The evidence of nesting in body oscillations is somewhat convincing in the argument that systematic phase locking does occur. However, even here I don’t find the binary phase locking theory very strong. At one point, a 1:10 relationship is cited as evidence of nested body oscillations. For one thing, any two frequencies anywhere near a 1:10 relationship could be claimed to have an integer relationship of 1:9, 1:10, or 1:11 by rounding up or down. For another thing, the fact that 10 is not a power of two is handily dismissed. In the case of breathing and walking, 1:2 nesting follows from the existence of the dichotomy of full vs. empty lungs and the dichotomy of left vs. right, respectively. The 1:1 coupling mentioned near the end of the manuscript is completely trivial — any two things that occur simultaneously with any periodicity are 1:1 coupled.

The binary hierarchy theory is sufficiently vague that the main significance articulated in the Discussion is effectively just that it would be very exciting to find a mathematical law governing the brain and body.

The seven figures make mountains out of molehills. For instance, figure 4 uses a strangely scaled y-axis to make a pretty picture out of what should simply be displayed as a line on a log scale, while figure 7 takes half a page to show that the left foot hits the ground on every second step.

It is valuable to note that the ERP likely represents transient phase alignment. However, since this alignment is transient, it must represent the alignment of oscillations with no sustained systematic period-nesting.

All in all, I believe that this manuscript falls prey to the cognitive bias of finding elegant dichotomies everywhere. Patterns can be picked out in any sufficiently complex data and theories can be created from them, but without carefully establishing the bounds of the theory and the conditions under which it could be shown false, this paper does not constitute a clearly articulated idea, and risks undermining the credibility of more substantive theories in the field of oscillations.

I would ordinarily be willing to read the paper again on a resubmission, but I am only available for reviews for a short period of the summer, after which daily responsibilities would not allow me time.

Reviewer: 3
**Review of EJN-2017-06-24636(NOSC)**

I would like to preface this review by stating that I have not reviewed a purely

theoretical paper such as this before and am therefore a little unsure as to my

suitability for the task. Having said that, I will proceed with my comments as I

think this is an important and excellent paper that warrants publication.

This paper is very clearly written and technically excellent, as one would expect

from an author who has helped to define the study of brain oscillations for the

past few decades. I think it fits well within the scopes of EJN and there is no

issue with Authors’ Guidelines. I do however have a few comments and

suggestions that I feel could help clarify certain aspects for the reader and also

possibly extend the theory further (into a different corporeal domain). I will list

these now in the order that they appear in the manuscript (not in order of

importance; points 6 & 8 are my main points):

1. Pg.2 (abstract) – perhaps the first couple of sentences could make more

explicit that this paper will argue that the principles already hypothesised

in brain oscillations will be extended to other non-brain oscillations? (it

would be nice to have a slightly better link from brain to brain-body).

2. Pg.2 2nd line of intro – suggest inserting “cortical oscillatory” between

“many” and “frequencies”.

3. Pg.3 3rd para – the notion of a ‘binary hierarchy’ could do with unpacking,

to make it clearer to the reader at this point.

4. Pg4 7 lines from bottom) – “without the influence of theta” – it would be

useful for the reader to be told what the possible consequences of this

would be.

5. Pg7, 3rd para – I’m a little worried (especially given the author’s extensive

work in this field) that the brief descriptions of the functional significance

of the main EEG bandwidths come across as a little simplistic and may

encourage somewhat lazy interpretation of data sets from less

experienced EEG practitioners. Having said that, it is not for this paper to

answer all such issues, I just wanted to bring it to the author’s attention.

6. Pg13 section 4 (and elsewhere) – the author may also like to consider

extending the scope of his theory to incorporate the electrogastrogram

(EGG)? There is increasing interest in the link between the CNS and the

enteric nervous system and one notable recent publication described a

1:1 relationship between the phase of the slow gastric rhythm and the

amplitude of the cortical alpha rhythm (Richter et al, 2016, NeuroImage).

Given that the frequency of normal gastric slow waves is about 3 cycles

per minute (Yin & Chen, 2013, JNM), by my rough calculations, that puts it

somewhere between BOLD IMF 1 & BOLD IMF 2 in figure 4.

7. Page 16, 3rd para – for me, the section about the ‘length of the R-R

interval’ could be made a little clearer (given the not obvious use of the

term ‘amplitude’ to describe it).

8. Page 16, para 4, final sentence – arguably this change in HR also varies

according to the locus of attention (i.e. internal vs external). Perhaps a

brief inclusion of the Lacey’s theories (e.g. 1967, 1970) here would be

helpful (and may also tie in the link between baroreceptor range and

alpha activity – e.g. Wolk & Velden, 1987)?

9. Pg18, 1st para – DMN – is it worth also making clar here that DMN is

considered an internally focused state (which would fit well with DMN

being associated with upper alpha) and would link well to the 1st para on

page 20?

Spellings and typos

 Pg9, 2nd line of 3.1 – “possibly” should read “possible”

 Pg10 line 2 – “fac,t” should read “fact”

 Pg10 3rd para of 3.2 – extra unnecessary “a” between “for” and “an”

 Pg22, line 11 – “und” should read “under”

 Pg24, 3rd from last line – superfluous “in”

 Pg25, last line – perhaps “and” would be better than “/” here?

____________________________________________________________________________________

Authors’ Response	03/04/18

_____________________________________________________________________________________

Response to Reviewer 1

Dear Reviewer, many thanks for your detailed and thoughtful comments. Here, I give an overview about how I have dealt with them. I have numbered your comments in 3 main concerns and 11 specifc comments, and discribe, how and where I have made changes in the revision.

Before I go into the discussion with your comments, I would like to mention that I have changed the revised ms in order to clarify which issues lead to the foundation of the suggested theory. I have done this in response to the other two Reviewers. I have changed the title (to: The Frequency Architecture of Brain and Brain-Body Oscillations: An Analysis) and reorganized the ms in an attempt to focus on three issues, on (i) coupling principles in brain and (ii) body oscillations and (iii) to present my theory as a result of the analyses of (i), which makes predictions on (ii). All Sections are revised and reorganized accordingly. The line of argumentation is summarized in Section 1.

Main concerns:

1. It is not clear, how to falsify the theory. How "far" would a frequency have to be from the predicted value to invalidate the theory?

*This issue is addressed in Section 4.1 that deals with the predictions of the theory (see page 15 and followng). In short, the theory is falsified if the frequency of a reported oscillation falls outside the respective frequency band that is predicted by the theory.*

2. I'm unclear on the details of exactly how this theory is supposed to play out in an individual. If my heart rate goes up, does my alpha center frequency also go up?

*This issue is addressed in section 4.1.4. about the covariation of brain and body oscillation (page 20 and following). In brief: Yes, frequencies are predicted to covary but not in an 1:1 manner. They may jump e.g., to an 1:2 ratio (as is the case for breathing, see page 16) or to an irrational ratio reflecting decoupling.*

3. Why do brain oscillations have bandwidth? There isn't really any room in this theory for bandwidth that I can see.

*Sorry for this misunderstanding, I have put much effort in clarifying this crucial issue. Sections 1-3 are revised accordingly. The basic arguments (summarized in section 1) are (i) that not all frequecies are equal,(ii) that center frequencies of traditional EEG bands reflect ‚preferred‘ frequencies that are associated with specific processing domains (see section 2). (iii) Most importantly, the analysis of cross frequency coupling (section 3) shows that phase is the driving force for coupling. The central argument here is that phase can establish its impact – physiologically as well as mathematically – only, if its frequency is a dominant oscillation (even with large jitter) within a certain frequency band. In a broad band with ‘equal frequencies’ that lacks a distinct oscillation, the phases of different frequencies will tend to cancel each other due to the superposition of this frequencies (as measured by standard EEG/MEG recordings). (iv) Finally and most importantly, center frequencies may shift to couple or decouple with other frequencies (see Sections 3.3.1 and 3.3.2 pag 12 and following).*

Specific Comments

1. The author writes, “The binary hierarchy…. represents a universal scale free power law.” The term, “scale free” implies that no one scale is more important than another. Yet the author argues at other points that heart rate is “the most [important] body oscillation” .

*Yes it’s both: scale free (because of the power function) and scaled because of the introduction of a scaling factor, s (Formula 2). Only necause of the relationship between s and HR, HR may be assumed the most important body oscillation. In the revision I have separated two questions: (i) Is HR part of the binary hierarchy (Section 4.1.1, page 15) and (ii) what are the implications of the HR scaling assumption (Section 6.2. page 32 and following). I have done this, because the HR scaling assumption is not an assumtion underlying the suggested theory, but a possible implication.*

2. The author states “it will be argued that there are (physiologically) only two coupling principles, (i) amplitude (envelope) coupling… and (ii) phase coupling…”. However, I do not find evidence arguing for this claim to follow. The manuscript does include examples of the two types of coupling provided, but does not describe the types of coupling that are *excluded* by the claim (e.g., amplitude-amplitude correlations --am I correct that this is the only remaining possible combination?

*I refer to this issue in Section 3 (page 5), first paragraph.*

3. I am left wondering what passive observations would have been like that could have disproved it. For example, what would my heart rate and alpha frequency need to be for us to conclude that the binary hierarchy can’t be correct?

*The answer is: if the ratio between individual HR and individual alpha frequency (IAF), measured in awake resting subjects, would not be 1:8 (see Section 4.1.1, pages 15,16)*

4. All of the evidence given relies on average values for a species, but I believe the author’s hypothesis should make strong predictions about the spacing of brain and body oscillation frequencies within individuals, correct? Has anyone looked at that?

*The reviewer is right, but there are almost no studies on this issue. Nontheless, I found some recent work which plays an importent role. This e.g., is Siebenhüner et al. (2016), see e.g. Fig. 3b,c and Gutmann et al. (2018), see Sections 3 and Section 4.1.1(pages 15,16)*

5. I am having difficulty following the reasoning at the bottom of page 2: “The argument here is that in all examples, phase is the driving force for coupling. The central conclusion from these findings is that phase can establish its impact… only, if its frequency exhibits a comparatively small jitter. In a broad band the phases of different oscillations with different frequencies will tend to cancel each other. This is taken as strong evidence for the existence of more or less distinct center frequencies.” **I believe the argument is that because theory requires the phases to be stable, that that serves as evidence for what’s going on in nature**. This strikes me as the reverse of the usual tack, for which scientists take observations of what goes on in nature as evidence for or against theory. In fact, when we measure the power spectrum of brain activity we usually do not see narrow-band peaks, especially e.g. in the gamma band. Does the author have experimental observations to bolster this argument?

*The theory does not require stable phases or stable frequencies. Frequency jitter is the essence of coupling and decoupling. Both principles refer to specific frequency ratios. Please see my response to main point 3 above and the line of argumentation in Sections 1-3.*

6. Another good example of the behavioral correlates of cross-frequency phase-amplitude coupling that the author might consider reviewing was provided by Fiebelkorn, et al. (2013), NeuroImage, vol. 69.

*Many thanks for this hint, this work is now included (see page 11).*

7. Alpha peaks appear usually during…. Frontal midline theta… can be observed during…. Sleep spindles are associated with. These claims need citations. For the alpha claim, one source the author might consider is a review from Foxe and Snyder (2011).

*Many thanks, you are right. I have now referred to several studies/reviews, including the suggested work by Foxe and Snyder (2011). See pages 3 and 4.*

8. The author writes, “This critical issue is closely linked to two questions, to the number of different frequency hierarchies (see above) ………The author then goes on to describe some potential “explanations” of frequency jitter. One factor that was not discussed, however, is that our recording electrodes are integrating over relatively large areas of space……

*Many thanks, you are righ againt. This issue is emphasized in Section 3.3.2, second paragraph on page 14.*

9. Following-up on the previous issue, at several points in the manuscript it seems as though the author is suggesting that the “scaling factor” for formula 1 is universal within an individual. How critical is this for the author’s central premise? I could imagine that some neural cell assemblies might be coupled more or less strongly to others and therefore could have some degree of freedom with respect to the exact value of the scaling factor at any given time. For an oversimplified example, maybe the left hemisphere resonates at 10.1 Hz whereas the right hemisphere prefers 12.3 Hz (see also, Groppe et al., 2013, NeuroImage, vol. 79, who used ECoG to find “substantial variability in the dominant frequencies cortical regions…”; and of course, see also Klimesch et al., 1993, Brain Topography, vol. 5). Could frequency jitter actually not be a problem, but rather a genuine use of frequency sub-bands? Would this perspective undermine the whole “binary hierarchy” framework? What is the role of bandwidth in this theory?

*Many thanks, these are important issues. In the revision, I emphasize that the binary hierarchy theory (regarding brain oscillations) applies to ‚preferred frequencies‘, which are termed frequency domains only (see Section 2). With respect to subbands: I refer e.g., to high alpha and high theta in work showing peaks in these subbands in memory tasks (see Section 3 pages 8,9 and Fig. 3c). The point here is that frequenciesmay shift in a task dependen manner to couple (or decouple). When they couple, they stay within the predicted bands, otherwise the fall outside the bands.*

10. How salient of a force does the author suppose the “binary hierarchy” to be? Is this more of a developmental principle that stakes out where on the spectrum each body function will roughly fall, but then from moment-to-moment their frequencies are somewhat independent? In other words, does the author predict that my individual alpha frequency should be about twice as fast if I measure it while I’m on a treadmill with a heart rate of 144 bpm as opposed to when I’m sitting quietly at 72 bpm? This seems unlikely. While I'm not personally well-versed on this issue, I know there is a small, but growing, literature on EEG changes during exercise; For example, Gutmann et al., 2015, Neural Plasticity, vol. 2015, measured individual alpha peak frequency before and immediately after exercise, and they did report an increase (in only one of two exercise conditions), but the size of that effect was only a quarter of a Hertz, not nearly what would be predicted by a binary hierarchy based on heart rate. What's more: the peak frequency remained elevated after ten minutes of rest, presumably after heart rate would have decreased to near resting levels, which also doesn't seem to fit with the binary hierarchy theory.

*Many thanks! There is a very relevant recent publication from Gutmann (Gutmann et al. 2018), which plays an important role in the revised ms. Please see Section 4.1.(pages 15,16), but also Section 4.1.4, page 20.*

11. It is not clear which of the figures depict measured data, and which are merely cartoon examples. My impression is that only figure 6 depicts measured data and the rest are all just illustrations. Is that correct? This should be made explicit in the figure legends.

*Done es suggested*

Response to Reviewer 2

Dear Reviewer man thanks for your critical comments. I have numbered them in three main concerns and 9 specifc comments and discribe, how I respond and where I have made changes in the revison.

Main concerns:

1. I believe that there is a systemic flaw that may be very difficult to address without a deep reconsideration of the subject at hand: namely, that the theory proposed in the work is broad enough to be practically unfalsifiable.

*In the revision, I have included a separate Section (Section 4, see page 15 and following) that deals with the predictions of the theory. In short, the theory can be falsified if its predictions (listed in Section 4) cannot be supported by empirical evidence. This is the case if the frequency of a reported oscillation falls outside the respective frequency band that is predicted by the theory.*

2. All in all, I believe that this manuscript falls prey to the cognitive bias of finding elegant dichotomies everywhere. Patterns can be picked out in any sufficiently complex data and theories can be created from them, but without carefully establishing the bounds of the theory and the conditions under which it could be shown false, this paper does not constitute a clearly articulated idea, and risks undermining the credibility of more substantive theories in the field of oscillations.

*Sorry for this opinion. I probably have been too vague in the original version of the ms. In the revison, I have put much effort in clarifying those issues that lead to the foundation of the suggested theory. I have changed the title (to: The Frequency Architecture of Brain and Brain-Body Oscillations: An Analysis) and reorganized the ms in an attempt to focus on three issues, on (i) coupling principles in brain and (ii) body oscillations and (iii) to present my theory as a result of the analyses of (i), which makes predictions on (ii). All Sections are revised and reorganized accordingly. The basic arguments (summarized in Section 1) motivating my theory are that not all frequecies are equal, that center frequencies of traditional EEG bands reflect ‚preferred‘ frequencies that are associated with specific processing domains (see section 2) and that the theory applies only to these frequency domains. Furthermore, as mentioned above, I have included a separate section about the evalaution of the prediction of the theory.*

Specific concerns:

1. The theory presented in this work is made up of several parts. First, that the important biological frequencies obey a geometric scaling law; second, that this law scales frequencies by powers of two; third, that the purpose of this property is to facilitate communication between processes with different characteristic frequencies via nesting; and fourth, that the hierarchy of oscillations is scaled to match the organism’s heart rate. The issue of “m:n phase-amplitude coupling” is raised, but it is not clear what purpose is served by this discussion — phase-amplitude coupling can clearly occur even if the frequencies do not exist in an integer m:n relationship, so this seems largely irrelevant to this theory of integer-nesting oscillations.

*The argument is that also for amplitude coupling, phase is the driving force. I use this argument to imply the existence of distinct center frequencies: phase can establish its impact – physiologically as well as mathematically – only, if its frequency is a dominant oscillation (even with large jitter) within a certain frequency band. In a broad band with ‘equal frequencies’ that lacks a distinct oscillation, the phases of different frequencies will tend to cancel each other due to the superposition of this frequencies (as measured by standard EEG/MEG recordings). See Sections 1, 2 and 3.*

2. The first part, the geometric scaling law, follows from the nature of oscillations -- if the essential frequencies are indeed systematically related to each other, it is to be expected that they would form a geometric progression. If any m:n relationship of oscillation periods is to be meaningful in itself, this relationship can only be preserved if frequencies are shifted together multiplicatively. This is well-established in music, as is noted in the manuscript, and in any other study of interacting oscillations. In the manuscript, this idea is presented at length, but this very basic concept and the accompanying math (including the repeated explanations of the relationship between period and frequency) do not deserve more than a brief acknowledgement of an assumed shared understanding.

*I completely agree that this issue is a simple mathematical fact. But I don’t see that this fact plays a role in the discussion of coupling principles in the (cognitive) neuroscience community. I think the discussion of the implications of this fact are very important and far from trivial.*

3. The second part, the scaling factor of two, is contradicted by your own work from 2010 discussed in this manuscript, in which you argue that the biophysically relevant brain oscillation frequencies exist in a golden-mean-based hierarchy. The only reason that both of these theories can be argued is that there are so many types of oscillation in the brain (none of the greek-letter oscillations can be said to represent a single monolithic phenomenon), and even oscillations created by the same process are often frequency-flexible. In this paper, you point out that there are distinct upper and lower alpha bands, but the inconsistency of this dichotomy with the theory is ignored. By focusing on some oscillatory phenomena instead of others that contradict the theory (e.g., the 0.1Hz oscillation referenced repeatedly in the paper) and using sufficiently large frequency bands, any scaling factor for essential brain frequencies can be argued. No principled method is offered for identifying “center frequencies,” and the apparent lack of center frequencies in brain oscillations is dismissed by claiming that each type of oscillation is frequency-flexible enough to disguise its center frequency. This allowance is one factor that makes the theory so difficult to falsify.

*No, I do not agree.*

*- With respect to the Pletzer et al. (2010) study: The emphasis here is on the role of the golden mean and on frequency separation, but in my theory the emphasis is on both, optimal synchronization and on frequency separation. In Section 6.1, I argue that there may be more than just one hierarchy principle and explicitely suggest an extension of the Pletzer study.*

*-With respect to subbands: I refer e.g., to high alpha and high theta in work showing peaks in these subbands in memory tasks (see Section 3 pages 8,9 and Fig. 3c). The point here is that frequencies may shift in a task dependen manner to couple (or decouple). When they couple, they stay within the predicted bands, otherwise the fall outside the bands.*

*- With respect to a lack of center frequencies: Please see my arguments regarding the role of phase. Please see my response to specific concern 1. Above.*

4. The third part, that power-of-two nesting facilitates communication, is not argued in this paper at all. “Communication” is never even given a concrete definition, nor is the mechanism by which period-nesting could improve communication discussed.

*In the revison, the Sections about the coupling principles in brain oscillations refer explicitely to the fact that cross frequency coupling is considererd a basic mechanism of neural communication, which – of course - reflects ‚standard agreement‘ in the (cognitive) neuroscience cummunity. The basic argument is that optimal coupling proivides optimal communication. The proposed theory is part of this argumentation.*

5. The fourth part, the heart-rate basis of the hierarchy, is rendered completely unfalsifiable by the cross-species comparison: the vastly different heart rates of different species seems to be deeply inconsistent with a system in which all brain oscillation frequencies scale with heart rate, but this problem is dismissed by claiming that the same frequency in two different species could represent different powers of two. This would imply that 10Hz oscillations in a mouse would interact with the body in a fundamentally different way than in humans; yet experiment shows that 10Hz oscillations appear in the same contexts in mice and humans and appear to serve the same function. If this is not counter-evidence for a hierarchy that scales with heart rate, what else possibly could be? Further table 1b shows that the trick of assigning the same rhythm to different powers of two could shoehorn any species with a binary-like hierarchy of frequencies into a heart-rate-scaled hierarchy, making the theory of heart-rate scaling impossible to prove or disprove.

*I see two separate issues, one referring to the prediction of the theory that HR is a ‚member‘ of the binary hierarchy and one referring to a possible implication of this prediction which is termed ‚HR scaling assumption‘. The latter issue is not an obligatory part of the theory. The reviewer confuses these issues, for which I take responsibility, because the original ms may have been to vague. Now, I have clearly separated these issues (please see Sections 4.1 and 6.2)*

*Sorry, I cannot agree to the opinion of the Reviewer that ‚10Hz oscillations appear in the same contexts in mice and humans and appear to serve the same function‘ If this would be true, hippocampal theta in mice would play the same role as alpha in humans. I can’t see any empirical evidence supporting this view.*

6. I can imagine several types of data that might support parts of the theory. There must be data somewhere that records characteristic human alpha frequency and body size — to make a convincing argument for this theory, it would help to find and consider data of this type. I would also be interested to see evidence that any two brain oscillations systematically vary in frequency together in a single individual, or any evidence of brain oscillations phase locking 2:1. Neither seems to be present in this paper. I am aware of a certain case of 2:1 beta/alpha locking (Steph Jones), but this seems to be a consequence of an alpha period interrupted repeatedly at its peak and thus subdivided rather than an episode of two independent oscillations being coordinated.

*I agree, there should be data, but I could not find any. The problem here is that body size is so much influenced by the proportion of muscles, fat and other factors (such as, e.g. age), which must be considered as control variables.*

*With respect to 2:1 locking, I report several empirical findings in Section 3 of the revised ms.The covariation of frequencies is part of the predictions in Section 4.*

7. The evidence of nesting in body oscillations is somewhat convincing in the argument that systematic phase locking does occur. However, even here I don’t find the binary phase locking theory very strong. At one point, a 1:10 relationship is cited as evidence of nested body oscillations. For one thing, any two frequencies any where near a 1:10 relationship could be claimed to have an integer relationship of 1:9, 1:10, or 1:11 by rounding up or down. For another thing, the fact that 10 is not a power of two is handily dismissed. In the case of breathing and walking, 1:2 nesting follows from the existence of the dichotomy of full vs. empty lungs and the dichotomy of left vs. right, respectively. The 1:1 coupling mentioned near the end of the manuscript is completely trivial — any two things that occur simultaneously with any periodicity are 1:1 coupled.

*In the revision, I have a separate Section (Section 4) that deals with the predictions of the theory. The results show evidence for a 1:8 coupling between IAF and HR and for binary multiple coupling ratios with breathing and also for BOLD fluctuations.*

*With respect to the 1:1 coupling, the Reviewer is right that this should not be taken as evidence for the theory. In the revision, I take this just as an example for a copling principle in body oscillations.*

8. The seven figures make mountains out of molehills. For instance, figure 4 uses a strangely scaled y-axis to make a pretty picture out of what should simply be displayed as a line on a log scale, while figure 7 takes half a page to show that the left foot hits the ground on every second step.

*I have changed several Figures. Fig. 3 is new, Fig. 4 is revised (now Fig. 5) – as the Reviewer has suggesed – using a log scale. Fig. 7 is still included, because I think it is nice to illustrate that even walking reflects a system of coupled oscillators.*

9. It is valuable to note that the ERP likely represents transient phase alignment. However, since this alignment is transient, it must represent the alignment of oscillations with no sustained systematic period-nesting.

*Thus is true. In the revision, I emphasize this point in Section 3.3.*

Response to Reviewer 3

Dear Reviewer, many thanks for your helpful comments. Here, I give an overview about how I have dealt with them. I use your numbering of the comments for my response, and discribe, how and where I have made changes in the revision.

1. Pg.2 (abstract) – perhaps the first couple of sentences could make more explicit that this paper will argue that the principles already hypothesized in brain oscillations will be extended to other non-brain oscillations? (it would be nice to have a slightly better link from brain to brain-body).

*Many thanks for your comment. This is an important point! I have made respective changes in the Abstract and the first line in Section 1. But most importantly, I have re-organized most Sections of the revised ms according to your suggestion, to extend the principles found for brain oscillations to body oscillations. I have changed the title (to: The Frequency Architecture of Brain and Brain-Body Oscillations: An Analysis) and reorganized the ms in an attempt to focus on three separate but closely related issues, on (i) coupling principles in brain and (ii) body oscillations and (iii) to present my theory as a result of the analyses of (i), which makes predictions on (ii). All Sections are revised and reorganized accordingly*

2. Pg.2 2nd line of intro – suggest inserting “cortical oscillatory” between “many” and “frequencies”.

*Done as suggested (see page 2, Section 1,2^nd^ sentence)*

3. Pg.3 3rd para – the notion of a ‘binary hierarchy’ could do with unpacking,

to make it clearer to the reader at this point.

*I am sorry, I do not know, what the reviewer means with ‘unpacking’.*

4. Pg4 7 lines from bottom – “without the influence of theta” – it would be

useful for the reader to be told what the possible consequences of this

would be.

*I have erased that sentence. You are right, it was not clear, because the emphasis is on the double modulation by theta and gamma.*

5. Pg7, 3rd para – I’m a little worried (especially given the author’s extensive work in this field) that the brief descriptions of the functional significance of the main EEG bandwidths come across as a little simplistic and may encourage somewhat lazy interpretation of data sets from less experienced EEG practitioners. Having said that, it is not for this paper to

answer all such issues, I just wanted to bring it to the author’s attention.

*Many thanks, you are completely right. This issue is now discussed in Section 2 (page 3 and following pages)*

6. Pg13 section 4 (and elsewhere) – the author may also like to consider extending the scope of his theory to incorporate the electrogastrogram (EGG)? There is increasing interest in the link between the CNS and the enteric nervous system and one notable recent publication described a 1:1 relationship between the phase of the slow gastric rhythm and the amplitude of the cortical alpha rhythm (Richter et al, 2016, NeuroImage).

Given that the frequency of normal gastric slow waves is about 3 cycles per minute (Yin & Chen, 2013, JNM), by my rough calculations, that puts it somewhere between BOLD IMF 1 & BOLD IMF 2 in figure 4.

*Again, many thanks for this comment. I have included gastric waves (in what is now Fig. 5) and have added a new Section (Section 5.2; page 24) where I refer to the interesting work of Richter and coleagues in Section 5.2 (page 24).*

7. Page 16, 3rd para – for me, the section about the ‘length of the R-R interval’ could be made a little clearer (given the not obvious use of the term ‘amplitude’ to describe it).

*Yes, that’s a good point. I have added the following sentences (now on page 21, 3^rd^ paragraph): …..This relationship can be illustrated by a diagram, in which for each heart beat (at time t shown at the x-axis) the corresponding cycle length (in ms) is plotted on the y-axis. Fluctuations in the envelope of cycle lengths reflect m:n coupling (in this case with breathing) and is known as heart rate variability (HRV)…….*

8. Page 16, para 4, final sentence – arguably this change in HR also varies according to the locus of attention (i.e. internal vs external). Perhaps a brief inclusion of the Lacey’s theories (e.g. 1967, 1970) here would be helpful (and may also tie in the link between baroreceptor range and alpha activity – e.g. Wolk & Velden, 1987)?

*I mention the work of Lacey in the 2^nd^ paragraph from the bottom on page 22, as suggested.*

9. Pg18, 1st para – DMN – is it worth also making clar here that DMN is considered an internally focused state (which would fit well with DMN being associated with upper alpha) and would link well to the 1st para on page 20?

*Many thanks for this comment. Done as suggested (page 19, 3^rd^ paragraph, line 15)*

10. Spellings and typos

*Many thanks! Corrected as suggested*

2^nd^ Editorial Decision	19/06/18

_____________________________________________________________________________________

The Frequency Architecture of Brain and Brain-Body Oscillations: An Analysis

Dear Prof. Klimesch,

First, my sincere apologies for the long delay in getting word to you about your revised manuscript – the fault is entirely with me. I have two returned reviews at this stage and as you will read in the attached comments, there remains considerable ground to be covered if the reviewers are to be convinced that this manuscript is ready for publication here at EJN. You will also find that both reviewers are divided in their opinion of the work, and this has in part contributed to the long period of deliberation. Reviewer #1 is relatively positive about the revisions, but nonetheless lists a large number of generally solvable issues that will still need to be addressed. The second reviewer is considerably more critical and continues to have issues with some of the central claims of the paper. He points out that ascribing specific cognitive domains to a given frequency band is likely to be a major oversimplification, and suggests some ways to reconceptualize this.  He also worries that a number of the statements made about phase, rhythmicity, and phase-amplitude coupling, rely on circular reasoning. He points to important work that should be considered when making some of the arguments about oscillatory coupling.  There are entire sections of the paper where claims are made that are not substantiated by the extant literature.  So, we find that much work remains to be done if your paper is to be considered suitable here at EJN. We are certainly willing to entertain another revision if you feel that you can adequately and forthrightly address the serious issues that have been raised.

If you do decide to take on the challenge of another revision, please embolden or underline major changes to the text so they are easily identifiable and DO NOT leave 'track change' formatting marks in your paper.  Please ensure that you provide a text and a figure file for the Graphical Abstract (as detailed in the instructions below).  When carrying out your revisions please refer to the checklist below and visit the EJN author guidelines at www.ejneuroscience.org

When finalized, please upload your complete revised manuscript onto the website, as a Word file (.doc, or .docx).  Please also ensure that a complete set of tables and figures is included as separate files, even if these have not changed from the originals.  At this stage it is necessary to provide high resolution figures.  Please see important instructions below.

Please go into https://mc.manuscriptcentral.com/ejn - Author Centre - manuscripts with decisions where you will find a 'create a revision' link under 'actions'. We ask that you please indicate the way in which you have responded to the points raised by the Editors and Reviewers in a letter. Please upload this response letter as a separate Word (.doc or PDF) file using the file designation "Authors' Response to Reviewers" when uploading your manuscript files.  Please DO NOT submit your revised manuscript as a new one.  Also, please note that only the  Author who submitted the original version of the manuscript should submit a revised version.

If you are able to respond fully to the points raised, we would be pleased to receive a revision of your paper within 12 weeks.

Thank you for submitting your work to EJN.

Kind regards,

John Foxe &
Paul Bolam
Editors in Chief, EJN


Reviews:

Reviewer: 1

Comments to the Author
<p><strong>The author has addressed my concerns from the previous  submission. I have some lingering questions about the degree of falsifiability  (see my responses to points 1, 3 and 9 below), but I am much less concerned  about this issue that I was initially. Given the scope of an opinion article, I  think the argument supporting the theory and the predictions thereof are  sufficiently clear in this revised manuscript. Minor revisions to add  discussion of the remaining issues raised below may improve the article, but are  not critical in my opinion.</strong></p>
<p>Main concerns (author’s reply in italics, mine in bold):</p>
<p>1. It is not clear, how to falsify the theory. How  "far" would a frequency have to be from the predicted value to  invalidate the theory?</p>
<p><em>This issue is addressed in Section 4.1 that deals with  the predictions of the theory (see page 15 and following). In short, the theory  is falsified if the frequency of a reported oscillation falls outside the  respective frequency band that is predicted by the theory.</em></p>
<p><strong>Great, thanks. It’s clearer to me now what observations  would be unexpected under this theory. One lingering question I have is if  other coupling factors would lead to observations of frequencies in the  “forbidden zone” outside the predicted frequency bands. E.g., suppose the true  harmonic for coupling of frequencies was 1:3 (or 1:5, etc.), rather than 1:2.  In that case would we see frequencies outside the range predicted by the 1:2  theory (and if so, with what probability?), or are the predicted frequency  bands wide enough that they would still contain the frequencies generated by a  1:3 model? It’s a bit tricky to intuit because the theory allows some degree of  translation on the frequency axis (i.e., 30 Hz would be out of range if alpha  was 10 Hz, but 33 Hz would be in range if alpha was 11 Hz).</strong></p>
<p>2. I'm unclear on the details of exactly how this theory is  supposed to play out in an individual. If my heart rate goes up, does my alpha  center frequency also go up? </p>
<p><em>This issue is addressed in section 4.1.4. about the  covariation of brain and body oscillation (page 20 and following). In brief:  Yes, frequencies are predicted to covary but not in an 1:1 manner. They may  jump e.g., to an 1:2 ratio (as is the case for breathing, see page 16) or to an  irrational ratio reflecting decoupling</em>.</p>
<p><strong>The idea here is also clearer to me now. I get the  impression the cross-frequency coupling is not supposed to be extremely rigid,  but is a bit more of a “force” nudging frequencies around in tandem.</strong></p>
<p>3. Why do brain oscillations have bandwidth? There isn't  really any room in this theory for bandwidth that I can see.</p>
<p><em>Sorry for this misunderstanding, I have put much effort  in clarifying this crucial issue. Sections 1-3 are revised accordingly. The  basic arguments (summarized in section 1) are (i) that not all frequencies are  equal,(ii) that center frequencies of traditional EEG bands reflect ‚preferred‘  frequencies that are associated with specific processing domains (see section  2). (iii) Most importantly, the analysis of cross frequency coupling (section  3) shows that phase is the driving force for coupling. The central argument  here is that phase can establish its impact – physiologically as well as  mathematically – only, if its frequency is a dominant oscillation (even with  large jitter) within a certain frequency band. In a broad band with ‘equal  frequencies’ that lacks a distinct oscillation, the phases of different  frequencies will tend to cancel each other due to the superposition of this  frequencies (as measured by standard EEG/MEG recordings). (iv) Finally and most  importantly, center frequencies may shift to couple or decouple with other  frequencies (see Sections 3.3.1 and 3.3.2 page 12 and following).</em></p>
<p> <strong>Ok; I think I  understand the framework now.</strong>

</p>
<p>Specific Comments</p>
<p> 1. The author writes,  “The binary hierarchy…. represents a universal scale free power law.” The term,  “scale free” implies that no one scale is more important than another. Yet the  author argues at other points that heart rate is “the most [important] body  oscillation” . </p>
<p><em>Yes it’s both: scale free (because of the power function)  and scaled because of the introduction of a scaling factor, s (Formula 2). Only  because of the relationship between s and HR, HR may be assumed the most  important body oscillation. In the revision I have separated two questions: (i)  Is HR part of the binary hierarchy (Section 4.1.1, page 15) and (ii) what are  the implications of the HR scaling assumption (Section 6.2. page 32 and  following). I have done this, because the HR scaling assumption is not an assumption  underlying the suggested theory, but a possible implication.</em></p>
<p><strong> I’m still not  convinced that any one physiological process in this hierarchical scheme needs  to be considered more “fundamental” than any other, but I don’t feel strongly  about it. </strong></p>
<p>2. The author states “it will be argued that there are  (physiologically) only two coupling principles, (i) amplitude (envelope)  coupling… and (ii) phase coupling…”. However, I do not find evidence arguing  for this claim to follow. The manuscript does include examples of the two types  of coupling provided, but does not describe the types of coupling that are  *excluded* by the claim (e.g., amplitude-amplitude correlations --am I correct  that this is the only remaining possible combination? </p>
<p><em>I refer to this issue in Section 3 (page 5), first  paragraph.</em></p>
<p><strong>Thanks.</strong></p>
<p>3. I am left wondering what passive observations would have  been like that could have disproved it. For example, what would my heart rate  and alpha frequency need to be for us to conclude that the binary hierarchy  can’t be correct? </p>
<p><em>The answer is: if the ratio between individual HR and  individual alpha frequency (IAF), measured in awake resting subjects, would not  be 1:8 (see Section 4.1.1, pages 15,16)</em></p>
<p><strong>This is one of the tougher ones for me to get on board  with. Why 1:8 rather than 1:4 or 1:16, or even 1:256? The author might argue  that 2^3 should be the octave for alpha, but then the argument is made  elsewhere in the manuscript that any particular octave can be occupied by  different physiological functions across species (section 6.2). Therefore, what  would exclude predicting a 1:4 relationship in humans (i.e., a resting heart  rate of 144 bpm, or a brain oscillation around 20Hz that fills the functional  role of what we now call the alpha rhythm)? However, given the response to my  first stated concern, perhaps a falsifying observation under this theory would  be e.g. a heart rate of 1.8 Hz for an IAF of 10 Hz, which would be a 9:50 ratio  and fall outside of the predicted bandwidth for one or the other process  (depending on which is used as a reference).</strong></p>
<p>4. All of the evidence given relies on average values for a  species, but I believe the author’s hypothesis should make strong predictions  about the spacing of brain and body oscillation frequencies within individuals,  correct? Has anyone looked at that?</p>
<p><em>The reviewer is right, but there are almost no studies on  this issue. Nonetheless, I found some recent work which plays an important  role. This e.g., is Siebenhüner et al. (2016), see e.g. Fig. 3b,c and Gutmann  et al. (2018), see Sections 3 and Section 4.1.1(pages 15,16)</em></p>
<p><strong> Good. This  addition helps. The reference list should be updated to include the Gutmann  reference.</strong>

 

 5. I am having difficulty following the reasoning at the  bottom of page 2: “The argument here is that in all examples, phase is the  driving force for coupling. The central conclusion from these findings is that  phase can establish its impact… only, if its frequency exhibits a comparatively  small jitter. In a broad band the phases of different oscillations with  different frequencies will tend to cancel each other. This is taken as strong  evidence for the existence of more or less distinct center frequencies.” I  believe the argument is that because theory requires the phases to be stable,  that that serves as evidence for what’s going on in nature. This strikes me as  the reverse of the usual tack, for which scientists take observations of what  goes on in nature as evidence for or against theory. In fact, when we measure  the power spectrum of brain activity we usually do not see narrow-band peaks,  especially e.g. in the gamma band. Does the author have experimental  observations to bolster this argument?</p>
<p> <em>The theory does  not require stable phases or stable frequencies. Frequency jitter is the  essence of coupling and decoupling. Both principles refer to specific frequency  ratios. Please see my response to main point 3 above and the line of  argumentation in Sections 1-3.</em></p>
<p><strong>Ok.</strong></p>
<p>6. Another good example of the behavioral correlates of  cross-frequency phase-amplitude coupling that the author might consider  reviewing was provided by Fiebelkorn, et al. (2013), NeuroImage, vol. 69.</p>
<p><em>Many thanks for this hint, this work is now included (see  page 11).</em></p>
<p><strong>Thanks. The reference list should be updated as well. </strong></p>
<p>7. Alpha peaks appear usually during…. Frontal midline  theta… can be observed during…. Sleep spindles are associated with. These  claims need citations. For the alpha claim, one source the author might  consider is a review from Foxe and Snyder (2011).</p>
<p> <em>Many thanks, you  are right. I have now referred to several studies/reviews, including the  suggested work by Foxe and Snyder (2011). See pages 3 and 4.</em></p>
<p> <strong>Thank you.</strong></p>
<p>8. The author writes, “This critical issue is closely linked  to two questions, to the number of different frequency hierarchies (see above)  ………The author then goes on to describe some potential “explanations” of  frequency jitter. One factor that was not discussed, however, is that our  recording electrodes are integrating over relatively large areas of space……</p>
<p> <em>Many thanks, you  are right again. This issue is emphasized in Section 3.3.2, second paragraph on  page 14.</em></p>
<p> <strong>Thanks.</strong></p>
<p>9. Following-up on the previous issue, at several points in  the manuscript it seems as though the author is suggesting that the “scaling factor”  for formula 1 is universal within an individual. How critical is this for the  author’s central premise? I could imagine that some neural cell assemblies  might be coupled more or less strongly to others and therefore could have some  degree of freedom with respect to the exact value of the scaling factor at any  given time. For an oversimplified example, maybe the left hemisphere resonates  at 10.1 Hz whereas the right hemisphere prefers 12.3 Hz (see also, Groppe et  al., 2013, NeuroImage, vol. 79, who used ECoG to find “substantial variability  in the dominant frequencies cortical regions…”; and of course, see also  Klimesch et al., 1993, Brain Topography, vol. 5). Could frequency jitter  actually not be a problem, but rather a genuine use of frequency sub-bands?  Would this perspective undermine the whole “binary hierarchy” framework? What  is the role of bandwidth in this theory?</p>
<p> <em>Many thanks, these  are important issues. In the revision, I emphasize that the binary hierarchy  theory (regarding brain oscillations) applies to ‚preferred frequencies‘, which  are termed frequency domains only (see Section 2). With respect to subbands: I  refer e.g., to high alpha and high theta in work showing peaks in these  subbands in memory tasks (see Section 3 pages 8,9 and Fig. 3c). The point here  is that frequencies may shift in a task dependent manner to couple (or  decouple). When they couple, they stay within the predicted bands, otherwise  the fall outside the bands.</em></p>
<p><strong> Ok, the reasoning  is clearer here now. This does tread closely to unfalsifiability, however. That  is, anything conforming to the theory can be explained as coupling and anything  violating the theory can be explained away as decoupling. Coupled and decoupled  oscillations are predicted to have different functional consequences, though,  so I think this is fine.</strong></p>
<p>10. How salient of a force does the author suppose the  “binary hierarchy” to be? Is this more of a developmental principle that stakes  out where on the spectrum each body function will roughly fall, but then from  moment-to-moment their frequencies are somewhat independent? In other words,  does the author predict that my individual alpha frequency should be about  twice as fast if I measure it while I’m on a treadmill with a heart rate of 144  bpm as opposed to when I’m sitting quietly at 72 bpm? This seems unlikely.  While I'm not personally well-versed on this issue, I know there is a small,  but growing, literature on EEG changes during exercise; For example, Gutmann et  al., 2015, Neural Plasticity, vol. 2015, measured individual alpha peak  frequency before and immediately after exercise, and they did report an  increase (in only one of two exercise conditions), but the size of that effect  was only a quarter of a Hertz, not nearly what would be predicted by a binary  hierarchy based on heart rate. What's more: the peak frequency remained  elevated after ten minutes of rest, presumably after heart rate would have  decreased to near resting levels, which also doesn't seem to fit with the  binary hierarchy theory.</p>
<p><em>Many thanks! There is a very relevant recent publication  from Gutmann (Gutmann et al. 2018), which plays an important role in the  revised ms. Please see Section 4.1.(pages 15,16), but also Section 4.1.4, page  20.</em></p>
<p> <strong>Ok.</strong></p>
<p>11. It is not clear which of the figures depict measured  data, and which are merely cartoon examples. My impression is that only figure  6 depicts measured data and the rest are all just illustrations. Is that  correct? This should be made explicit in the figure legends. </p>
<p><em>Done as suggested</em></p>
<p><strong> Thanks.</strong></p>

Reviewer: 4

Comments to the Author
Despite the revisions, the manuscript is still unclearly written and comes off as unclearly thought out. The concerns that Reviewer 1 and Reviewer 2 expressed are still issues for the revised manuscript. I have to admit that the central hypothesis, of a binary hierarchy of brain oscillations, does not seem worth making in the form in which it’s presented. This is a shame, because there are some really interesting ideas in the paper, and bringing together the growing literature on the interactions between brain and body oscillations is a valuable endeavor. I believe that stating the hypotheses in the paper with more subtlety and clarity, and removing the claims that “make mountains out of molehills”, as Reviewer 2 stated, is necessary before publication.

Here are some of the conceptual issues with the manuscript.
1) The paper conflates “frequency” and “rhythm” throughout. In fact, brain rhythms are rhythmic dynamics exhibited by specific brain cells and/or brain circuits, as a result of critical timescales that arise in those cells/circuits. Two brain rhythms with the same or similar frequencies can arise from entirely different mechanisms, and have very different functional meanings (Cannon et al., EJN 2014) – consider the many different mechanisms producing beta-range oscillations in pathological and physiological conditions (Engel & Fries, Current Opinion in Neurobiology 2010), and the different functional significances of frontal midline theta and hippocampal theta. Thus, associating different frequencies with different cognitive domains is a vast oversimplification. The functional meaning of an oscillation comes from the timescales that give rise to it in a particular circuit and brain area. Are these timescales grouped together? How and why might they be grouped together – why do two different rhythms, in two different brain areas, utilized for two different purposes, have the same frequency? Is it a coincidence, or not? I wish these questions were addressed in the manuscript. In the absence of addressing them, the work needs to make clear that the frequency ranges into which brain oscillations fall are just that – relatively arbitrarily defined intervals used to group observed brain oscillations – and that ongoing research into the mechanisms of brain oscillations is making these frequency domains increasingly irrelevant. If there is an argument to be made that these frequency bands have a functional significance despite the functional heterogeneity of the oscillations that fall within them, that’s interesting – but assuming functional heterogeneity within frequency bands is downright inaccurate.
2) If the paper discussed the very rough distribution of timescales in the brain that give rise to oscillatory phenomena, that would make a bit more sense. Even then, the roughness of this distribution would require a different treatment than the attempted mathematical precision of the current manuscript. It is possible that the timescales in the brain are distributed in a roughly dyadic manner, and the idea of predicting body oscillations from this distribution is an interesting one. But, as the manuscript repeatedly mentions, the frequencies of oscillations vary over time, vary between individuals, and are altered by coupling or decoupling with each other. Given this, the effort expended to pin oscillations to a mathematically precise frequency hierarchy seems to be wasted. Furthermore, there is no attempt to disentangle the causality of relationships between oscillatory frequencies – do relationships between oscillatory frequencies allow coupling, or do they reflect coupling? Answering this question would require an understanding of the mechanisms of individual oscillations, and the mechanisms of coupling between them.
3) In the absence of a clear description of the mechanisms of rhythmicity, and an understanding of their variability, unstated assumptions about the function of rhythms in communication muddy the central hypothesis. For example, the statement “m:n phase to phase coupling … is optimal for harmonic frequency ratios only” is unclear. What is the meaning of the term optimal here? When the frequencies of two oscillations are not the same, then phase-locking between them is a claim about the relationship between their instantaneous phases over time – that they should be proportional, for instance. It is not obvious why the constant of proportionality needs to be an integer. The manuscript should make it clear how rhythms enable communication, and not only whether but why this puts constraints on rhythmic frequencies.
4) I find the argument about phase – that “phase can establish its impact … only if its frequency is a dominant oscillation … within a certain frequency band” – to be circular, unclear, and unconvincing. What does it mean for an oscillation to be “a dominant oscillation … within a certain frequency band”? A well-defined instantaneous phase exists only for a waveform that is sufficiently oscillation-like. Is the argument here that oscillations exist? That brain oscillations are not colored noise? This seems pretty self-evident. It is also hard to imagine what could be meant by the claim that “there is no evidence for the existence of different frequency domains” in rodents.
5) It’s unclear to me why the manuscript uses the term “amplitude (envelope) modulation” in place of “phase-amplitude coupling”, a more specific and commonly-used term. The most obvious interpretation of the term “amplitude modulation” is amplitude comodulation, or the covariation in amplitude between two oscillations at different frequencies. In addition, there are several misconceptions about phase-amplitude coupling in the manuscript.
a. First, the idea that the coupling of spiking or multi-unit activity to an ongoing oscillation counts as phase-amplitude coupling is against the entire spirit of defining cross-frequency coupling as distinct from rhythmicity. There are many quantities that covary with the phase of rhythms in the brain and body – this is, indeed, how and why most of these rhythms are defined and identified in the first place. To claim that all of these phenomena are examples of phase-amplitude coupling reduces the term to a vacuous generality. Phase-amplitude coupling – like all forms of cross-frequency coupling – requires two rhythms to be coupled to each other. An arrhythmic phenomenon like spiking is just a quantity that is rhythmically modulated.
b. Secondly, respiratory sinus arrhythmia is an example of phase-frequency coupling – the phase of the respiratory rhythm modulates the frequency of the heartbeat. It is not phase-amplitude coupling, by any definition of phase-amplitude coupling that is specific enough to remain sensible. The length of the R-R interval is not the amplitude of anything.
6) In the revised manuscript, in Section 3.3, the manuscript now lists the types of possible cross-frequency coupling. However, there is still no attempt made to justify the claim that “phase is the driving force for coupling”. Not that this claim is wrong – but the paper neither explains nor provides evidence for the claim. It would be great if the manuscript clearly stated what this could mean, and whether or not there is evidence for it.
7) The idea that the relationship between amplitude and period gives rise to the 1/f spectrum of brain electrical activity is an interesting one. But there is little discussion of whether all the various mechanisms of brain oscillations exhibit the same relationship between amplitude and period on a cycle-by-cycle basis. There is also no attempt to explain why the proportionality between amplitude and period should be constant across oscillations of different frequencies and mechanisms. The author should also make contact with the literature discussing the relationship between scale-free dynamics and oscillatory coupling (He et al, Neuron 2010; Pittman-Polletta et al. Biological Reviews 2013) Finally, the corresponding figure is erroneous – if amplitude and period are linearly related, then amplitude and frequency are already reciprocally related (not just negatively correlated).
8) The manuscript states “brain and body oscillations obey the same coupling principles and can be described by a single system” as if this were news. But any two oscillatory subsystems of a larger system can display the kinds of relationships between amplitude and phase that the author describes – this is part of why these measures of cross-frequency coupling are useful, and why they are already being applied to study the relationships between brain and body oscillations. Furthermore, the fact that brain and body oscillations are part of a single system is perhaps worth emphasizing in contrast to persistent dualistic notions of brain and body, but it is not a discovery or a hypothesis – it is self-evident that brains are contained in, constrained by, and interact with the “bodies” of the biological organisms of which they are a part.
9) In the discussion of brain and body oscillations, the manuscript omits recent findings on respiratory-related oscillations (Tort et al. Scientific Reports, 2018). One of the major points made in the literature on RROs is that respiratory frequency varies across multiple classical frequency bands (delta and theta), and can thus be conflated with them. This seems to conflict with the central hypothesis of the paper.
10) Section 3.2 makes little sense.
a. The author’s discussion about “temporal precision” is very unclear. What is it that operates “at the temporal precision of the slow frequency” in phase-amplitude coupling? Is the author talking about the timescale on which coupling evolves? The timing of spiking? If the former, it’s experimentally unclear at what timescales cross-frequency coupling is engaged and disengaged, and theoretically unconstrained. If the latter, the author needs to discuss more carefully what the limits on spiking precision imposed by the two coupling regimes are, and where these limits come from.
b. The idea that phase coupling can be established at different phase angles, while phase-amplitude coupling cannot, is patently false. There are many examples in the literature of high-frequency oscillations coupling to different phases of low frequency oscillations.
c. In the second paragraph, the author starts discussing observations of phase-phase coupling in EEG, and states “one of the basic results” is that phase-phase coupling increases in tasks with high cognitive demands. He then states “another basic results stems from the 1:2 ratio between neighboring frequencies”. I cannot see what this basic result could be. The only thing I can conclude is that the author intends to suggest that all observations of rhythms at “about” harmonic frequencies involve phase-phase coupling. Given the highly nonstationary nature of brain oscillations, with fluctuations in frequency and phase occurring from cycle to cycle, much more evidence is required than simply a “1:2 ratio” between oscillations to demonstrate phase-phase coupling. The author, however, goes on to list a number of “binary multiple frequency ratios”, while also listing non-binary frequency ratios.
d. The idea that theta-gamma coupling between frequency bands of 4-8 and 30-50 Hz “is consistent with a 1:8 coupling ratio” drives home the unnecessary and imprecise nature of the paper’s central hypothesis. If the frequency bands within which oscillations occur is broad enough to allow oscillations to assume both binary ratios and golden mean ratios, as well as ratios that fall into neither category, then why is it worth tying them to a precisely-defined mathematical hierarchy? There is not enough information in this paper to make it clear.
11) Many of the points seem belabored, irrelevant, or too speculative.
a. Section 3.1 (on phase-amplitude coupling) doesn’t require more than one illustrative example (say, Lakatos’ binary hierarchy).
b. In section 3.1, the discussion of phase precession is an entirely unnecessary detail, and it and the associated figure should be omitted.
c. In section 3.2, the tedious listing of reports of “binary multiple frequency ratios” along with non-binary multiple frequency ratios can and should be condensed.
d. In general, the evidence supporting the main claims could be summarized much more succinctly.
e. The binary frequency hypothesis is stated in multiple places. It should be stated once, early on, and referred to later only minimally.
f. The phenomena described in section 5.4 are not related enough to the central hypothesis to include this section and the associated figure.
g. Section 6.3 – “music and the binary hierarchy” – is an interesting topic. But these ideas are not really sufficiently developed here to be included.
12) The first figure is unnecessarily complicated – the “asymmetric oscillation which may be induced by the slope of another very slow oscillation” is not important in the text, not central to the paper’s main point, and so it should not appear in the figure.

_____________________________________________________________________________________

Authors’ Response	19/08/18

_____________________________________________________________________________________

Response to Reviewer: 1

Dear Reviewer, many thanks again for your thoughtful comments/questions, to which I refer below for the revised ms. Numbering is to your previous comments/questions (which are listed together with my previous response) and my response now for the revised ms is marked in red after ‘author’s response’. Missing numbers refer to those issues, to which you had no further comments/questions.

Main concern:

1. It is not clear, how to falsify the theory. How "far" would a frequency have to be from the predicted value to  invalidate the theory?
*This issue is addressed in Section 4.1 that deals with the predictions of the theory (see page 15 and following). In short, the theory is falsified if the frequency of a reported oscillation falls outside the respective frequency band that is predicted by the theory*.
Great, thanks. It’s clearer to me now what observations would be unexpected under this theory. One lingering question I have is if other coupling factors would lead to observations of frequencies in the  “forbidden zone” outside the predicted frequency bands. E.g., suppose the true harmonic for coupling of frequencies was 1:3 (or 1:5, etc.), rather than 1:2.  In that case would we see frequencies outside the range predicted by the 1:2  theory (and if so, with what probability?), or are the predicted frequency  bands wide enough that they would still contain the frequencies generated by a  1:3 model? It’s a bit tricky to intuit because the theory allows some degree of  translation on the frequency axis (i.e., 30 Hz would be out of range if alpha  was 10 Hz, but 33 Hz would be in range if alpha was 11 Hz).

Author’s response: Many thanks, this comment is very helpful. The following two arguments are important here. (i) The forbidden zones refer to *neighboring frequency* domains. As an example, a frequency of 15 Hz lies in the forbidden zone relative to alpha and beta, which means that 15 Hz can hardly synchronize with beta or alpha. But a 15 Hz rhythm can well 1:3 couple with theta (5 Hz) or 1:6 couple with delta (2.5 Hz). (ii) If the true harmonic for coupling between neighboring frequency domains (as a general role) would be e.g., 1:3, then the entire hierarchy would be widened and shift e.g., from delta with e.g., 2.5 Hz to neighboring frequencies of 7.5 Hz and 0.83 Hz. In this example, we would miss theta and alpha of about 5 and 10 Hz, and we could not predict the frequencies of body oscillations from brain oscillations. This indicates that only the binary hierarchy allows a faithful description of brain and body oscillations

I have added clarifications regarding (i) in the third paragraph in Section 3.2 (page 8), and regarding (ii) in the first paragraph in Section 4.1 (page 15) in the revised ms (marked by underlining)

Specific Comments
1. The author writes,  “The binary hierarchy…. represents a universal scale free power law.” The term,  “scale free” implies that no one scale is more important than another. Yet the author argues at other points that heart rate is “the most [important] body oscillation”

*Yes it’s both: scale free (because of the power function) and scaled because of the introduction of a scaling factor, s (Formula 2). Only because of the relationship between s and HR, HR may be assumed the most important body oscillation. In the revision I have separated two questions: (i)  Is HR part of the binary hierarchy (Section 4.1.1, page 15) and (ii) what are  the implications of the HR scaling assumption (Section 6.2. page 32 and  following). I have done this, because the HR scaling assumption is not an assumption underlying the suggested theory, but a possible implication.*

I’m still not convinced that any one physiological process in this hierarchical scheme needs to be considered more “fundamental” than any other, but I don’t feel strongly about it.

Author’s response: You are right, in the hierarchy all frequencies are ‘equal’ in a mathematical as well as in a biological sense. This is the scale free part of formula. What I mean with ‘fundamental’ refers to an interesting property of formulas (1) and (2) which is that the scaling factor s itself can be identified as a physiological oscillation, namely HR. In a mathematical sense, s could be any quantity, not necessarily a brain or body oscillations. I have erased the terms ‘fundamental’ and ‘most important’ with respect to HR.

3. I am left wondering what passive observations would have been like that could have disproved it. For example, what would my heart rate and alpha frequency need to be for us to conclude that the binary hierarchy  can’t be correct?

*The answer is: if the ratio between individual HR and  individual alpha frequency (IAF), measured in awake resting subjects, would not  be 1:8 (see Section 4.1.1, pages 15,16)*

This is one of the tougher ones for me to get on board with. Why 1:8 rather than 1:4 or 1:16, or even 1:256? The author might argue that 2^3 should be the octave for alpha, but then the argument is made elsewhere in the manuscript that any particular octave can be occupied by different physiological functions across species (Section 6.2). Therefore, what would exclude predicting a 1:4 relationship in humans (i.e., a resting heart  rate of 144 bpm, or a brain oscillation around 20Hz that fills the functional  role of what we now call the alpha rhythm)? However, given the response to my first stated concern, perhaps a falsifying observation under this theory would  be e.g. a heart rate of 1.8 Hz for an IAF of 10 Hz, which would be a 9:50 ratio  and fall outside of the predicted bandwidth for one or the other process  (depending on which is used as a reference).

Author’s response: I think you already answered your question above, when you say ‘a falsifying observation under this theory would be e.g., a heart rate of 1.8 Hz. I also think your question here is related to your main concern (Comment 1), which I have answered above (see (ii) to your Comment 1). But in short: The answer to your question also is related to the identification of frequency domains. According to formula (2) the frequency domains for i = 0, 1, 2, 3, …. can be identified with HR (i=0), delta, theta and alpha (i=1,2, and 3) respectively. Thus, the ratio between HR and alpha must be 1:8. Or in other words, the answer to your questions is given by formula (2) which predicts a 1:8 ratio between HR and alpha which is empirically testable (See the Gutmann et al. (2018) results, which are described in Section 4.1.1.)

4. The reference list should be updated to include the Gutmann  reference. Author’s response: Many thanks, done as suggested. Reference is marked by underlining on page 37.

6. The reference list with respect to Fiebelkorn, et al. (2013) sould be updated as well. Author’s response: Many thanks, done as suggested. Reference is marked by underlingn on page 36.

9. Following-up on the previous issue, at several points in the manuscript it seems as though the author is suggesting that the “scaling factor”  for formula 1 is universal within an individual. How critical is this for the  author’s central premise? I could imagine that some neural cell assemblies might be coupled more or less strongly to others and therefore could have some degree of freedom with respect to the exact value of the scaling factor at any  given time. For an oversimplified example, maybe the left hemisphere resonates  at 10.1 Hz whereas the right hemisphere prefers 12.3 Hz (see also, Groppe et  al., 2013, NeuroImage, vol. 79, who used ECoG to find “substantial variability  in the dominant frequencies cortical regions…”; and of course, see also  Klimesch et al., 1993, Brain Topography, vol. 5). Could frequency jitter  actually not be a problem, but rather a genuine use of frequency sub-bands?  Would this perspective undermine the whole “binary hierarchy” framework? What  is the role of bandwidth in this theory?

*Many thanks, these are important issues. In the revision, I emphasize that the binary hierarchy  theory (regarding brain oscillations) applies to ‚preferred frequencies‘, which  are termed frequency domains only (see Section 2). With respect to subbands: I  refer e.g., to high alpha and high theta in work showing peaks in these  subbands in memory tasks (see Section 3 pages 8,9 and Fig. 3c). The point here is that frequencies may shift in a task dependent manner to couple (or  decouple). When they couple, they stay within the predicted bands, otherwise  the fall outside the bands.*
Ok, the reasoning  is clearer here now. This does tread closely to unfalsifiability, however. That is, anything conforming to the theory can be explained as coupling and anything  violating the theory can be explained away as decoupling. Coupled and decoupled  oscillations are predicted to have different functional consequences, though,  so I think this is fine.

Author’s response: I think the answer to your question here relates to task specific frequency shifts. As an example, if in a movement task, alpha (or more precise the mu-rhythm) slows to e.g., 8 Hz in task relevant brain areas, the binary hierarchy is predicted to slow down (ideally to 2, 4, 8, 16 Hz). In the first paragraph in Section 4.1.4 (page 20) I have inserted a respective clarification.

Response to Reviewer: 4

Summary of the author’s responses: Dear Reviewer, many thanks again, but I am sorry to say that I see a deep misunderstanding/misconception in your comments. Many comments (concerning ‘oversimplification’, ‘harmonic ratios’, ‘circular argumentation’, HRV, ‘erroneus’ Fig. 4, RRO and Lakatos’ ‘binary’ hierarchy; please see my detailed responses below) are simply wrong. Other comments reflect a deep misunderstanding of my argumentation. For me, this is not surprising, when I consider Comment 3, where the Reviewer asks why “m:n phase to phase coupling … is optimal for harmonic frequency ratios only”. The Reviewer questions a simple mathematical fact! Arguments about numerical frequency relationships are core issues in my ms, which are built on the optimal integer (harmonic) and the disadvantageous golden mean relationships. Ignoring/not knowing/not recognizing these simple mathematical facts cannot allow the Reviewer understanding/appreciating my argumentation. An obviously related problem is that the Reviewer raises many fundamental questions (which are interesting in itself) but are simply beyond the scope of the submitted ms and also irrelevant for my argumentation. As an example, the Reviewer thinks my analysis (regarding the frequency architecture) can be done only when ‘….understanding the mechanisms of individual oscillations, and the mechanisms of coupling between them.’ (see Reviewer’s Comment 2) and requires a clear description of the mechanisms of rhythmicity (Comment 3). These issues are not within the scope of my ms. My analysis refers to numerical frequency relationships and is based on simple mathematical facts, which allow to state important constraints about coupling. In sum, I am sorry to say that almost all of the Reviewers comments are either wrong or not to the point. The only thing, I could do (besides correcting a few misunderstandings), was to refine the overview of my argumentation in the Introduction (Section 1) particularly regarding the functional role of phase and the frequency specificity of oscillations.

Comments to the Author
Despite the revisions, the manuscript is still unclearly written and comes off as unclearly thought out. The concerns that Reviewer 1 and Reviewer 2 expressed are still issues for the revised manuscript. I have to admit that the central hypothesis, of a binary hierarchy of brain oscillations, does not seem worth making in the form in which it’s presented. This is a shame, because there are some really interesting ideas in the paper, and bringing together the growing literature on the interactions between brain and body oscillations is a valuable endeavor. I believe that stating the hypotheses in the paper with more subtlety and clarity, and removing the claims that “make mountains out of molehills”, as Reviewer 2 stated, is necessary before publication.

Author’s response: When the Reviewer says that the binary hierarchy theory ‘does not seem worth making in the form in which it’s presented’ you have to say why. I show that this theory allows a faithful description of (i) the center frequencies of traditional EEG frequency bands, (ii) their bandwidths and (iii) allows to predict the frequencies of body oscillations. Is this nothing?

Here are some of the conceptual issues with the manuscript.
1)      The paper conflates “frequency” and “rhythm” throughout. In fact, brain rhythms are rhythmic dynamics exhibited by specific brain cells and/or brain circuits, as a result of critical timescales that arise in those cells/circuits. Two brain rhythms with the same or similar frequencies can arise from entirely different mechanisms, and have very different functional meanings (Cannon et al., EJN 2014) – consider the many different mechanisms producing beta-range oscillations in pathological and physiological conditions (Engel & Fries, Current Opinion in Neurobiology 2010), and the different functional significances of frontal midline theta and hippocampal theta. Thus, associating different frequencies with different cognitive domains is a vast oversimplification. The functional meaning of an oscillation comes from the timescales that give rise to it in a particular circuit and brain area. Are these timescales grouped together? How and why might they be grouped together – why do two different rhythms, in two different brain areas, utilized for two different purposes, have the same frequency? Is it a coincidence, or not? I wish these questions were addressed in the manuscript. In the absence of addressing them, the work needs to make clear that the frequency ranges into which brain oscillations fall are just that – relatively arbitrarily defined intervals used to group observed brain oscillations – and that ongoing research into the mechanisms of brain oscillations is making these frequency domains increasingly irrelevant. If there is an argument to be made that these frequency bands have a functional significance despite the functional heterogeneity of the oscillations that fall within them, that’s interesting – but assuming functional heterogeneity within frequency bands is downright inaccurate.

Author’s response: With respect to the ‘conflation of frequency and rhythm’: I use –as is usual done – rhythm synonymous with oscillations. As an example, ‘alpha rhythm’ or ‘alpha oscillation’ are largely synonymous terms. And with respect to frequency, this of course is a term which refers to the ‘speed’ of a rhythm (oscillation). Nonetheless, in many cases one can speak e.g., of alpha frequency or alpha oscillation, depending on what the argumentation is (referring e.g., more to frequency estimates or e.g., to the functional meaning of a rhythm (oscillation). Exactly in this way, I am using these terms. It is the context, which defines the meaning of these terms. So, I cannot see what is wrong here. I have checked the ms again and refined a few phrases accordingly. (Note: I think the Reviewer himself confuses these terms. See my response to your Comment 2 below, where the Reviewer writes ‘…..do relationships between oscillatory frequencies allow coupling…).

Other questions you raise, ignore research on the functional meaning of oscillation (i), or are irrelevant (ii) for the argumentation in the ms.

Ad(i) The functional meaning of brain oscillations is a big and central issue in Cognitive Neuroscience and I give a brief overview in Section 2. Given the large body of research on this issue, it is not acceptable when the Reviewer simply says ‘associating different frequencies with different cognitive domains is a vast oversimplification’!

Ad(ii) Yes, there are different oscillations with similar frequencies from different sources etc. These are well known facts. Empirical research has developed different methods to deal with these facts. I just mention the use of source reconstructed signals, data driven methods for selecting task relevant (reactive) frequencies, etc. But these questions are not the topic of the present article. Nonetheless, I refer on research that, of course, took care of these issues. A discussion of these issues is beyond the scope of the present ms.

2) If the paper discussed the very rough distribution of timescales in the brain that give rise to oscillatory phenomena, that would make a bit more sense. Even then, the roughness of this distribution would require a different treatment than the attempted mathematical precision of the current manuscript. It is possible that the timescales in the brain are distributed in a roughly dyadic manner, and the idea of predicting body oscillations from this distribution is an interesting one. But, as the manuscript repeatedly mentions, the frequencies of oscillations vary over time, vary between individuals, and are altered by coupling or decoupling with each other. Given this, the effort expended to pin oscillations to a mathematically precise frequency hierarchy seems to be wasted. Furthermore, there is no attempt to disentangle the causality of relationships between oscillatory frequencies – do relationships between oscillatory frequencies allow coupling, or do they reflect coupling? Answering this question would require an understanding of the mechanisms of individual oscillations, and the mechanisms of coupling between them.

Author’s response: (i) When the Reviewer says that the paper should discuss the ‘… distribution of time scales in the brain that give rise to oscillatory phenomena,’ I am left puzzled, because I think that’s what I do. The *time scale* of an oscillation is defined by its *frequency*. Right? Thus, analyzing the frequency relationships between oscillations is basically equivalent with analyzing the time scales between oscillations. So, what is wrong here? With respect to ‘frequencies of oscillations vary’: The Reviewer ignores the fact that I also give a mathematical argument for defining frequency bands and this means variation between the frequencies of oscillations (but within defined bands). Again, what is wrong here?

(ii) Regarding: ‘…..do relationships between oscillatory frequencies allow coupling…’ This is what is analyzed in the ms on an abstract, mathematical level (see my response to optimal coupling to your comment 3 below). But the Reviewer thinks an analysis of this question can be done only when ‘….understanding of the mechanisms of individual oscillations, and the mechanisms of coupling between them.’ I cannot see, why? My analysis is based on simple mathematical facts, which allow to state important constraints about coupling.

(iii) Finally, note the way the Reviewer uses the terms oscillation and frequency in the phrase ‘do relationships between oscillatory frequencies allow coupling’: From my understanding the Reviewer confuses frequency with oscillation here. The phrase should read instead ‘do relationships between the frequencies of oscillations allow coupling’

3)      In the absence of a clear description of the mechanisms of rhythmicity, and an understanding of their variability, unstated assumptions about the function of rhythms in communication muddy the central hypothesis. For example, the statement “m:n phase to phase coupling … is optimal for harmonic frequency ratios only” is unclear. What is the meaning of the term optimal here? When the frequencies of two oscillations are not the same, then phase-locking between them is a claim about the relationship between their instantaneous phases over time – that they should be proportional, for instance. It is not obvious why the constant of proportionality needs to be an integer. The manuscript should make it clear how rhythms enable communication, and not only whether but why this puts constraints on rhythmic frequencies.

Author’s response. Regarding: ‘In the absence of a clear description of the mechanisms of rhythmicity, and an understanding of their variability….’ Please see my response in (ii) to your Comment 2 above.

Regarding harmonic ratios: Sorry, you are wrong here. If one frequency has a period = p and the other a period p’= p*g (g =golden mean) than their phases will never meet (never be coupled) in the future. Questioning or asking why harmonic (integer) relationships are optimal for coupling is like questioning why 2+2 should equal 4.

Again, you are raising topics like ‘mechanisms of rhythmicity’ that are not relevant for the argumentation in the ms.

4)      I find the argument about phase – that “phase can establish its impact … only if its frequency is a dominant oscillation … within a certain frequency band” – to be circular, unclear, and unconvincing. What does it mean for an oscillation to be “a dominant oscillation … within a certain frequency band”? A well-defined instantaneous phase exists only for a waveform that is sufficiently oscillation-like. Is the argument here that oscillations exist? That brain oscillations are not colored noise? This seems pretty self-evident. It is also hard to imagine what could be meant by the claim that “there is no evidence for the existence of different frequency domains” in rodents.

Author’s response: No, the argumentation is not circular. The argument is that phase of a task relevant oscillation from a specific source becomes distorted due to the superposition with other oscillations in a compound broad band signal (like the EEG or MEG). I have revised the first paragraphs of Section 1 to clarify this point.

Because this really is a crucial issue, let me explain here and start with citing my central argument (Introduction, Section 1): (i) ‘The central argument here is that phase can establish its impact – physiologically as well as mathematically – only, if it is associated with a task relevant oscillation with a distinct frequency (which may have even a large jitter in instantaneous period, for an illustration see Fig. 4). The reason is that in a broad band, comprising many frequencies, the phase of a task relevant oscillation will be distorted (due to superposition of these frequencies as is the case in the raw EEG/MEG signal.’ To avoid this problem, different methods are used to select task relevant oscillations. Based on these methods, numerous studies have shown that phase plays a crucial role for perception, brain communication, cognition and most importantly for cross frequency coupling. All of these findings nicely demonstrate the frequency specificity of oscillations. Or in other words, empirical findings showing that phase is meaningful also document the existence of oscillations with distinct frequencies (also termed ‘center frequencies’ in the ms). In Section 3, where I review studies on cross frequency coupling, I emphasize (ii) the crucial role of phase for coupling and its function for cognition (e.g., for episodic encoding). From these two facts, a mathematical fact (i) and empirical finding (ii) it follows (by necessity) that a task relevant oscillation has a certain frequency that is functionally relevant. What I am interested in – and what is the topic of the ms – is just the relationship between the frequencies of task relevant oscillations.

In the revised ms, I have elaborated this argumentation in Section 1, particularly with respect to (i).

With respect to the use of the term frequency domain, please see Section 2.

5)      It’s unclear to me why the manuscript uses the term “amplitude (envelope) modulation” in place of “phase-amplitude coupling”, a more specific and commonly-used term. The most obvious interpretation of the term “amplitude modulation” is amplitude comodulation, or the covariation in amplitude between two oscillations at different frequencies. In addition, there are several misconceptions about phase-amplitude coupling in the manuscript.
a.      First, the idea that the coupling of spiking or multi-unit activity to an ongoing oscillation counts as phase-amplitude coupling is against the entire spirit of defining cross-frequency coupling as distinct from rhythmicity. There are many quantities that covary with the phase of rhythms in the brain and body – this is, indeed, how and why most of these rhythms are defined and identified in the first place. To claim that all of these phenomena are examples of phase-amplitude coupling reduces the term to a vacuous generality. Phase-amplitude coupling – like all forms of cross-frequency coupling – requires two rhythms to be coupled to each other. An arrhythmic phenomenon like spiking is just a quantity that is rhythmically modulated.
b.      Secondly, respiratory sinus arrhythmia is an example of phase-frequency coupling – the phase of the respiratory rhythm modulates the frequency of the heartbeat. It is not phase-amplitude coupling, by any definition of phase-amplitude coupling that is specific enough to remain sensible. The length of the R-R interval is not the amplitude of anything.

Author’s response: I prefer to use the term ‘amplitude (envelope)’ coupling to emphasize that ‘amplitude’ simply refers to power. I am doing this to avoid the confusion between the terms instantaneous amplitude, amplitude and power. Power always is a measure referring to the envelope of a signal, whereas the term amplitude is ambiguous whether meaning instantaneous amplitude (the amplitude at a particular phase of the signal) or power.

Ad a) Yes, that’s true if the requirement is that both signals are rhythmic. I have changed the respective sentences and speak of an impact of phase that is similar to phase amplitude coupling in a formal sense.

Ad b) No! One can also speak of a signal in a mathematical and abstract sense. That’s what I say in the ms. In this abstract sense the duration of the R-R interval can of course be considered the magnitude (or amplitude of a rhythmic) signal. This is also evident in the fact that the HRV signal is frequency analyzed, which is a commonly used method in a large variety of studies.

6)      In the revised manuscript, in Section 3.3, the manuscript now lists the types of possible cross-frequency coupling. However, there is still no attempt made to justify the claim that “phase is the driving force for coupling”. Not that this claim is wrong – but the paper neither explains nor provides evidence for the claim. It would be great if the manuscript clearly stated what this could mean, and whether or not there is evidence for it.

Author’s response: This is really a minor point, but I have changed the respective sentence to ‘phase plays a crucial role for coupling’.

7)      The idea that the relationship between amplitude and period gives rise to the 1/f spectrum of brain electrical activity is an interesting one. But there is little discussion of whether all the various mechanisms of brain oscillations exhibit the same relationship between amplitude and period on a cycle-by-cycle basis. There is also no attempt to explain why the proportionality between amplitude and period should be constant across oscillations of different frequencies and mechanisms. The author should also make contact with the literature discussing the relationship between scale-free dynamics and oscillatory coupling (He et al, Neuron 2010; Pittman-Polletta et al. Biological Reviews 2013) Finally, the corresponding figure is erroneous – if amplitude and period are linearly related, then amplitude and frequency are already reciprocally related (not just negatively correlated).

Author’s response: The amplitude period covariation is just used as an example to illustrate that frequency jitter may have a physiological meaning and not necessarily is noise. I do not want to go deeper into this issue, because it is not relevant for the basic argumentation in the ms.

With respect to the Figure, I cannot see what is wrong. I think your phrasing is wrong. The right phrasing is: Both, amplitude and period on the one hand and amplitude and frequency on the other hand are linearly related. But because frequency and period are reciprocally related we have a positive correlation in one case and a negative correlation in the other case.

8)      The manuscript states “brain and body oscillations obey the same coupling principles and can be described by a single system” as if this were news. But any two oscillatory subsystems of a larger system can display the kinds of relationships between amplitude and phase that the author describes – this is part of why these measures of cross-frequency coupling are useful, and why they are already being applied to study the relationships between brain and body oscillations. Furthermore, the fact that brain and body oscillations are part of a single system is perhaps worth emphasizing in contrast to persistent dualistic notions of brain and body, but it is not a discovery or a hypothesis – it is self-evident that brains are contained in, constrained by, and interact with the “bodies” of the biological organisms of which they are a part.

Author’s response: This is a strange comment. I present an algorithm that allows to predict (successfully as I think) the frequency of body oscillations from that of brain oscillations and based on this I argue that brain and body oscillations can be described by a single system and the Reviewer says I make this statement ‘as if this were news’. Where is the reference to work showing that this is already known/described? This is again an example, where I think that the Reviewer does not see/follow my central arguments. If the Reviewer means that form an ‘every day’ or ‘wholistic’ view brain and body are part of a single system, this indeed would be a trivial statement. But instead of a naïve every day view I present an algorithm that describes and explains why brain and body oscillations are coupled.

9)      In the discussion of brain and body oscillations, the manuscript omits recent findings on respiratory-related oscillations (Tort et al. Scientific Reports, 2018). One of the major points made in the literature on RROs is that respiratory frequency varies across multiple classical frequency bands (delta and theta), and can thus be conflated with them. This seems to conflict with the central hypothesis of the paper.

Author’s response. No, you are wrong, this does not conflict with the central hypotheses, because of two reasons: (i) on the long run respiratory frequency is much slower than delta and theta and thus cannot be conflated with them. (ii) For a short time period, a transient 1:1 relationship may arise. But 1:1 is the trivial part of the binary hierarchy where frequency m*I = n for I = 1, 2, 4, 8…..

10)     Section 3.2 makes little sense.

Author’s response: Section 3.2 makes little sense? This section is central for my arguments regarding the meaning of phase and the frequency relationship between oscillations. Please see my response to your Comment 4.

a.      The author’s discussion about “temporal precision” is very unclear. What is it that operates “at the temporal precision of the slow frequency” in phase-amplitude coupling? Is the author talking about the timescale on which coupling evolves? The timing of spiking? If the former, it’s experimentally unclear at what timescales cross-frequency coupling is engaged and disengaged, and theoretically unconstrained. If the latter, the author needs to discuss more carefully what the limits on spiking precision imposed by the two coupling regimes are, and where these limits come from.

Author’s response: The argument is that only phase is a measure that is directly related to time, whereas power (envelope) is only indirectly related to time, namely to that of the phase.

b.      The idea that phase coupling can be established at different phase angles, while phase-amplitude coupling cannot, is patently false. There are many examples in the literature of high-frequency oscillations coupling to different phases of low frequency oscillations.

Author’s response: No, I could not find any sentence in the ms, where I say that phase-amplitude coupling cannot be established at different phase angles! Nonetheless, I have erased the following sentence, which may have been misleading: ’Phase coupling can be established at different phase angles. Different networks can, thus, synchronize with the same m:n ratio, but at different phase angles (e.g., one network is in counterphase relative to another network) without interference (Canolty, 2010).

c.      In the second paragraph, the author starts discussing observations of phase-phase coupling in EEG, and states “one of the basic results” is that phase-phase coupling increases in tasks with high cognitive demands. He then states “another basic results stems from the 1:2 ratio between neighboring frequencies”. I cannot see what this basic result could be. The only thing I can conclude is that the author intends to suggest that all observations of rhythms at “about” harmonic frequencies involve phase-phase coupling. Given the highly nonstationary nature of brain oscillations, with fluctuations in frequency and phase occurring from cycle to cycle, much more evidence is required than simply a “1:2 ratio” between oscillations to demonstrate phase-phase coupling. The author, however, goes on to list a number of “binary multiple frequency ratios”, while also listing non-binary frequency ratios.

Author’s response: I cannot see what is wrong here. I simply review findings showing that a 1:2 frequency relationship is commonly reported.

d.      The idea that theta-gamma coupling between frequency bands of 4-8 and 30-50 Hz “is consistent with a 1:8 coupling ratio” drives home the unnecessary and imprecise nature of the paper’s central hypothesis. If the frequency bands within which oscillations occur is broad enough to allow oscillations to assume both binary ratios and golden mean ratios, as well as ratios that fall into neither category, then why is it worth tying them to a precisely-defined mathematical hierarchy? There is not enough information in this paper to make it clear.

Author’s response: No, this is simply wrong. I define frequency bands on a clearcut mathematical reasoning and have ‘forbidden zones’ which are inconsistent with the binary hierarchy. These are used to evaluate/falsify the theory. See e.g., Section 4.1.

11)     Many of the points seem belabored, irrelevant, or too speculative.
a.      Section 3.1 (on phase-amplitude coupling) doesn’t require more than one illustrative example (say, Lakatos’ binary hierarchy).

Author’s response: No, I don’t agree, you are wrong. The issue is on the functional role of phase, which cannot be appreciated by simply referring to Lakato’s hierarchy. Furthermore and most importantly: What do you mean with Lakotos’ BINARY hierarchy. You are wrong, I checked again, in his original paper (Lakatos et al., 2005) the word binary hierarchy never is used!

b.      In Section 3.1, the discussion of phase precession is an entirely unnecessary detail, and it and the associated figure should be omitted.

Author’s response: It is omitted. The Reviewer confuses the original with the revised ms. In the latter phase precession is no longer mentioned and the respective part in the Figure removed.

c.      In Section 3.2, the tedious listing of reports of “binary multiple frequency ratios” along with non-binary multiple frequency ratios can and should be condensed.

d.      In general, the evidence supporting the main claims could be summarized much more succinctly.

e.      The binary frequency hypothesis is stated in multiple places. It should be stated once, early on, and referred to later only minimally.

Author’s response: I have erased the detailed description in Section 1

f.      The phenomena described in section 5.4 are not related enough to the central hypothesis to include this section and the associated figure.
g.      Section 6.3 – “music and the binary hierarchy” – is an interesting topic. But these ideas are not really sufficiently developed here to be included.

Author’s response: Ad c,d and f,g) Sorry, these are all central issues for my argumentation.

12)     The first figure is unnecessarily complicated – the “asymmetric oscillation which may be induced by the slope of another very slow oscillation” is not important in the text, not central to the paper’s main point, and so it should not appear in the figure.

Author’s response: This is just a hint that a slower oscillation may control the excitation level of neurons, which is an example of an interplay between oscillations. See e.g., Palva & Palva (2012a)
